# The MCL elderly III trial protocol: an international, randomized, open-label phase II trial to investigate the combinations of venetoclax, ibrutinib and rituximab or bendamustine, ibrutinib and rituximab in patients with treatment naive mantle cell lymphoma not eligible for dose-intensive treatment

**DOI:** 10.1186/s12885-025-14803-8

**Published:** 2025-08-25

**Authors:** Stephanie Herold, Christian Schmidt, Carlo Visco, Marie-Kristin Tilch, Anke Ohler, Michael Unterhalt, Eva Hoster, Elisabeth Hoenig, Susanne Singer, Alessandra Tucci, Christiane Pott, Marco Ladetto, Georg Hess, Martin Dreyling

**Affiliations:** 1 Department of Hematology and Medical Oncology, University Medical Center of the Johannes Gutenberg-University, Langenbeckstr. 1, Mainz, 55131 Germany; 2 Department of Medicine, III - University Hospital, Ludwig Maximilian University (LMU) Munich, Munich, Germany; 3 Institute for Medical Information Processing, Biometry, and Epidemiology (IBE), Faculty of Medicine, LMU Munich, Munich, Germany; 4 Interdisciplinary Centre for Clinical Trials (IZKS), University Medical Center of the Johannes Gutenberg-University, Mainz, Germany; 5 Division of Quality of Life in Oncology, Comprehensive Cancer Center Mecklenburg- Vorpommern (CCC-MV), Rostock, Germany; 6 Hematology and Bone Marrow Transplant Unit, Department of Engineering for Innovative Medicine, University of Verona, Verona, Italy; 7 Department of Translational Medicine, Division of Hematology, Piedmont and SCDU Ematologia, University of Eastern, Azienda Ospedaliera Santi Antonio e Biagio e Cesare Arrigo, Alessandria, Italy; 8 Division of Hematology, Spedali Civili, Brescia, Italy; 9 Department of Medicine II, University Hospital Schleswig-Holstein - Campus Kiel, Kiel, Germany

**Keywords:** Mantle cell lymphoma, Ibrutinib, Venetoclax, Elderly, Chemo-free, First-line therapy, MCL elderly III

## Abstract

**Background:**

Mantle cell lymphoma (MCL) is a rare B-cell Non-Hodgkin-lymphoma that predominantly affects elderly patients. While younger and fit patients receive an intensive first-line treatment, older or comorbid patients have limited options of chemo-immunotherapy (CIT) alone followed by anti-CD20-antibody maintenance. Targeted oral agents as Bruton`s tyrosine kinase inhibitors (BTKi, e.g. ibrutinib) - and B-cell lymphoma 2 (Bcl2) – inhibitors (e.g. venetoclax) have revolutionized the treatment especially for relapsed patients, with apparent synergistic effects. The MCL elderly III trial of the European MCL Network is an international phase II trial evaluating the efficacy of the combination of ibrutinib, venetoclax and rituximab as well as the CIT bendamustine and rituximab in combination with ibrutinib in elderly patients with untreated MCL.

**Methods:**

The primary trial objective is to evaluate efficacy in both treatment arms as measured by failure-free survival at 30 months separately in both treatment arms. Secondary endpoints include progression-free survival, response rates, overall survival, adverse events as well as quality of life and impact of frailty and sarcopenia on treatment outcome through geriatric and body composition assessments via imaging. Exploratory endpoints comprise the rate of minimal residual disease negativity and kinetics of immune reconstitution.

**Current status:**

The first patient was included in May 2023, with full site activation achieved in Q1 2025. Until May 15th 2025, 75 of 150 planned patients were enrolled in 27 German and Italian trial sites.

**Discussion:**

This is the first randomized trial to exploratively compare a BTKi-Bcl2i-anti-CD20 triplet to a BTK-CIT combination in older MCL patients.

**Trial registration:**

The trial is registered on EU Clinical Trial Register (20225018089600).

**Supplementary Information:**

The online version contains supplementary material available at 10.1186/s12885-025-14803-8.

## Background and rationale

Mantle cell lymphoma (MCL) is a rare B-cell Non-Hodgkin-lymphoma (NHL) that is challenging to treat and is considered incurable with conventional treatment. Patients are predominantly elderly men, with a median age of 65 years and a male to female ratio of 3:1 [1, 2]. The disease is typically generalized at diagnosis with frequent nodal, bone marrow, and extra-nodal manifestations [3]. MCL is characterized by the translocation *t(11;14)* which leads to overexpression of Cyclin D1, resulting in a growth advantage of the malignant cell [4]. The MCL international prognostic index (MIPI) discriminates 3 risk groups with varying prognosis [5–7]. Biologic risk factors include *TP53* mutation/deletion, blastoid/pleomorphic variant and the expression of Ki67 > 30% [8–10]. During disease evolution, secondary genetic alterations, particularly *TP53* mutations, commonly emerge, driving a progressively aggressive disease biology and resistance to conventional chemotherapies [11]. Consequently, improvements in first-line treatment strategies have likely been a major contributor to the substantial therapeutic progress observed in recent years [12].

Current treatment concepts are basically selected on patient’s age and performance status. Until recently, younger and medically fit patients received an intensive first-line treatment with a high-dose cytarabinoside-containing induction that was followed by consolidative high dose therapy including autologous stem cell transplantation (ASCT) and rituximab maintenance [13–15]. With the results of the TRIANGLE and the ECOG-ACRIN EA4151 trial, ASCT should no longer be offered to patients on a routine basis, as no benefit could be proven from dose intensification when ibrutinib is added to induction and as maintenance [16–19].

For elderly patients, less intensive chemo-immunotherapy (CIT) options are established, of which bendamustine-rituximab (BR) is most frequently used, alongside rituximab-cyclophosphamide-doxorubicin-vincristin-prednisone (R-CHOP) or VR-CAP (rituximab-cyclophosphamide-doxorubicin-bortezomib-prednisone) [20–22]. Maintenance with rituximab is regularly applied [23]. The addition of ibrutinib to BR improved median progression free survival (PFS) from 53 to 81 months in the SHINE trial without a difference in overall survival (OS) [24] and similar results have been obtained for acalabrutinib in the ECHO trial [25]. The not yet fully published data of the ENRICH trial even showed superiority of ibrutinib combined with rituximab (IR) compared with CIT alone. Noteworthy, this benefit was driven by the R-CHOP group, not the BR group. However, patients treated with a chemo-free approach reported a higher quality of life [26].

Whether an MCL patient is considered elderly is often left to the discretion of the attending physician. However, elderly patients of a similar age may be extremely heterogeneous considering comorbidities, functional capacities, psychological and physical performance status. Geriatric assessment has been shown to predict severe treatment-related toxicity and overall survival (OS) in patients with various malignancies, and is now recommended by major scientific societies [27–29]. The application of geriatric assessment in MCL patients is of particular interest due to the promising results observed with targeted agents, showing good tolerability and a favorable safety profile. However, no data from clinical trials in MCL patients are currently available.

At relapse, Bruton’s tyrosine kinase inhibitors (BTKi) have shown high efficacy superior to the re-use of chemotherapy, and ibrutinib represents the therapeutic standard of care [30]. Furthermore, venetoclax, a first in class Bcl-2 inhibitor, has demonstrated high response rates in relapsed MCL, but the duration of remission after monotherapy was limited thus prompting the exploration of combination therapies [31–33]. The results of the randomized SYMPATICO trial comparing ibrutinib to ibrutinib-venetoclax in relapsed MCL subsequently proved a significant benefit for the combination over single agent use with good tolerability [34]. The addition of the anti-CD20 antibody obinutuzumab to this combination in a phase I/II trial (OASIS) with infinite ibrutinib treatment achieved promising results in relapse and, with very high minimal residual disease (MRD) negativity, also in first line [35, 36]. The subsequent phase II trial, OASIS II, showed higher MRD negativity of this combination as compared to obinutuzumab and ibrutinib alone in a preliminary analysis and is currently recruiting [37].

In the past, based on early experiences with BTKi in relapsed MCL, treatment cessation of ibrutinib was generally not recommended due to the risk of relapse. However, a substantial number of patients do not tolerate long term treatment due to side effects such as cardiac toxicity. As a result, time-limited approaches have gained attention, particularly for patients receiving first-line therapy, and contrast with the concept of infinite duration explored in SHINE, ECHO and ENRICH [24–26].

Hence, a chemo-free, fixed duration treatment with ibrutinib-venetoclax and an anti-CD20 antibody may effectively challenge current CIT and even outstand combinations of CIT with BTKi as recent trials pronounce the benefit of novel agents in MCL treatment. With the results of TRIANGLE being reserved for the young and fit patient cohort, novel first-line combinations are of special interest for the elderly and unfit patients [17]. The European MCL Network therefore initiated the phase II MCL elderly III trial to evaluate efficacy and safety of the combination of ibrutinib, venetoclax and rituximab as well as the CIT bendamustine and rituximab in combination with ibrutinib.

## Methods

### Study design

This international phase II, multicenter, prospective, open label, randomized trial investigates the combination of ibrutinib, venetoclax and rituximab versus the CIT regimen of BR in combination with ibrutinib in older patients with previously untreated MCL. A central block randomization will be used for allocation of patients to both arms in a 1:1 ratio. Stratification is performed according to country and MIPI risk groups.

### Patients - eligibility

For this trial, adult patients (≥ 60 years and no candidates for dose-intensive treatment) must have histologically confirmed diagnosis of MCL. The term “dose-intensive treatment” refers to regimens incorporating high-dose cytarabine and/or ASCT. Eligibility for dose-intensive treatment was determined at the discretion of the treating physician and was not based on pre-specified objective thresholds. Clinical factors commonly taken into account included comorbid conditions and overall ability to tolerate intensive cytotoxic therapy or ASCT.

Further inclusion criteria comprise: untreated stage II-IV, Eastern Cooperative Group (ECOG) performance status of ≤ 2 (if not disease related), at least 1 measurable tumor lesion > 1.5 cm x > 1.0 cm, and/or bone marrow infiltration, adequate hematopoietic reserve (platelets ≥ 75.000 cells/µl, absolute neutrophil count ≥ 1000 cells/µl) as well as an appropriate liver (transaminases (AST and ALT) ≤ 3 x upper limit of normal (ULN), total bilirubin ≤ 2 x ULN except Gilbert´s Syndrome) and renal function (measured or calculated eGFR ≥ 50 mL/min and creatinine ≤ 2 mg/dL).

Patients are excluded for any of the following reasons: Prior organ, bone marrow, or peripheral blood stem cell transplantation, serious cardiovascular disease, history of severe bleeding, central nervous system involvement, known infection with seropositive HIV or active hepatitis B (when positive for HBsAG) or C (when elevation of transaminases, coagulopathy or active virus replication is present) viruses or previous malignancies within the last 3 years except for basal cell skin cancer, prostate cancer in remission with prostate-specific antigen within normal range or in situ uterine cervix cancer.

### Treatment

We investigate the efficacy and safety of the combination of BR and ibrutinib for 6 cycles followed by maintenance of rituximab and ibrutinib for 2 years and the chemotherapy-free combination of venetoclax, ibrutinib and rituximab for 6 cycles followed by a maintenance treatment for 2 years. The investigational medicinal products (IMPs) for this trial are ibrutinib and venetoclax. Auxiliary medicinal products (AMPs) are rituximab (both arms) and bendamustine. Each treatment cycle consists of 28 days.

**Fig. 1:**
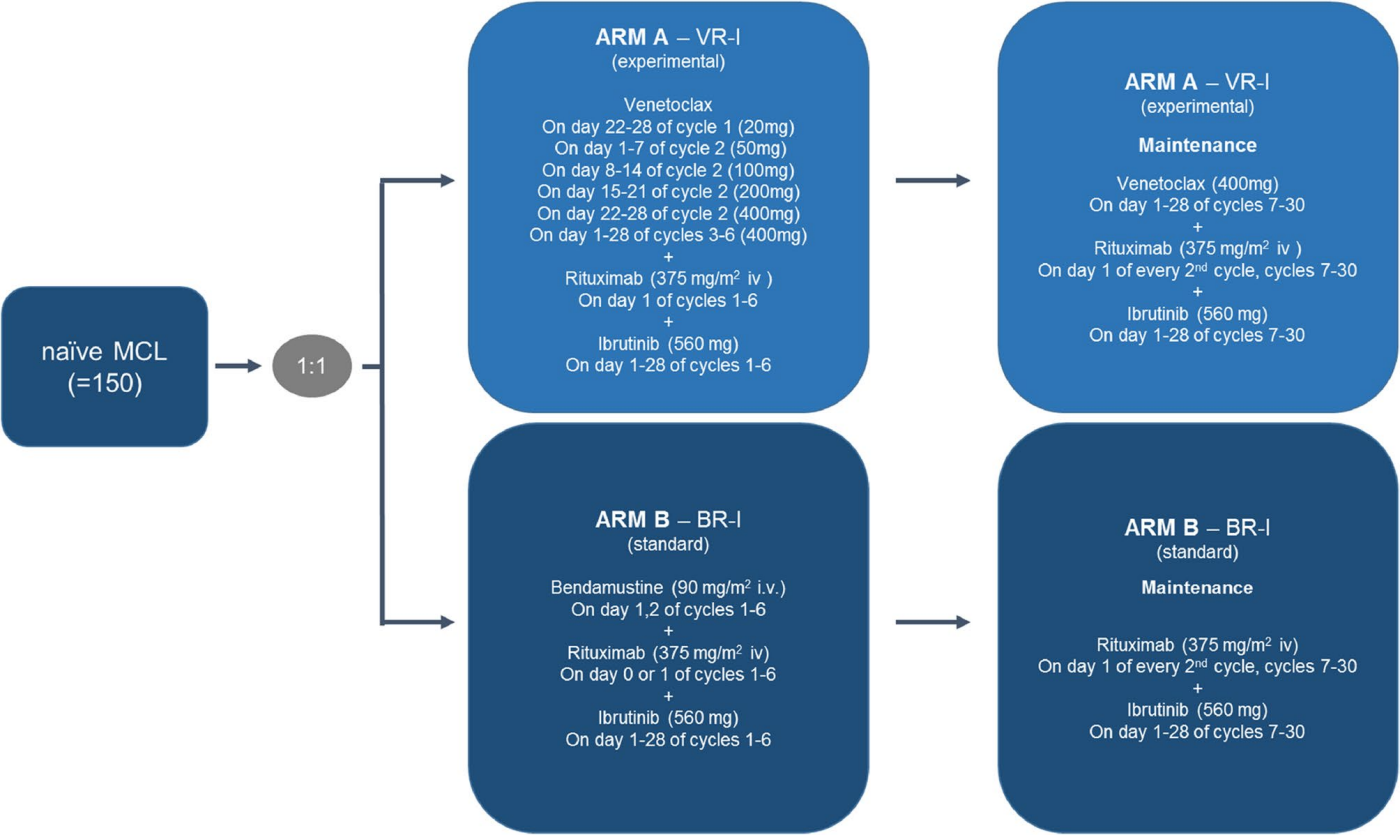
Study design. Arm A shows the experimental arm with treatment consisting of 6 induction cycles followed by a maintenance of another 24 cycles of the full combination. Arm B comprehends the standard arm consisting of 6 induction cycles followed by a maintenance of another 24 cycles of Rituximab and Ibrutinib without Bendamustine. VR-I: Venetoclax/Rituximab/Ibrutinib, BR-I: Bendamustine/Rituximab/Ibrutinib

Patients enrolled and randomized to arm A receive 375 mg/m^2^ intravenous (iv) rituximab on day 1, along with 560 mg ibrutinib orally on day 1–28 for a maximum of 6 cycles. Venetoclax is initiated orally with 20 mg (Cycle 1, day 22–28) and gradually ramped-up up to a target dose of 400 mg over 5 weeks. After ramp-up, the final dose of 400 mg is administered at day 1–28 for cycles 3 to 6 (Fig. 1). Unless there is no clear evidence of disease progression patients can continue for maintenance (cycle 7–30) with 375 mg/m^2^ iv rituximab at day 1 of every second cycle plus 560 mg ibrutinib on day 1–28 and 400 mg venetoclax on day 1–28.

Patients enrolled and randomized to arm B will receive 90 mg/m^2^ iv bendamustine on days 1 and 2, along with 375 mg/m^2^ iv rituximab on day 1, and an additional 560 mg of ibrutinib daily from days 1–28. The regime will be repeated for a maximum of 6 cycles. Unless there is no clear evidence of disease progression patients can continue for maintenance (cycles 7–30) with 375 mg/m^2^ iv rituximab on day 1 of every second cycle plus 560 mg ibrutinib on days 1–28.

### Study procedures

A diagnostic MCL biopsy sample must be sent to a national reference pathology laboratory for confirmation and assessment of biologic risk factors, including Ki67 and TP53 mutation analysis. Disease dissemination is evaluated using computed tomography (CT) scans according to the Lugano criteria, positron emission tomography (PET) scans, and bone marrow biopsy. At defined time points, samples for MRD and immune reconstitution will be collected and analyzed. Quality of life will be assessed by validated questionnaires before the therapy start and during the treatment (European Organization for Research and Treatment of Cancer quality of Life Core questionnaire, EORTC QLQ-C30, and its module for high-grade non-Hodgkin lymphoma, EORTC QLQ-NHL-HG29) [38, 39] A simplified geriatric assessment consisting of the items Activities of Daily Living (ADL) [40], Instrumental Activities of Daily Living (IADL) [41] and Cumulative Illness Rating Scale for Geriatrics (CIRS-G) [42] is performed prior first dose. Body composition and evaluation of sarcopenia is evaluated in an augmented reality-based, semiautomatic approach based on the CT scans at screening and end of treatment (EOT). Regular investigator independent staging has been integrated into the patient surveillance including a mandatory PET-CT at end of induction. Follow up visits after the end of maintenance are planned every 6 months. Enrolment of trial patients into the European Mantle Cell Lymphoma (EMCL) registry is highly recommended for long term follow-up.

### Outcome measures and endpoints

We investigate the efficacy and safety of the combination of BR and ibrutinib for 6 cycles followed by maintenance of rituximab and ibrutinib for 2 years and the chemotherapy-free combination of venetoclax, ibrutinib and rituximab for 6 cycles followed by a maintenance treatment for 24 months.

The primary endpoint is efficacy as measured by failure free survival (FFS) at 30 months from randomization among all evaluable patients in both treatment arms determined by CT scan evaluation following Cheson criteria [43]. Patients who achieve a complete or partial remission and remain alive without disease progression at 30 months post-randomization are classified as having achieved FFS at 30 months. In contrast, patients with stable or progressive disease at the end of induction, as well as those who experience death from any cause before or during the 30-month assessment, are classified as not achieving FFS at 30 months.

Secondary endpoints comprise other efficacy endpoints such as continuous FFS from randomization, PFS, OS, complete remission (CR) rate and overall response rate (ORR) as well as safety endpoints such as frequency of adverse events (AEs) and quality of life.

Simplified geriatric assessment will be correlated to treatment toxicity and efficacy in order to investigate the impact of frailty on treatment outcome and whether vulnerable patients may particularly benefit from a chemo-free approach.

Body composition assessment and sarcopenia will be assessed in an augmented reality-based, semiautomatic approach analogous to the work of Müller et al. at diagnosis and at EOT, assuming that due to the advanced age it might already be present at diagnosis and is likely to deteriorate during therapy [44]. Its assumed prognostic impact on therapy outcomes will be evaluated with a special focus on the impact of the chemo-free treatment in comparison to the standard-of-care CIT. The image data is initially collected and stored locally at the respective site, and required scans are digitally transferred in a pseudonymized way to the University Medical Center Mainz for evaluation.

### Exploratory endpoints and translational program

The accompanying scientific program will evaluate the correlation between molecular remission or conversion and clinical response. MRD will be determined at screening, after induction, during maintenance and during follow up. MRD will be determined by assessment of circulating lymphoma cells using high-throughput sequencing targeting clonal immunoglobulin rearrangements. Sensitivity of MRD detection will achieve 10^−06^. In an exploratory analysis, ctDNA from plasma samples will be tracked by targeted capture sequencing.

Central pathological review includes mutation pattern analysis on biopsy materials for genetic subclassification with NGS (next-generation sequencing) methods.

Immune reconstitution is assessed by flow cytometry. Using four multicolor flow cytometry panels (9–10 colors per panel) incorporating markers such as CD45, CD45RA/RO, CD3, CD4, CD5, CD8, CD10, CD15, CD16, CD19, CD20, CD27, CD56, CD57, CD64, CD69, NKG2A, Slan, SIRPalpha, TREM-1, PD-1, VEGFR, IgM, IgD, TCR alpha/beta, and TCR gamma/delta, dynamic changes in T cells, NK cells, B cells, monocytes, and granulocytes are analyzed. These markers enable the identification and detailed characterization of immune cell subpopulations. This allows for precise monitoring of immunological changes at the cellular level throughout disease progression and therapy. Investigating immune reconstitution in the context of CIT versus a chemo-free regimen is of particular interest, as it may offer insights into differential immune system responses to these treatment approaches.

### Statistics

The primary analysis population is the intention-to-treat (ITT) population (all randomized subjects). Per protocol (PP) analyses will additionally be performed for secondary efficacy analysis. The safety population comprises all subjects who received at least one dose of trial treatment.

The frequency and percentage of 30-month FFS will be reported by trial arms among all evaluable patients. Separately for each trial arm, an exact binomial hypothesis test will be performed using the null hypothesis 30-month FFS ≤ 60%; the alternative hypothesis is expressed as 30-month FFS > 60%, aiming to accept a value superior to that observed with R-CHOP + R-maintenance in the previous European MCL Elderly trial [45], which represented the most favorable and comparable benchmark available at the time of this trial’s design. The significance level will be set to 10% one-sided, accounting for the phase II design with limited sample size. A one-sided lower-bounded 10%-exact confidence interval will be calculated for the estimated 30-month FFS probability in each treatment arm.

The secondary efficacy endpoints will be evaluated with descriptive statistical methods separately in each treatment group. Depending on the data type, absolute and relative frequencies, median, interquartile range (IQR), range, or Kaplan-Meier estimates will be calculated. For probabilities estimated by relative frequencies or the Kaplan-Meier method, two-sided 95% confidence intervals will be reported.

FFS, PFS and OS as time-to-event variables will be exploratively compared between the two treatment arms using Cox regression. In unadjusted analyses and analyses adjusted for MIPI score and/or Ki-67 index (≥ vs. <30%), the hazard ratio between the two treatment arms will be estimated along with two-sided 95% confidence intervals. These results can only be interpreted as hypothesis generating, since the statistical power for a confirmatory evaluation will be unacceptably low. If, for example, BR-I achieves a 30-month FFS rate of 70%, corresponding to a hazard ratio of 0.70 in comparison to R-CHOP + R, 67 evaluable patients per group give rise to a power of only 52% to detect a 12% improvement to 82% 30-months FFS with VR-I (hazard ratio 0.56) by a two-sided logrank-test with alpha = 0.05. With 67 evaluable patients per group, the trial will achieve 80% power to detect an improvement of 16% and 15% for VR-I to 86% and 90% in comparison to 70% and 75% assumed with BR-I, respectively. The huge and unrealistic corresponding hazard ratios of 0.42 and 0.37 to be detected with 80% power in the direct comparison underline the phase 2 nature of this trial that will probably not definitively answer the question of superiority of VR-I over BR-I.

QoL will be analyzed using mixed-models regression for the a priori defined primary QoL domains Global QoL (the EORTC QLQ-C30), and secondary QoL domains Physical, condition/fatigue, Symptom burden (EORTC QLQ-NHL-HG29). The remaining QoL domains will be descriptively analyzed per trial arm and the differences per scale will be checked against a threshold of 10 points (clinical relevance).

### Planned sample size and trial duration

Allowing for 10% dropouts, an estimated sample size of 75 per group gives 90% power to detect an improvement by 15–75% 30-month FFS for each treatment arm when compared to the outcome with R-CHOP + R maintenance (60% 30-month FFS) using one-sided binomial tests with significance level 0.10. Therefore, it is planned to enroll and randomize 150 subjects.

Based on enrollment projections, the duration of this study is estimated to be 66 months (5.5 years). Recruitment is ongoing.

### Trial status

The study is recruiting patients from 36 trial sites in Germany and Italy. The first study site was activated in Q2/2023 with a current recruitment of 75 of 150 patients; full site activation achieved in Q2/2025.

## Discussion

### Current status, outlook and potential impact of the data

Currently, chemotherapy-based regimen are the established treatment of choice for MCL patients requiring treatment at all age cohorts. For older patients, CIT followed by R-maintenance represents the current standard of care, with BR-acalabrutinib or even IR expected to become a new standard soon [25, 26]. Especially elderly patients experience chemotherapy-related toxicities and the long-term outcome leaves substantial room for improvement. Therefore, new treatment options with improved tolerability and efficacy are necessary.

At the time of trial design, we expected the combination of BR-ibrutinib to become the optimal available standard for elderly patients with MCL. Similar to BR-ibrutinib and IR evaluated in SHINE and ENRICH, the addition of novel agents enhances the current standard while a chemo-free regimen may demonstrate a favorable side effect profile. Although ibrutinib was the first approved BTKi in relapsed MCL at the time this trial was designed and thus selected for both treatment arms, it is important to consider the implications of future results in the context of newer covalent (acalabrutinib and zanubrutinib) and non-covalent (pirtobrutinib) BTKi [25, 46, 47]. The ECHO trial led to FDA and EMA approval of acalabrutinib in combination with BR for older, untreated MCL patients just recently [25]. Zanubrutinib is FDA-approved after at least one prior therapy based on the results of a phase II study that also showed a favorable safety profile of zanubrutinib, particularly with regard to cardiovascular toxicity [48], consistent with earlier studies of second-generation BTKi that were conducted in patients with chronic lymphocytic leukemia [49]. The superior safety profiles of newer BTKi may influence treatment choices going forward. As such, the interpretation of our findings should take into account the evolving therapeutic landscape, where more selective BTK inhibitors may provide similar efficacy with potentially fewer adverse events.

The combination of ibrutinib, venetoclax and an anti-CD20 antibody has shown promising efficacy and favorable toxicity as investigated in the OASIS trial [35, 37]. However, in contrast to OASIS, SHINE, ECHO and ENRICH, the MCL Elderly III is the first *randomized* prospective trial exploring a time-limited chemo-free triplet compared to an optimal chemotherapy based combination [24–26, 37]. Considering tolerability, a time-limited approach is attractive, particularly in first-line. In the SHINE and ECHO trial, the median exposure to BTKi was 24.1 and 28.6 months due to toxicity [24, 50]. Accordingly, the arm of the MCL Elderly III trial - comprising venetoclax, ibrutinib, and rituximab - is expected to demonstrate high efficacy, with the added advantage of a fixed treatment duration.

Furthermore, the OASIS II trial demonstrated a high rate of MRD negativity [37]. Given that MRD status appears to be predictive of both relapse risk and treatment outcomes, its assessment represents a valuable clinical tool. Achieving a similarly high MRD negativity rate may further support the overall feasibility of a fixed-duration treatment approach. Moreover, monitoring MRD conversion during the treatment-free follow-up phase allows for early detection of potential relapses. Furthermore, a comprehensive understanding of immune reconstitution across the different treatment arms and following treatment cessation is essential to ensure optimal safety in this vulnerable patient population. Given the well-documented T-cell depleting effect of bendamustine, comparative analysis may also reveal differential impacts of the regimen on the efficacy of subsequent T-cell engaging therapies. Long-term clinical outcomes and the course of subsequent treatments can be systematically captured through extended follow-up within the EMCL registry.

Consequently, a chemotherapy-free, time-limited regimen combining ibrutinib, venetoclax, and rituximab may represent a promising alternative to the current standard of care. Should it demonstrate superior outcomes compared to BR plus ibrutinib, these findings would support further evaluation in a phase III trial. This study is designed as a hypothesis-generating investigation, with the potential to inform the design of a future phase III trial. Notably, this study constitutes the first randomized trial to directly compare a BTKi–Bcl2i–anti-CD20 triplet with a BTKi–containing CIT regimen.

## Supplementary Information

Supplementary Material 1.

Supplementary Material 2.

## Data Availability

No datasets were generated or analysed during the current study.

## Abbreviations

ADL: Activities of Daily Living
AE: Adverse event
ASCT: Autologous stem cell transplantation
AMP: Auxiliary medicinal product
BR: Bendamustine Rituximab
Bcl2: B-cell lymphoma 2
BTKi: Bruton tyrosine kinase inhibitors
CIRS-G: Cumulative Illness Rating Scale for Geriatrics
CIT: Chemoimmunotherapy
CR: Complete remission
CT: Computed tomography
ECOG: Eastern Cooperative Group
EMCL: European Mantle Cell Lymphoma
EORTC: European Organisation for Research and Treatment of Cancer
FFS: Failure free survival
IADL: Instrumental Activities of Daily Living
IQR: Interquartile range
IR: Ibrutinib Rituximab
ITT: Intention-to-treat
MCL: Mantle cell lymphoma
MIPI: MCL international prognostic index
MRD: Minimal residual disease
NGS: Next-generation sequencing
NHL: Non-Hodgkin-lymphoma
ORR: Overall response rate
OS: Overall survival
PET: Positron emission tomography
PFS: Progression free survival
PP: Per protocol
QoL: Quality of life
R: Rituximab
ULN: Upper limit of normal
