# Supplementary material for: The MCL elderly III trial protocol: an international, randomized, open-label phase II trial to investigate the combinations of venetoclax, ibrutinib and rituximab or bendamustine, ibrutinib and rituximab in patients with treatment naive mantle cell lymphoma not eligible for dose-intensive treatment
Source: BMC Cancer. 2025 Aug 25;25:1370. doi: 10.1186/s12885-025-14803-8 (PMC12376325; doi:10.1186/s12885-025-14803-8)
Supplement: Supplementary file 2 — Supplementary Material 2. [file 12885_2025_14803_MOESM2_ESM.docx]

CLINICAL TRIAL PROTOCOL

Venetoclax in combination with the BTK inhibitor Ibrutinib and Rituximab or conventional chemotherapy (Bendamustine) and Ibrutinib and Rituximab in patients with treatment naive Mantle Cell Lymphoma not eligible for high dose therapy

**- MCL Elderly III -**

(Mantle Cell Lymphoma Elderly III-Trial)

Eudra CT 2020-002935-30

EU CT 2022-501808-96-00

Sponsor trial code 20-01434

Version 1.1

Date 2023-01-05

| **Sponsor** | **Coordinating investigator** |
| --- | --- |
| University Medical Center of the  Johannes Gutenberg-University Mainz  represented by the executive board of the University  represented by the scientific member of the executive board  Univ.-Prof. Dr. U. Förstermann | Institution Medizinische Klinik III Klinikum der Universität LMU München  Name Univ.-Prof. Dr. med. Martin Dreyling  Address Marchioninistrasse 15, 81377 München  Phone +49 (0)89 / 4400-72202  Fax +49 (0)89 / 4400-72201  e-Mail [Martin.Dreyling@med.uni-](mailto:Martin.Dreyling@med.uni-) muenchen.de |
| **Operating institution of Sponsor** | **Biometrician** |
| Institution Universitätsmedizin Mainz delegated to  Name Univ.-Prof. Dr. med. Georg Hess  Address III. Medizinische Klinik und Poliklinik Langenbeckstr. 1, 55131 Mainz  Phone +49 (0)6131 / 17 5040  Fax +49 (0)6131 / 17 475040  e-Mail georg.hess@unimedizin-mainz.de | Institution Institut für Medizinische Informationsverarbeitung, Biometrie und Epidemiologie (IBE),  LMU München  Name Univ.-Prof. Dr. rer. biol. hum., Dipl.-Math Eva Hoster  Address Marchioninistr. 15, 81377 München  Phone +49 (0)89 / 4400-77496  Fax +49 (0)89 / 440077491  e-Mail ehoster@ibe.med.uni-muenchen.de |

This protocol is confidential information and intended solely for the guidance of the clinical trial. It must not be disclosed to third parties not associated with the clinical trial or used for any other purpose without the prior written consent of the sponsor.

**DOCUMENT HISTORY**

| **Version** | **Date** | **Comments/Summary of Changes** |
| --- | --- | --- |
| 1.0 | 2022-08-05 | First Submission |
| 1.1 | 2023-01-05 | Including pulmonary function test. Correcting typo. Clarification of sampling schedule. |


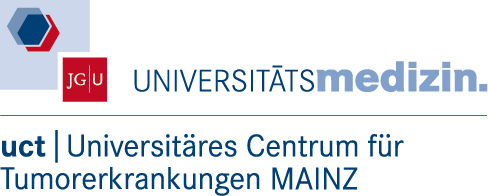

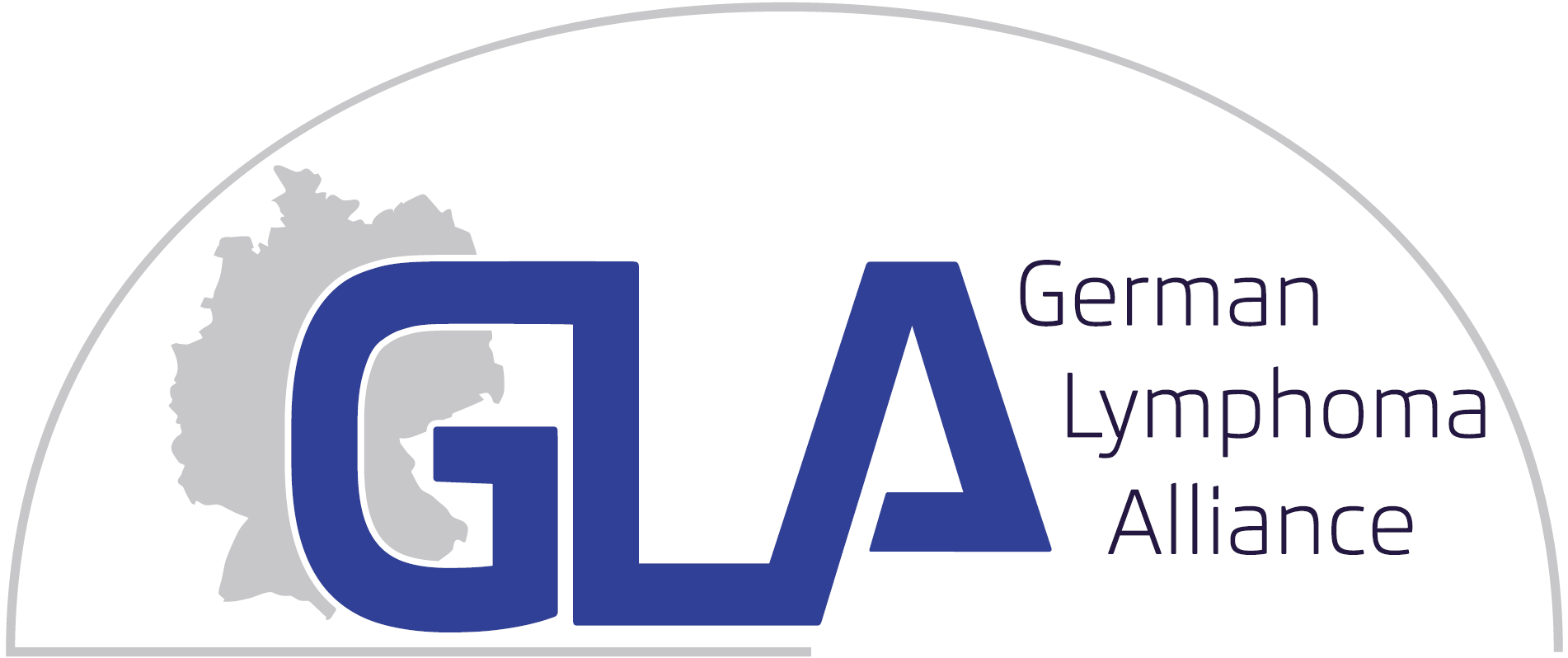

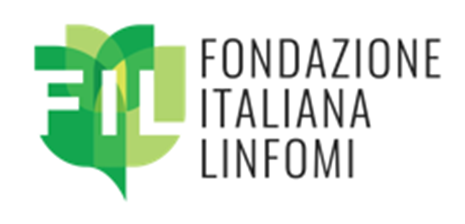

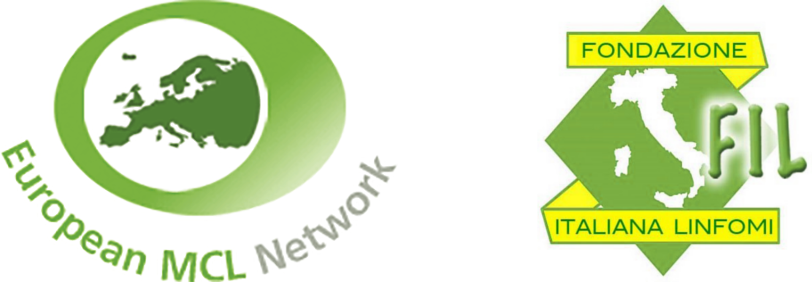


# SIGNATURES

The present trial protocol was subject to critical review and has been approved in the present version by the persons undersigned. The information contained is consistent with:

- The current risk-benefit assessment of the investigational medicinal product.
- The moral, ethical, and scientific principles governing clinical research as set out in the Declaration of Helsinki and the principles of GCP.
- This clinical study is conducted in accordance with the study protocol, ICH guidelines, GCP principles, the Declaration of Helsinki, and applicable laws and regulations.

**Local Delegate of the Institution of the Sponsor (Prof. Dr. G. Heß)**

_________________ _____________________________________

Date Signature

**Coordinating Investigator (Prof. Dr. M. Dreyling)**

_________________ _____________________________________

Date Signature

**Biometrician (Prof. Dr. E. Hoster)**

_________________ _____________________________________

Date Signature

DECLARATION OF INVESTIGATOR

I have read the following trial protocol and I confirm that it contains all information to conduct the clinical trial accordingly. I pledge to conduct the clinical trial according to the protocol.

I will enroll the first subject only after all ethical and regulatory requirements are fulfilled. I pledge to obtain written consent for trial participation from all subjects.

I know the requirements for accurate notification of serious adverse events and I pledge to document and notify such events as described in the protocol.

I will conduct the trial in compliance with the protocol, GCP and the applicable regulatory requirements.

**Investigator**

Name

Address

Phone

Fax

E-Mail

_________________ _____________________________________

Date Signature

**Writing committee** Primary Investigator

Univ.-Prof. Dr. med. Martin Dreyling, Munich

**Further members** Univ.-Prof. Dr. med. Georg Heß, Mainz

Univ.-Prof. Dr. med. Christiane Pott, Kiel

Univ.-Prof. Dr. rer. biol. hum. Eva Hoster, Munich

Dr. med. Michael Unterhalt, Munich

Prof. Dr. Carlo Visco, Verona

Prof. Dr. Marco Ladetto, Milan

Dr. rer. nat. Anke Ohler, Mainz

Prof. Dr. Susanne Singer, Mainz

Dr. med. Marie-Kristin Tilch, Mainz

Dipl. biol. Elisabeth Hoenig, Mainz

Dr. Alessandra Tucci, Brescia

**List of abbreviations**

AE Adverse event

AMG German drug law (Arzneimittelgesetz)

ANC Absolute neutrophile count

BP Blood pressure

CNS Central nervous system

CRF Case report form

CTCAE Common terminology criteria for adverse events

CTIS Clincal trial information system

CV Curriculum vitae

DMP Data Management Plan

DSMB Data safety monitoring board

EC/IEC Ethics committee/Independent ethics committee

e-CRF Electronic case report form

FSI First subject in

GCP Good clinical practice

GCP-V GCP regulation

HR Heart rate

ICH International conference on harmonization of technical requirements for registration of pharmaceuticals for human use

IMP Investigational medicinal product

IRB Institutional review board

ISRCTN International standard randomized controlled trial number

INN International nonproprietary name

IRR Infusion related reaction

ITT Intention to treat

IZKS Interdisciplinary Center for Clinical Trials

LSI Last subject in

LSO Last subject out

MedDRA Medical dictionary for regulatory activities terminology

NCI National cancer institute

PFT Pulmonary function test

QoL Quality of life

RDE Remote Data Entry

SAE Serious adverse event

SaO_2_ Peripheral oxygen saturation

SCT Stem cell transplantation

SAP Statistical analysis plan

SOP Standard Operating Procedure

SDV Source data verification

SUSAR Suspected unexpected serious adverse reaction

TLS Tumor lysis syndrome

TMF Trial master file

**Synopsis**

| **Title** | Venetoclax in combination with the BTK inhibitor Ibrutinib and Rituximab or conventional chemotherapy (Bendamustine) and Ibrutinib and Rituximab in patients with treatment naive Mantle Cell Lymphoma not eligible for high dose therapy |
| --- | --- |
| **Short title** | MCL-Elderly III |
| **Eudra CT** | 2020-002935-30 |
| **Sponsor trial code** | 20-01434 |
| **Indication** | Mantle Cell Lymphoma |
| **Phase** | II |
| **Experimental Treatment arms** | Arm A (VR-I): Venetoclax, Rituximab, Ibrutinib  Arm B (BR-I): Bendamustine, Rituximab, Ibrutinib |
| **Study medication** | Venetoclax, Ibrutinib |
| **Primary objective and endpoint** | To evaluate efficacy in both treatment arms:   - Failure-Free Survival (FFS) at 30 months |
| **Secondary objectives and endpoints** | To evaluate efficacy, safety, tolerability, and quality of life in both treatment arms:   - Failure-free survival (continuous observation) - Progression-free survival - Complete Remission rate (CR) and overall response rate (ORR: CR, PR) four weeks after the end of induction therapy - best response, time to best response, time to first response - overall survival - Overall survival of patients divided according to the geriatric categories and treatment received - Safety: adverse events, tolerability - Quality of life during induction and maintenance therapy (assessed using the EORTC QLQ-C30 and the EORTC QLQ-NHL-HG29) - Molecular remission after induction and conversion during maintenance (exploratory) - Immune reconstitution, e.g. persistence of anti-Covid19 immunity - safety and efficacy in different geriatric categories |
| **Trial design** | International, multicenter, open label, randomized phase II trial |
| **Trial population** | Key Inclusion Criteria:   - Histologically confirmed diagnosis of MCL according to WHO classification - previously untreated stage II-IV (Ann Arbor) - ≥ 60 years and not suitable for autologous SCT - At least 1 measurable lesion; in case of bone marrow infiltration only, bone marrow aspiration and biopsy is mandatory for all staging evaluations. - ECOG performance status ≤ 2   The following laboratory values at screening (unless related to MCL):   - Absolute neutrophil count (ANC) ≥ 1000 cells/µL - Platelets ≥75.000 cells/µL - Transaminases (AST and ALT) ≤3 x ULN - Total bilirubin ≤ 2 x ULN unless other reason known (Gilbert-Meulengracht-Syndrome) - Creatinine ≤ 2 mg/dL or eGFR ≥ 50 mL/min - Written informed consent form according to ICH/EU GCP and national regulations - Sexually active men with female partners of child-bearing potential potential must agree to use highly effective contraceptives   Key exclusion criteria:   - Major surgery within 4 weeks prior to first dose - Requires anticoagulation with warfarin or equivalent vitamin K antagonists (e.g. phenprocoumon) - History of stroke or intracranial hemorrhage within 6 months prior to first dose - Treatment with strong or moderate CYP3A4/5 inhibitors/inducers within 7 days before first dose and during Venetoclax and Ibrutinib intake - Any life-threatening illness, medical condition, or organ system dysfunction which, in the investigator’s opinion, could compromise the subject’s safety, interfere with the absorption or metabolism of Ibrutinib capsules, or put the study outcomes at undue risk - Vaccinated with live, attenuated vaccines within 4 weeks prior to first dose - Known CNS involvement of MCL - Known bleeding disorder (e.g. von Willebrand disease; hemophilia) - Serious concomitant disease interfering with a regular therapy according to the study protocol: - Cardiac (Clinically significant cardiovascular disease such as uncontrolled or symptomatic arrhythmias, congestive heart failure, or myocardial infarction within 6 months of Screening, or any Class 3 (moderate) or Class 4 (severe) cardiac disease as defined by the New York Heart Association Functional Classification or LVEF below LLN) - Pulmonary (e.g. chronic lung disease with hypoxemia, e.g. DLCO ≤ 65% or FEV1 ≤ 65%) - Endocrinological (e.g. severe, not sufficiently controlled diabetes mellitus) - Patients with unresolved hepatitis B or C infection or known HIV positive infection (mandatory test) - Concomitant or previous malignancies within the last 3 years other than basal cell skin cancer, Prostate cancer in remission with PSA within normal range or in situ uterine cervix cancer |
| **Treatment schedule** | **ARM A (VR-I):**  Induction, cycle length 28 days:  Venetoclax:  Cycle 1: day 22-28 20 mg  Cycle 2: day 1 - 7 50 mg  day 8-14 100 mg  day 15-21 200 mg  day 22-28 400 mg  Cycle 3-6: day 1-28 400 mg  Rituximab:  Cycle 1-6: day 1 375 mg/m^2^ i.v.  Ibrutinib:  Cycle 1-6: day 1-28 560 mg  Maintenance, cycle length 28 days:  Venetoclax:  Cycle 7–30: day 1-28 400 mg  Rituximab:  Cycle 7-30: day 1 of every second cycle 375 mg/m^2^ i.v.  Ibrutinib:  Cycle 7-30: day 1-28 560 mg  **ARM B (BR-I):**  Induction, cycle length 28 days:  Bendamustine:  Cycle 1-6: day 1,2 90 mg/m^2^ i.v.  Rituximab:  Cycle 1-6: day 0 or 1 375 mg/m^2^ i.v.  Ibrutinib:  Cycle 1-6: day 1-28 560 mg  Maintenance, cycle length 28 days:  Rituximab:  Cycle 7-30: day 1 of every second cycle 375 mg/m^2^ i.v.  Ibrutinib:  Cycle 7-30: day 1-28 560 mg |
| **Follow up** | All subjects who enter the trial will continue to be followed every 6 months for disease progression, subsequent treatment, and survival until at least two years after last patient last treatment up to a maximum of 5 years (LPLT). |
| **Trial duration and dates** | First subject in Q1/2023  Last subject out Q3/2028  Duration of recruitment Approximately 3 years  Duration of the trial 66 months (primary endpoint reached for all patients)  Transition to CTR 536/2014 Q1/2023 |
| **Number of subjects** | It is planned to enroll 150 subjects |
| **Randomization** | A stratified central block randomization will be used for allocation of patients to both arms in a 1:1 ratio. Patients will be randomized until the final number is reached in each arm. |
| **Number of sites** | Approx. 40 trial sites are planned to participate in Germany and Italy |
| **Statistical methods** | Sample size estimation:  Allowing for 10% dropouts, the sample size of 75 per group gives 90% power to detect an improvement by 15% to 75% 30-month FFS for each treatment arm when compared to the outcome with R-CHOP+R maintenance (60% 30-month FFS) using one-sided binomial tests with significance level 0.10.  Analysis of Primary Endpoint:  Separately for each of the two trial arms, the frequency and the percentage of failure-free survival among all evaluable patients will be reported. A one-sided lower-bounded 10%-exact confidence interval will be calculated for the estimated failure-free survival probability in each treatment arm. For each treatment arm, a separate exact binomial hypothesis test will be performed using the following one-sided hypotheses:   - Null hypothesis H0: 30-month FFS ≤ 60% - Alternative hypothesis HA: 30-month FFS > 60%   The significance level will be set to 10% accounting for the phase 2 design with limited sample size.  Analysis of Secondary Endpoints:  Secondary efficacy endpoints will be evaluated with descriptive statistical methods separately in each treatment group. Depending on the data type, absolute and relative frequencies, median, IQR, range, or Kaplan-Meier estimates will be calculated. For probabilities estimated by relative frequencies or the Kaplan-Meier method, two-sided 90% confidence intervals will be reported.  Failure-free survival, progression-free survival, and overall survival as time-to-event variables will be exploratively compared between the two treatment arms using Cox regression.  Quality of Life (QoL) will be assessed using mixed-models regression for the *a priori* defined primary QoL domains Global QoL, and secondary QoL domains Physical, condition/fatigue, Symptom burden. The remaining QoL domains will be descriptively analyzed per trial arm and the differences per scale will be checked against a threshold of 10 points (clinical relevance). |

| Visits  Action | **Screening** | | **Cycle 1** | | | | | **Cycle 2** | | | | | **C3** | **Staging**  prior cycle 4 | **Cycle4-6** | **End of induction EoI ^5^** | **Maintenance Visit** every 8 weeks for 24 cycles* | End of Maintenance **EoM** or 24 mo after EoI | **Follow up** Every 6 months after EoM |
| --- | --- | --- | --- | --- | --- | --- | --- | --- | --- | --- | --- | --- | --- | --- | --- | --- | --- | --- | --- |
|  | -28 - 0 | -7 - 0 | D1 | D8 | D15 | D22 | D23 | D1 | D2 | D8 | D15 | D22 | D1 |  | Day 1 |  |  |  |  |
| Demographics | x |  |  |  |  |  |  |  |  |  |  |  |  |  |  |  |  |  |  |
| Informed consent | x |  |  |  |  |  |  |  |  |  |  |  |  |  |  |  |  |  |  |
| Prior/concomitant diseases | x |  |  |  |  |  |  |  |  |  |  |  |  |  |  |  |  |  |  |
| Prior/concomitant medication | x |  | x | x | x | x | x | x | x | x | x | x | x | x | x | x | x | x |  |
| In-/Exclusion criteria check | x |  |  |  |  |  |  |  |  |  |  |  |  |  |  |  |  |  |  |
| Physical examination | x |  | x^1^ |  |  |  |  | x |  |  |  |  | x | x | x | x^1^ | every 6 months | x^1^ | every 12 month |
| ECOG | x |  | x | x | x | x | x | x | x | x | x | x | x | x | x | x | x | x | X |
| Vital signs (inkl. SaO2) |  | x | x | x | x | x | x | x | x | x | x | x | x | x | x | x | x | x | X |
| Height (once), Weight | x |  | x |  |  |  |  | x |  |  |  |  | x |  | x | x | x | x |  |
| ECG |  | x |  |  |  |  |  |  |  |  |  |  |  | if indic. |  | if indic. | if indicated | if indicated | if indicated |
| Echocardiogram (recomm.) | (x) |  |  |  |  |  |  |  |  |  |  |  |  |  |  | (x) |  |  |  |
| PFT (incl DLCO) | x |  |  |  |  |  |  |  |  |  |  |  |  |  |  |  |  |  |  |
| Lab: General | xA | xB |  |  |  |  |  |  |  |  |  |  |  | x |  | x |  |  |  |
| Lab: Small |  |  | x |  | x |  |  |  |  | x | x | x | x |  | x |  | x | x |  |
| Lab: Tumorlysis |  |  |  | x |  | xx(xx hr) | x | xx(xx hr) | x | (xx hr) | (xx hr) | (xx hr) |  |  |  |  |  |  |  |
| Anti-COVID19 immunity | x |  |  |  |  |  |  |  |  |  |  |  |  |  |  | x | Week 53 | x |  |
| HIV, Hep B/C, EBV, CMV |  | x |  |  |  |  |  | HepB^2^ |  |  |  |  |  |  | Hep B^2^ C4, 6 |  | Hep B^2^ |  |  |
| CT Neck/Chest/Abd./ Pelvis | x^3^ |  |  |  |  |  |  |  |  |  |  |  |  | x |  | x | each 6 months | x | 12 mo after EoM |
| PET |  |  |  |  |  |  |  |  |  |  |  |  |  |  |  | x |  |  |  |
| Bone marrow biopsy | x^4^ |  |  |  |  |  |  |  |  |  |  |  |  | x^4^ |  | x^4^ |  | x^4^ |  |
| MRD /Immunrecon | x |  |  |  |  |  |  | - |  |  |  |  |  | x |  | x | every 24 weeks | x | every 12 month |
| Reference pathology | x |  |  |  |  |  |  |  |  |  |  |  |  |  |  |  |  |  |  |
| QoL questionnaires |  | x |  |  |  |  |  |  |  |  |  |  |  | x |  | x | Week 53 | x | once yearly |
| Geriatric Assessment | x |  |  |  |  |  |  |  |  |  |  |  |  |  |  |  |  |  |  |
| Venetoclax / mg daily |  |  |  |  |  | 20 | x | ↑50 | x | ↑100 | ↑200 | ↑400 | 400mg daily until End of Maintenance | | | | |  |  |
| Ibrutinib / mg daily |  |  | 560 mg daily until End of Maintenance | | | | | | | | | | | | | | |  |  |
| Rituximab |  |  | x |  |  |  |  | x |  |  |  |  | x |  | x |  | x |  |  |
| Adverse events |  |  | x | x | x | x | x | x | x | x | x | x | x | x | x | x | x | x |  |
| Subsequent Therapy |  |  |  |  |  |  |  |  |  |  |  |  |  |  |  |  |  | x | x |
| EMCL Registry- ICF (opt) | (x) |  |  |  |  |  |  |  |  |  |  |  |  |  |  |  |  |  |  |

**Trial Schedule Arm A – VR-I Regime**

Trial schedule Arm B – BR-I Regime

| Visits  Action | **Screening** | | **Cycle 1** | | | **Cycle 2-3** | | **Staging**  prior cycle 4 | **Cycle 4-6** | | End of induction EoI^5^ | **Maintenance** every 8 weeks, for 24 cycles* | **End of Maintenance** (EoM) or 24 months after EoI | **Follow up** Every 6 months after EoM |
| --- | --- | --- | --- | --- | --- | --- | --- | --- | --- | --- | --- | --- | --- | --- |
|  | -28 - 0 | -7- 0 | Day 1 | D2 | D15 | D1 | D2 |  | D1 | D2 |  |  |  |  |
| Demographics | x |  |  |  |  |  |  |  |  |  |  |  |  |  |
| Informed consent | x |  |  |  |  |  |  |  |  |  |  |  |  |  |
| Prior/concomitant diseases | x |  |  |  |  |  |  |  |  |  |  |  |  |  |
| Prior/concomitant medication | x |  | x | x | x | x | x | x | x | x | x | x | x |  |
| In-/Exclusion criteria check | x |  |  |  |  |  |  |  |  |  |  |  |  |  |
| Physical examination | x |  | x^1^ |  |  | x |  | x | x |  | x^1^ | every 6 months | x^1^ | every 12 month |
| ECOG | x |  | x | x | x | x | x | x | x | x | x | x | x | x |
| Vital signs (inkl. SaO2) |  | x | x | x | x | x | x | x | x | x | x | x | x | x |
| Height (once), Weight | x |  | x |  |  | x |  |  | x |  | x | x | x |  |
| ECG |  | x |  |  |  |  |  | if indicated |  |  | if indicated | if indicated | if indicated | if indicated |
| Echocardiogram (recomm.) | (x) |  |  |  |  |  |  |  |  |  | (x) |  |  |  |
| PFT (incl DLCO) | x |  |  |  |  |  |  |  |  |  |  |  |  |  |
| Lab: General | xA | xB |  |  |  |  |  | x |  |  | x |  |  |  |
| Lab: Small |  |  | x |  | x | x |  |  | x |  |  | x | x |  |
| Anti-COVID19 immunity | x |  |  |  |  |  |  |  |  |  | x | Week 53 | x |  |
| HIV, Hep B/C, EBV,CMV |  | x |  |  |  | HepB^2^ Cycle2 |  |  | HepB^2^ C4,6 |  |  | HepB^2^ |  |  |
| CT Neck/Chest/Abd/Pelvis | x^3^ |  |  |  |  |  |  | x |  |  | x | every 6 months | x | 12 mo after EoM |
| PET |  |  |  |  |  |  |  |  |  |  | x |  |  |  |
| Bone marrow biopsy | x^4^ |  |  |  |  |  |  | x^4^ |  |  | x^4^ |  | x^4^ |  |
| MRD / immunrecon | x |  |  |  |  |  |  | x |  |  | x | every 24 weeks | x | every 12 month |
| Reference pathology | x |  |  |  |  |  |  |  |  |  |  |  |  |  |
| QoL questionnaires |  | x |  |  |  |  |  | x |  |  | x | Week 53 | x | once yearly |
| Geriatric Assessment | x |  |  |  |  |  |  |  |  |  |  |  |  |  |
| Bendamustine 90mg/m^2^ |  |  | x | x |  | x | x |  | x | x |  |  |  |  |
| Ibrutinib 560 mg |  |  | 560 mg daily until End of Maintanance | | | | | | | | | |  |  |
| Rituximab 375 mg/m^2^ |  |  | x |  |  | x |  |  | x |  |  | x |  |  |
| Adverse events |  |  | x | x | x | x | x | x | x | x | x | x | x |  |
| Subsequent Therapy |  |  |  |  |  |  |  |  |  |  |  |  | x | x |
| EMCL Registry – ICF (optional) | x |  |  |  |  |  |  |  |  |  |  |  |  |  |

**Footnotes**

1 Assessment not necessary, if done within 7 days before

2 Retest of HBV-DNA only if patient was HBcAb positive at screening. Retesting only every second cycle during Induction and Maintenance (C2, 4, 6, 8, 10, 12, and so on)

3 Baseline CT results acceptable within 42 days before first dose

4 If a prior positive bone marrow result exists, no re-biopsy at staging is necessary. Retesting during study is only necessary if bone marrow was infiltrated at screening

5 All patients should perform EoI Visit within 3 weeks (± 7 days) after d1 of the last cycle or before maintenance or any new antineoplastic therapy

6 MRD/Immunreconstitution timepoints: Baseline, Staging prior cycle 4, EoI and during maintenance Z12 D1 ; Z17 D1; Z21 D1; Z26 D1; EoM, FU every 12 months

* First maintenance visits will be performed at day 57 after d1 of last induction cycle

Lab General:

A (within day -28 - 0) GGT, total bilirubin, total protein, TSH, fT3, fT4, BSG, CRP, LDH, immunoglobuline levels (IgG), vitamin D, ferritin, electrophoresis and immunofixation in blood and urine (repeat only if positive at screening)

B (within day -7 - 0): Whole blood count and differential, creatinine/eGFR, uric acid, urea, ASAT, ALAT, sodium, potassium, calcium, phosphate.

Lab Small: Whole blood count and differential, creatinine, uric acid, LDH, ASAT, ALAT, GGT, total bilirubin

Lab Tumorlysis: At Venetoclax ramp-up days multiple TLS labs have to be drawn pre and post dosing, depending on individual TLS risk of patient. Definition of TLS risk isdescribed section 7.2.4

Pre dose only: Whole blood count and differential,

Pre and multiple post dose according to TLS risk: creatinine, uric acid, LDH, sodium, potassium, calcium, phosphate.

**Mandatory timepoints for Lab tumorlysis please see section 7**

**Table of Contents**

[CLINICAL TRIAL PROTOCOL 1](#_Toc107916520)

[1 SIGNATURES 3](#_Toc107916521)

[2 INTRODUCTION 16](#_Toc107916522)

[2.1 Scientific background 16](#_Toc107916523)

[2.2 Trial rationale 17](#_Toc107916524)

[2.3 Treatments and rationale for dose selection 18](#_Toc107916525)

[2.4 Risk-benefit assessment 18](#_Toc107916526)

[3 TRIAL OBJECTIVES 19](#_Toc107916527)

[3.1 Primary objective 19](#_Toc107916528)

[3.2 Secondary objectives 19](#_Toc107916529)

[4 TRIAL DESIGN 19](#_Toc107916530)

[4.1 Trial duration and schedule 19](#_Toc107916531)

[4.2 Number of subjects and trial centers 20](#_Toc107916532)

[4.3 Primary endpoint 20](#_Toc107916533)

[4.4 Secondary endpoints 20](#_Toc107916534)

[4.5 Measures taken to minimize/avoid bias 20](#_Toc107916535)

[4.5.1 Randomisation 20](#_Toc107916536)

[4.5.2 Blinding/Unblinding 21](#_Toc107916537)

[4.6 Selection and withdrawal of subjects 21](#_Toc107916538)

[4.6.1 Recruitment 21](#_Toc107916539)

[4.6.3 Inclusion criteria 21](#_Toc107916540)

[4.6.4 Exclusion criteria 22](#_Toc107916541)

[4.6.5 Individual Withdrawal / Early termination 23](#_Toc107916542)

[4.6.6 Premature closure of the entire clinical trial 24](#_Toc107916543)

[5 TRIAL TREATMENTS 24](#_Toc107916544)

[5.1 Investigational treatments 24](#_Toc107916545)

[5.1.1 General information about Ibrutinib (IMP 1) 24](#_Toc107916546)

[5.1.1.1 Pharmacokinetics 25](#_Toc107916547)

[5.1.1.2 Therapeutic effects 25](#_Toc107916548)

[5.1.1.3 Known side effects 26](#_Toc107916549)

[5.1.2 General information about Venetoclax (IMP 2) 27](#_Toc107916550)

[5.1.2.1 Pharmacokinetics 27](#_Toc107916551)

[5.1.2.2 Therapeutic effects 28](#_Toc107916552)

[5.1.2.3 Known side effects 28](#_Toc107916553)

[5.2 Dosage schedule 29](#_Toc107916554)

[5.2.1 Arm A (VR-I) Dosage and application 29](#_Toc107916555)

[5.2.2 Arm B (BR-I) Dosage and application 31](#_Toc107916556)

[5.3 Dose Modifications 32](#_Toc107916557)

[5.3.1 Bendamustine 32](#_Toc107916558)

[5.3.2 Ibrutinib 33](#_Toc107916559)

[5.3.3 Rituximab 34](#_Toc107916560)

[5.3.4 Venetoclax 34](#_Toc107916561)

[5.4 Overdose instructions 35](#_Toc107916562)

[5.5 Recommended concommitant medications, prophylaxes and precautions 36](#_Toc107916563)

[5.6 Treatment assignment 37](#_Toc107916564)

[5.6.1 Treatment after the end of the trial 37](#_Toc107916565)

[5.7 Packaging and labelling 37](#_Toc107916566)

[5.7.1 Venetoclax 37](#_Toc107916567)

[5.7.2 Ibrutinib 37](#_Toc107916568)

[5.8 Drug Handling 38](#_Toc107916569)

[5.8.1 Return of unused medication 38](#_Toc107916570)

[5.8.2 Safety instructions 38](#_Toc107916571)

[5.8.3 Procedures for monitoring subject compliance 38](#_Toc107916572)

[5.9 Not permitted medication 38](#_Toc107916573)

[6 TRIAL SCHEDULE 40](#_Toc107916574)

[6.1 Screening and Baseline: Days -28 until -1 (Arm A VR-I and Arm B BR-I) 40](#_Toc107916575)

[6.2 Induction Treatment (Cycle length 28 days) 42](#_Toc107916576)

[6.2.1 **Day 1** of every induction cycle (Arm A VR-I and Arm B BR-I) 42](#_Toc107916577)

[6.2.2 **Day 2** Arm A VR-I only cycle 2; Arm B BR-I everey induction cycle 42](#_Toc107916578)

[6.2.3 **Day 8** only Arm A VR-I at induction cycle 1 and 2 43](#_Toc107916579)

[6.2.4 **Day 15** Arm A VR-I induction cycle 1 and 2; Arm B BR-I at induction cycle 1) 43](#_Toc107916580)

[6.2.5 **Day 22** Arm A VR-I induction cycle 1 and 2 44](#_Toc107916581)

[6.2.6 **Day 23** only cycle 1 Arm A VR-I 44](#_Toc107916582)

[6.2.7 **Staging** prior induction cycle 4, Arm A VR-I and Arm B BR-I 45](#_Toc107916583)

[6.2.8 **End of Induction Visit EoI** (Arm A VR-I and Arm B BR-I) 45](#_Toc107916584)

[6.3 Maintenance Treatment 46](#_Toc107916585)

[6.3.1 **Maintenance Visit** every 8 weeks until for 24 cycles(Arm A VR-I and Arm BR-I) 46](#_Toc107916586)

[6.3.2 **End of Maintenance Visit** 24 months after EoI (Arm A VR-I and Arm B BR-I) 46](#_Toc107916587)

[6.4 Follow Up Phase 47](#_Toc107916588)

[6.4.1 **Follow Up Visit** every 6 months +/- 4 weeks (Arm A VR-I and Arm B BR-I) 47](#_Toc107916589)

[7 TRIAL METHODS 47](#_Toc107916590)

[7.1 Assessment of efficacy 47](#_Toc107916591)

[7.2 Other assessments 50](#_Toc107916592)

[7.2.1 Previous and intercurrent illnesses 50](#_Toc107916593)

[7.2.2 Previous and intercurrent medical treatments 50](#_Toc107916594)

[7.2.3 Laboratory parameters and vital signs 50](#_Toc107916595)

[7.2.4 Tumor Lysis Syndrom (TLS) Risk Assessment 50](#_Toc107916596)

[8 SAFETY 50](#_Toc107916597)

[8.1 Definitions 50](#_Toc107916598)

[8.1.1 Documentation of special situations 50](#_Toc107916599)

[8.1.2 Onset and end date of AEs and SAEs 51](#_Toc107916600)

[8.2 Assessment of AEs by investigator (1^st^ assessment) 51](#_Toc107916601)

[8.2.1 Assessment of Intensity/Severity 51](#_Toc107916602)

[8.2.2 Assessment of Seriousness 51](#_Toc107916603)

[8.2.3 Assessment of Causal relation to trial medication/procedures 52](#_Toc107916604)

[8.3 Period of observation 53](#_Toc107916605)

[8.4 Documentation of AEs and Follow-up 53](#_Toc107916606)

[8.5 Immediate reporting of SAEs by investigator 53](#_Toc107916607)

[8.6 Safety evaluation and Reporting by sponsor 54](#_Toc107916608)

[8.7 Immediate Reporting of pregnancy by investigator 54](#_Toc107916609)

[8.8 Emergency procedures 55](#_Toc107916610)

[9 STATISTICS 55](#_Toc107916611)

[9.1 Sample size 55](#_Toc107916612)

[9.2 Analysis populations 55](#_Toc107916613)

[9.3 Efficacy analyses 56](#_Toc107916614)

[9.3.1 Definition and analysis of primary endpoint 56](#_Toc107916615)

[9.3.2 Analysis of secondary endpoints 56](#_Toc107916616)

[9.3.3 Analysis of Subgroups 57](#_Toc107916617)

[9.3.4 Interim analyses 57](#_Toc107916618)

[9.4 Analysis of adverse events 58](#_Toc107916619)

[9.5 Analysis of quality of life 58](#_Toc107916620)

[9.5.1 Instruments 58](#_Toc107916621)

[9.5.1.1 QLQ-C30 58](#_Toc107916622)

[9.5.1.2 QLQ-NHL-HG29 58](#_Toc107916623)

[9.5.2 Data collection and Monitoring of QoL compliance 59](#_Toc107916624)

[9.5.3 QoL schedule 59](#_Toc107916625)

[9.5.4 QoL analysis 60](#_Toc107916626)

[9.5.4.1 Power considerations 60](#_Toc107916627)

[9.5.4.2 Primary analysis 60](#_Toc107916628)

[9.5.4.3 Secondary analysis 60](#_Toc107916629)

[9.5.4.4 Supportive analysis 60](#_Toc107916630)

[9.5.4.5 Exploratory analysis 61](#_Toc107916631)

[9.5.4.6 Missing data 61](#_Toc107916632)

[9.6 Geriatric assessment evaluation 61](#_Toc107916633)

[10 QUALITY CONTROL, QUALITY ASSURANCE AND RISK MANAGEMENT 62](#_Toc107916634)

[10.1 Requirements for Investigator, investigational sites and members of the investigating staff 62](#_Toc107916635)

[10.2 Quality of the source data 62](#_Toc107916636)

[10.3 When a copy is used to replace an original document, the copy has to fulfill the requirements for certified copies.Direct access to source data/documents 63](#_Toc107916637)

[10.4 Monitoring 63](#_Toc107916638)

[10.5 Risk Management 63](#_Toc107916639)

[10.6 Measures to secure compliance 64](#_Toc107916640)

[10.7 Inspection by authorities 64](#_Toc107916641)

[10.8 Audits 64](#_Toc107916642)

[11 DATA MANAGEMENT 64](#_Toc107916643)

[11.1 Responsibilities 64](#_Toc107916644)

[11.2 Data collection 64](#_Toc107916645)

[11.3 Data handling 65](#_Toc107916646)

[11.4 Storage and archiving of data 66](#_Toc107916647)

[11.5 Long Term follow up of Patients of the MCL-Elderly-III-trial in the EMCL-Registry 66](#_Toc107916648)

[12 ETHICAL AND LEGAL ASPECTS 67](#_Toc107916649)

[12.1 Good clinical practice 67](#_Toc107916650)

[12.2 Legal Requirements 67](#_Toc107916651)

[12.3 Subject information and informed consent 67](#_Toc107916652)

[12.4 Confidentiality 68](#_Toc107916653)

[12.5 Responsibilities of the investigator 68](#_Toc107916654)

[12.6 Documentation of serious breaches 68](#_Toc107916655)

[12.7 Approval of trial protocol and amendments 69](#_Toc107916656)

[12.7.1 Submissions 69](#_Toc107916657)

[12.7.2 Substantial Modifications 69](#_Toc107916658)

[12.7.3 Other information to the concerned member states 69](#_Toc107916659)

[12.8 Data monitoring committee (DMC) 69](#_Toc107916660)

[12.9 Insurance 70](#_Toc107916661)

[12.10 Agreements 70](#_Toc107916662)

[12.10.1 Financing of the trial 70](#_Toc107916663)

[12.10.2 Report 70](#_Toc107916664)

[12.10.3 Publication policy 70](#_Toc107916665)

[13 REFERENCES 72](#_Toc107916666)

[14 APPENDICES 75](#_Toc107916667)

[14.1 ANNEX I SAFETY DEFINITIONS 75](#_Toc107916668)

[14.2 ANNEX II Sample List of Cautionary Medications 77](#_Toc107916669)

[14.3 ANNEX III Lugano classification for staging of lymphomas (derived from Ann Arbor staging with Cotswolds modifications) 79](#_Toc107916670)

# INTRODUCTION

## Scientific background

Mantle cell lymphoma (MCL) is a rare but challenging B-cell-lymphoma. Patients are usually elderly, with a median age at diagnosis of approx. 65 years and there is a clear male predominance. Clinically the disease is in general widespread at diagnosis and only few patients present with localized stages. Interestingly, there is a substantial proportion of patients with extranodal manifestation as the gastrointestinal tract. Bone marrow manifestation is frequent and very sensitive methods demonstrate the blood/bone marrow compartment affected in almost every patient (1). The disease is characterized by the *t*(11;14) which leads to overexpression of Cyclin D1, resulting in a growth advantage of the malignant cell. During the disease course, secondary genetic changes frequently occur (e.g. TP53 alterations) and result in an increasingly aggressive behaviour, which finally result in refractoriness to conventional treatments.

A clinical risk score has been established, which is prognostic but does not affect treatment selection. This mantle cell lymphoma international index (MIPI) discriminates 3 risk groups, low, intermediate and high risk with 83% of patients surviving at 5 years in the low risk group, whereas the patients in the high risk group have a median survival of 2.8 years in the validation cohort of patients treated with immunochemotherapy and autologous stem cell transplantation or maintenance (2, 3). The addition of biologic risk factors as p53 mutation/deletion and the expression of Ki67 > 30% identifies specifically high-risk patients. If Ki67 is added to the MIPI (MIPI-C) four discriminative risk groups can be identified, which, especially in the very low and highest risk groups, may be used for treatment stratification in the future (4).

Current treatment concepts are basically selected on patient’s age and performance status. In young, fit patients an intensive first line treatment with a cytarabinoside containing induction, followed by consolidative high dose therapy and Rituximab maintenance is currently the standard of care (5, 6). With this treatment concept, progression free survival has significantly improved over recent years with up to 80% of patients being disease-free 5 years after initiation of treatment in prospective trials (7). For elderly patients a variety of chemoimmunotherapy options exist, e.g. Bendamustine-Rituximab (BR) or Rituximab-Cyclophosphamide-Doxorubicin-Vincristin-Prednisone (R-CHOP) or variants as VR-CAP (Vincristine replacement by Bortezomib) (8-13).Maintenance with Rituximab is regularly applied. With currently available trial results and data from epidemiological registries a median PFS of 3-4 years or even longer is observed for elderly patients after first line treatment.

However, although major progress has been made in first line treatment, in relapse re-exposition to chemotherapy frequently shows disappointing results. Cellular therapies as allogeneic transplantation is potentially curative, but only a minority of patients are eligible due to the known side effects of allo SCT (14-17). Therefore, in the last decade novel targeted agents have drawn major attention. Amongst those, especially BTK inhibitors have shown high efficacy superior to chemotherapy regimens (18, 19). Namely, the first in class drug Ibrutinib represents a new standard of care especially in early relapses after chemotherapy.

While other approved agents have shown some efficacy, they do not achieve similar results as BTK-inhibitors and therefore are currently used in later treatment lines. Very scarce data exist for BTKi-failure patients with agents like Lenalidomide (20), Temsirolimus or Bortezomib (19, 21, 22). Venetoclax, a first in class bcl-2 inhibitor has shown substantial efficacy in this highly challenging situation. The drug has achieved high response rates (75%) in relapsed MCL, but duration of remission after monotherapy seems to be limited, which makes an application in first line desirable (23). Recently, CAR-T-cell-treatment has been introduced for patients failing BTK-inhibitors, however at this time long term data are lacking (24). In order to prolong the efficacy of BTK-based treatment, the combination of Ibrutinib and Venetoclax has been explored in relapsed disease with even molecular remissions in some patients (25). Of note, some patients who stopped treatment experienced durable remission, demonstrating the potential of this combination. In addition, the addition of an anti-CD20 monoclonal antibody – Obinutuzumab - (OASIS) to this combination is feasible and achieved promising results in relapse and first line (26). Results of a randomized trial comparing Ibrutinib to Ibrutinib-Venetoclax (Sympatico) in relapsed MCL are eagerly awaited, as this combination may represent a new standard of care. First available results are encouraging.

Importantly, most patients tolerate these agents very well and long-term treatment is feasible without the side effects of chemotherapy. In relapsed MCL, treatment cessation is frequently not recommended due to the lack of CR-induction- However, whether first line combinations may be given for a limited time is an important, clinically relevant question. If durable responses are achieved, such combinations would challenge current chemotherapy based approaches.

Thus, combinations of novel agents in first line has been initiated, with different approaches applied. On the one hand, Ibrutinib has been added to chemo-immunotherapy (SHINE-trial) in comparison to chemotherapy alone. Recently published results demonstrate a benefit for PFS (27). In a small phase 2 studythe use of Ibrutinib-Rituximab in elderly patients proved the capability to induce excellent and deep remissions in the scenario of treatment naïve patients (28). This is confirmed by a recent report on patients treated with Ibrutinib-Rituximab in a Spanish series of low risk patients.

Therefore, a chemotherapy free combination of Ibrutinib-Venetoclax and an anti-CD20 antibody may effectively challenge the current chemo-immunotherapies, even if those are combined with BTK-inhibitors as potential new standard resulting from the above mentioned SHINE-trial.

At this time, the use of these combinations is restricted to elderly patients not being candidates for high dose therapy, as the results of the ongoing TRIANGLE trial exploring the addition of Ibrutinib to chemotherapy in younger patients are eagerly awaited.

## Trial rationale

Currently, chemotherapy based regimen are the established treatment of choice for MCL patients requiring treatment at all age cohorts. For older patients, R-CHOP or R-Bendamustine followed by R-maintenance represent current standard of care, with BR-Ibrutinib to be expected to become a new standard soon. Especially elderly patients, however, may experience significant chemotherapy related toxicities. In addition, the long-term outcome with immunochemotherapy and maintenance still leaves substantial room for improvement. Therefore, new treatment options with improved tolerability and efficacy would be highly attractive.

The combination of Ibrutinib, Venetoclax and an anti-CD20 antibody has shown promising efficacy and favorable toxicity, therefore challenging the current treatment approach in elderly patients. At this time, we expect the combination or Bendamustine, Rituximab and Ibrutinib to become the optimal available standard for elderly patients with MCL. Similar to BR-I, a chemotherapy free regimen of Venetoclax, Ibrutinib and Rituximab may be superior to the current standard while demonstrating a favorable side effect profile. Besides the antibody treatment, the entire treatment is orally available.

One key challenge of novel agents is the unlimited application of the target agents. As a substantial number of patients experience a reasonable treatment free interval after an induction regimen followed by maintenance and as treatment free time is valuable for patients, we limit treatment at the end of a 2 year maintenance period.

Based on these assumptions we propose to investigate efficacy and safety of the combination of Bendamustine, Ibrutinib and Rituximab for 6 cycles followed by maintenance of Rituximab and Ibrutinib for 2 years and the chemotherapy-free combination of Venetoclax, Ibrutinib and Rituximab for 6 cycles followed by a 24 months of maintenance treatment. Furthermore, the explorative comparison of the two treatment modalities will allow an estimation of relative efficacy as basis for future phase 3 trials.

## Treatments and rationale for dose selection

The combination of BR-I has been evaluated in a variety of trials. Especially the SHINE trial explored this combination. At no time there were signals from the data safety committee indicating increased or unacceptable toxicity in comparison to BR. Therefore, we consider this treatment as safe and established doses will be used in this trial. As evidence emerges that infinitive treatment might not be mandatory in front line treatment, in contrast to the mentioned SHINE trial, duration of maintenance will be shortened to 2 years, which will result in a better safety profile, e.g. in light of the SARS-CoV-2 pandemic.

The Sympatico (NCT03112174) and AIM trials, have evaluated the combination of Ibrutinib and Venetoclax with no reported unforeseen adverse events (25). The combination of Ibrutinib, Venetoclax and Rituximab is considered to be very well tolerated, as suggested from available data. The combination of Ibrutinib and Venetoclax and Obinutuzumab has been explored in the OASIS trial with a favorable side effect profile as compared to conventional treatments (26). As Rituximab is similarly well tolerated as Obinutzumab, the combination investigated in this trial can be considered safe.

## Risk-benefit assessment

The combination of Bendamustine and Rituximab followed by maintenance treatment is an accepted standard of care for patients with MCL, however, this combination has well known side effects. The chemotherapy is associated with cytopenias and increased risk of infections, skin rashes and nausea; Rituximab is associated with especially long-term immunosuppressive side effects. Ibrutinib has a few side effects, including bleeding, infections, atrial fibrillation, gastrointestinal side effects and skin changes.

The experimental treatment contains Ibrutinib and Rituximab as well. Venetoclax toxicity is mainly associated with cytopenias and the risk of tumor lysis syndrome (TLS) early during treatment initiation. Therefore, step-up dosing of Venetoclax will be added after 3 weeks of treatement with Ibrutininb and Rituximab. As substantial tumor reduction can be expected from this prephase, and the risk of TLS will be substantially diminished. In addition, appropriate advice is given to detect early signs of TLS.

Therefore, we assume both regimens to have an acceptable risk-benefit ratio as compared to the immanent risk of active MCL.

The investigators will be informed about any relevant findings including AEs relating to treatment with the investigational medicinal product.

Risk evaluation in relation to the current SARS-CoV-2-Pandemic (“Corona”) situation:

SARS-CoV-2 infections have resulted in a worldwide pandemic. Infections are leading to severe pulmonary complications with the risk of death in a minority, but still meaningful proportion of patients. Patients treated for especially hematologic malignancies experience immunosuppression by their disease *per se*, which in turn is associated with an increased mortality from SARS-CoV-2.

In general, all oncological treatments, which are not urgently required, should be postponed due to current recommendations (e.g. EHA-ESMO / DGHO-guidelines). However, patients included into this protocol have substantial disease related risk for death of MCL, which exceeds the potential risk of infection related complications. Therefore, the risk-benefit-ratio remains positive in favor of conduct of this trial. However, all measures should be undertaken to reduce the risk of infection using protective measures, e.g. patients should have been actively vaccinated in the past, as in line with current recommendations.

**Important precautions**: If a patient is tested positive for SARS-CoV-2 prior to start of study medication treatment ***must not*** be started unless release of signs of infection and proof of negativity with PCR testing.

If a patient becomes positive for SARS-CoV-2 while on treatment, medication has to be stopped ***immediately***. Treatment should be paused unless recovery of clinical signs and negativity in PCR-testing. Restart of treatment should consider the individual risk-benefit for the patient, if a tumor rebound occurs the rules for TLS prevention should be followed for re-inititation of treatment. Upon re-start of a patient after a SARS-CoV-2 infection, regular testing for re-activation of SARS-CoV-2 is recommended. In patients with CR benefits of re-start as compared to surveillance should be carefully weighted.

# TRIAL OBJECTIVES

## Primary objective

The primary objective of the trial is to evaluate the efficacy of a chemo-free regimen of Ibrutinib, Venetoclax and Rituximab and of chemo-immunotherapy in combination with Ibrutinib (Bendamustine, Rituximab, Ibrutinib) in terms of the primary endpoint failure-free survival probability at 30 months in comparison with the results previously obtained with R-CHOP followed by R maintenance.

## Secondary objectives

The secondary objectives of the trial are:

To evaluate the efficacy, safety, tolerability, and quality of life of a chemo-free regimen of Ibrutinib, Venetoclax and Rituximab and of a standard chemo-immunotherapy in combination with Ibrutinib (Bendamustine, Rituximab, Ibrutinib) in terms of secondary endpoints.

Exploratory:

To exploratively compare the efficacy and safety of a chemo-free regimen of Ibrutinib, Venetoclax and Rituximab vs. a standard chemo-immunotherapy in combination with Ibrutinib (Bendamustine, Rituximab, Ibrutinib) in terms of primary and secondary endpoints.

# TRIAL DESIGN

## Trial duration and schedule

The duration of this trial is expected to be 66 months. The subject recruitment is planned to start in Q1/2023 and end in Q3/2028. The actual overall trial duration or subject recruitment period may differ from these time periods. First act of recruitment will be defined as date of first initiation visit for each member state, the trial ends at the last visit of the last patient (including follow up visits). First visit of the first patient is the date of sign of the first ICF for each trial member state.

## Number of subjects and trial centers

It is planned to enroll 150 subjects in the clinical trial, i.e. 75 subjects per treatment group*.* Allowing for 10% dropouts, this sample size gives 90% power to detect an improvement by 15% to 75% 30-month FFS for each treatment arm when compared to the outcome with R-CHOP+R maintenance (60% 30-month FFS) using one-sided binomial tests with significance level 0.10. Recruitment and treatment of subjects is expected to be performed in about 40 trial centers in two countries, namely Germany and Italy.

## Primary endpoint

- The primary endpoint is the failure-free survival status at 30 months from randomization.
- Patients with response (complete or partial remission) to induction treatment and alive without progression at the staging time point 30 months from randomization are classified as achieving failure-free survival, whereas patients with stable disease at end of induction and patients with progression or death from any cause before or in staging at 30 months from randomization are classified as not achieving failure-free survival.

## Secondary endpoints

- Failure-free survival from randomization to failure (stable disease at end of induction or progressive disease) or death from any cause
- Progression-free survival: The time from randomization to first documentation of progression or to death due to any cause, whichever occurs first
- Complete Remission (CR) Rate and Overall Response Rate (ORR: CR or PR) at end of induction treatment
- Best response during 6, 18 and 30 months, time to best response, time to first response
- Overall survival: the time between randomization to date of death due to any cause
- Safety and tolerability as measured as the overall number and grade of adverse events, the percentage of AE’s > Grade 2, the dose adherence, the number of treatment interruptions, the percentage of patients completing treatment as planned
- QoL as measured by EORTC QLQ-C30 and EORTC QLQ-NHL-HG29
- Molecular remission after induction and conversion during maintenance (proposed)
- Immune reconstitution, e.g. persistence of anti-Covid19 immunity
- Overall survival of patients divided according to the geriatric categories and treatment received
- Safety and efficacy in different geriatric categories

## Measures taken to minimize/avoid bias

### Randomisation

After verification of eligibility using a registration checklist patient registration and randomization will be performed via EDC system. Registration is only accepted from authorized investigators and must be performed before start of the treatment. Randomization will ensure equal probability for assignment to any treatment group. Thus, the allocation ratio will be 1:1. Block randomization will be performed stratified according to study groups (countries) and MIPI risk groups (LR/IR vs. HR) at study entry.

Inclusion of patients in the trial will be based on local pathological assessment. In addition, diagnostic material from all study patients must be submitted for central pathologic review.

### Blinding/Unblinding

There is no blinded use of any study drug. Blinding is not considered feasible due to the need for dummy infusions in the VR-I arm and double-dummy control. The primary endpoint FFS is based on quantitative measurements of radiological images without substantial potential for subjective interpretation.

## Selection and withdrawal of subjects

### Recruitment

No subject will be allowed to be enrolled in this trial more than once. Subjects who discontinued prior to the first dose of IMP may be replaced. Subjects withdrawn during study treatment will not be replaced.

- - 1. *Entry procedures*

This is an open-label, randomized study. Eligible patients will be sequentially enrolled in the trial until target enrollment is met. Registration and randomization will be performed via eCRF. The site should maintain a log of all patients who are screened but do not qualify for the study or who do not receive study drug. The reason for disqualification should be noted in the log.

### Inclusion criteria

Subjects meeting all of the following criteria will be considered for admission to the trial:

- Histologically confirmed diagnosis of MCL according to WHO classification
- previously untreated Stage II-IV (Ann Arbor)
- ≥ 60 years and not suitable for autologous SCT
- At least 1 measurable lesion; in case of bone marrow infiltration only, bone marrow aspiration and biopsy is mandatory for all staging evaluations
- ECOG performance status ≤ 2
- The following laboratory values at screening (unless related to MCL):
  - Absolute neutrophil count (ANC) ≥ 1000 cells/µL
  - Platelets ≥ 75.000 cells/µL
  - Transaminases (AST and ALT) ≤ 3 x ULN
  - Total bilirubin ≤ 2 x ULN (unless known Gilbert-Meulengracht-Syndrome)
  - Creatinine ≤ 2 mg/dL or eGFR ≥ 50 mL/min (institutional standard)
  - Written informed consent according to ICH/EU GCP and national regulations. Signed and dated informed consent of the subject must be available before start of any specific trial procedures.
- Sexually active men with female partners of child-bearing potential must agree to use both a highly effective method of birth control (eg, implants, injectables, combined oral contraceptives, some intrauterine devices [IUDs], complete abstinence, or sterilized partner) and a barrier method (eg, condoms, cervical ring, sponge, etc) during the period of therapy and for 90 days after the last dose of study drug. As only patient’s ≥ 60 years are eligible for the trial, no women of child-bearing potential will participate in this trial.
- Ability of subject to understand nature, importance and individual consequences of clinical trial

### Exclusion criteria

Subjects presenting with any of the following criteria will not be included in the trial:

- Major surgery within 4 weeks prior to first dose
- Patients requiring anticoagulation with warfarin or equivalent vitamin K antagonists (e.g. Phenprocoumon)
- Patients requiring double platelet inhibition, e.g due to cardiovascular intervention
- History of stroke or intracranial hemorrhage within 6 months prior to first dose
- Treatment with strong or moderate CYP3A4/5 inhibitors/inducers within 7 days before first dose and during Venetoclax and Ibrutinib intake
- Any life-threatening illness, medical condition, or organ system dysfunction which, in the investigator’s opinion, could compromise the subject’s safety, interfere with the absorption or metabolism of Ibrutinib capsules, or put the study outcomes at undue risk
- Vaccinated with live, attenuated vaccines within 4 weeks prior to first dose
- Known CNS involvement
- Known significant bleeding disorder (e.g. severe von Willebrand disease; hemophilia),
- Clinically significant hypersensitivity (eg, anaphylactic or anaphylactoid reactions to the compound of Ibrutinib itself or to the excipients in its formulation)
- Known anti-murine antibody (HAMA) reactivity or known hypersensitivity to murine antibodies
- Serious concomitant disease interfering with a regular therapy according to the study protocol:
  - Cardiac (Clinically significant cardiovascular disease such as symptomatic arrhythmias, congestive heart failure, or myocardial infarction within 6 months of screening, or any Class 3 (moderate) or Class 4 (severe) cardiac disease as defined by the New York Heart Association Functional Classification or LVEF below LLN)
  - Pulmonary disease with hypoxemia, DLCO ≤ 65% or FEV1 ≤ 65%
  - other relevant and uncontrolled medical situations (e.g. severe, not sufficiently controlled diabetes mellitus)
- For Hepatitis B or C infection or human immunodeficiencyvirus (HIV) infection, the following applies in detail
  - Patients who are seropositive for HIV are not eligible
  - For patients with hepatitis B virus (HBV) those who are seropositive because of hepatitis B virus vaccination are eligible.
  - Patients who are seronegative for HBsAg, but with HBcAb positive serology, will not be excluded from the study and be given Lamivudine (100 mg die) as prophylaxis starting one week before chemotherapy. HBV-DNA should be tested regularly.
  - Patients who are positive for HBsAg are not eligible
  - Patients with hepatitis C virus (HCV) infection are excluded if they have elevated transaminases, alterations in coagulation tests or active virus replication
- Prior organ, bone marrow or peripheral blood stem cell transplantation
- Concomitant or previous malignancies within the last 3 years other than basal cell skin cancer, Prostate cancer in remission with PSA within normal range or in situ uterine cervix cancer
- Any psychological, familiar, or social condition potentially hampering compliance with the study protocol and follow up schedule
- Subjects not able to give consent
  - Subjects without legal capacity who are unable to understand the nature, scope, significance and consequences of this clinical trial
- Participation in another clinical trial within 30 days before first dose in this study, if the patient received an experimental drug
- History of hypersensitivity to the investigational medicinal product or to any drug with similar chemical structure or to any excipient present in the pharmaceutical form of the investigational medicinal product
- Medical or psychological conditions that would jeopardize an adequate and regular completion of the trial

### Individual Withdrawal / Early termination

Subjects can withdraw their consent without giving any reasons at all time during the trial and this should not entail any disadvantages for them. However, the investigator should try to arrange for the performance of a final visit in order to get conclusive findings of investigation.

Treatment may be prematurely stopped for the following reasons:

- At their own request or at request of the legal representative
- At the decision/instigation of the sponsor (e.g. new results become available which question the design of the trial and the respective treatment)
- If continuation of the trial would be detrimental to the subject’s well-being in the opinion of investigator
- In case of non-compliance of participant regarding intake of investigational product and/or keeping visit times and/or due to any other reasons jeopardising data recording

Premature treatment stop should not result in withdrawal of the patient from the trial. Rather, patients in whom treatment was stopped prematurely should be followed for efficacy and safety according to the specifications of this protocol.

In all cases, the reason for premature treatment stop must be recorded in the CRF and in the subject’s medical records. In case of treatment stop of a subject at their own request, as far as possible the reason should be asked for and documented, the subject should be followed up, and all examinations scheduled should be performed and documented.

All ongoing serious adverse events of withdrawn subjects should be followed up until resolution or stabilization of health condition of the subject, but no longer than 30 days after subject’s discontinuation from the trial.

Withdrawn subjects will not be replaced and are accounted for in the prospected dropout rate.

A trial site can be prematurely closed in case of obvious and gross major protocol violations, violations of legal and ethical regulations (GCP), non-compliance of investigator to adhere to protocol rules or upon wish of the individual site.

### Premature closure of the entire clinical trial

For following reasons, the whole trial may be discontinued at the discretion of the sponsor:

- New risks for subjects become known, which change the risk-benefit negatively to an unacceptable level.
- Occurrence of up-to-date unknown adverse events in respect of their nature, severity, and duration or the unexpected increase in the incidence of known adverse events.
- Medical or ethical reasons negatively affecting the continued performance of the trial.
- Difficulties in the recruitment of subjects.
- Withdrawal of marketing or production authorization or import authorization of study drug
- Restrictions of health care authorization
- Absence of sufficient insurance

In this case the concerned member states must be notified by the sponsor.

# TRIAL TREATMENTS

The investigational medicinal products (IMPs) for this trial are Ibrutinib and Venetoclax. Auxiliary medicinal products (AMPs) are Rituximab (both arms) and Bendamustin (arm B). AMPs are obtained by the investigational sites as local commercial product. For Italian sites, Rituximab will be provided by the sponsor. In this cases Rituximab (MabThera) manufractured by Roche with 100 mg or 500 mg concentrate for infusion per Vials will be used.

Please refer to the current information for health professionals and/or the regional labeling information for detailed information about the used AMPs.

## Investigational treatments

### General information about Ibrutinib (IMP 1)

Ibrutinib (IMBRUVICA®) is a first-in-class, potent, orally administered, covalently binding inhibitor of Bruton’s tyrosine kinase (BTK) co-developed by Pharmacyclics LLC and Janssen Research & Development LLC for the treatment of B-cell malignancies. Ibrutinib has been approved in the European Union (EU), for indications including the treatment of patients with MCL who have received at least one prior systemic treatment.

For the most up-to-date and comprehensive nonclinical and clinical information regarding Ibrutinib background, safety, efficacy, in vitro and in vivo preclinical activity, and toxicology of Ibrutinib, always refer to the latest version of the Ibrutinib information for health professionals and/or the applicable regional labeling information.

For details about packaging and storage see 5.7.2.

#### Pharmacokinetics

Following oral administration of Ibrutinib at doses ranging from 420 to 840 mg/day, exposure to Ibrutinib increased proportionally with substantial inter-subject variability. The mean terminal plasma elimination half-life (t1/2) of Ibrutinib ranged from 4 to 13 hours, with a median time to maximum plasma concentration (Tmax) of 2 hours. Despite the doubling in mean systemic exposure when dosed with food, the favorable safety profile of Ibrutinib allows dosing with or without food. Ibrutinib is extensively metabolized primarily by cytochrome P450 (CYP) 3A4. The on-target effects of the main metabolite PCI-45227 are not considered clinically relevant. Steady-state exposure of Ibrutinib and PCI-45227 was less than 2-fold of first dose exposure implying non-clinically relevant accumulation. Less than 1% of Ibrutinib is excreted in the urine. Ibrutinib exposure is not altered in patients with creatinine clearance (CrCl) >30 mL/min. Patients with severe renal impairment or patients on dialysis have not been studied. Following single-dose administration, the AUC of Ibrutinib increased 2.7-, 8.2- and 9.8-fold in subjects with mild (Child-Pugh class A), moderate (Child-Pugh class B), and severe (Child-Pugh class C) hepatic impairment compared to subjects with normal liver function. A higher proportion of Grade 3 or higher adverse reactions were reported in patients with B-cell malignancies (CLL, MCL and WM) with mild hepatic impairment based on NCI organ dysfunction working group (NCI-ODWG) criteria for hepatic dysfunction compared to patients with normal hepatic function.

#### Therapeutic effects

Efficacy results from Study PCYC-04753 and Study PCYC-1104-CA demonstrate that Ibrutinib has activity as a single-agent in treatment of subjects with relapsed or refractory MCL.

Study PCYC-04753: In this Phase 1, multicenter, multicohort, open-label, dose-escalation study, 56 subjects with relapsed or refractory NHL including CLL and Waldenström’s macroglobulinemia were enrolled across 7 dose cohorts (1, 2). Nine of 56 subjects had a diagnosis of MCL and were evaluable for response. Seven of them achieved an objective response by the Revised Response Criteria for Malignant Lymphoma (8), including 3 CRs and 4 partial responses [PRs]; 1 subject had stable disease and 1 subject had progressive disease. All of the subjects responding to treatment achieved response at the time of the first postbaseline response assessment (after 2 cycles of treatment). Of the 3 subjects who achieved a CR, 2 subjects had CR on initial postbaseline assessment, and 1 subject achieved a PR initially and they had a CR after 8 cycles (28-days cycle duration) of therapy. Five subjects who entered a long-term follow-up study have durations of response ranging from 10.5 to 27.5 months.

Study PCYC-1104-CA: This was a multicenter Phase 2 study in 111 subjects with MCL who were relapsed or refractory to their previous treatment (18). Subjects were stratified based on their previous exposure to the chemotherapeutic agent bortezomib. The objectives included studying the efficacy of Ibrutinib given as a continuous fixed dose of 560 mg/day. Overall response rate was the primary end point. 86% of the patients had intermediate- or high-risk MCL. The overall response rate was 68%, complete response was achieved in 21%, partial response in 47% of the patients. Prior treatment with bortezomib had no impact on response. In some patients, treatment with Ibrutinib was associated with a transient increase in peripheral lymphocyte count representing a compartmental shift of cells with the CD19+/CD5+ phenotype from nodal tissues to peripheral blood.

Study MCL2001: In Study MCL2001, a Phase 2 study of Ibrutinib in subjects with MCL, the IRC-assessed ORR was 62.7% (20.9% CR+ 41.8% PR) for the response-evaluable population (n=110). With an estimated median time of efficacy follow-up of 14.5 months, the estimated median DOR was 14.9 months (95% CI: 12.4, not estimable). Median PFS by IRC assessment was 10.5 months, and median OS was not reached. The RAY-trial, an international, randomized phase III trial comparing Ibrutinib to Temsirolimus proved the superiority of BTK on the one hand and on the other hand confirmed the efficacy of Ibrutinib in patients with relapsed MCL (19). The most recent long term follow up of patients treated within the various trials confirmed previous observations (29).

#### Known side effects

Bleeding-related Events: There have been reports of hemorrhagic events in subjects treated with Ibrutinib, both with and without thrombocytopenia. These include minor hemorrhagic events such as contusion, epistaxis, and petechiae; and major hemorrhagic events, rare fatal, including gastrointestinal bleeding, subdural intracranial hemorrhage, and hematuria. Subjects with congenital bleeding diathesis have not been studied. In an in vitro platelet function study, inhibitory effects of Ibrutinib on collagen‑induced platelet aggregation were observed. The use of either anticoagulant or antiplatelet agents concomitantly with Ibrutinib increases the risk of major bleeding. A higher risk for major bleeding was observed with anticoagulant than with antiplatelet agents. Subjects with congenital bleeding diathesis have not been studied.

Leukostasis: There were isolated cases of leukostasis reported in subjects treated with Ibrutinib. A high number of circulating white blood cells (>400.000/μL) may confer increased risk.

Infections: Infections (including sepsis, bacterial, viral, or fungal infections) were observed in subjects treated with Ibrutinib. Some of these reported infections have been associated with hospitalization and death. Although causality has not been established, cases of progressive multifocal leukoencephalopathy (PML) have occurred in subjects treated with Ibrutinib. Subjects should be monitored for symptoms (fever, chills, weakness, confusion), and appropriate therapy should be instituted as indicated. It can be expected that patients under treatment have a higher risk of SARS-CoV-2 infection due to impairment or loss of vaccination response.

Cytopenias: Treatment-emergent Grade 3 or 4 cytopenias (neutropenia, thrombocytopenia, and anemia) were reported in subjects treated with Ibrutinib.

Interstitial Lung Disease (ILD): Cases of interstitial lung disease (ILD) have been reported in subjects treated with Ibrutinib.

Cardiac Arrhythmias: Atrial fibrillation, atrial flutter, and cases of ventricular tachyarrhythmia including some fatal events, have been reported in subjects treated with Ibrutinib, particularly in subjects with cardiac risk factors, hypertension, acute infections, and a previous history of cardiac arrhythmia.

Tumor Lysis Syndrome: Tumor lysis syndrome has occasionally been reported with Ibrutinib therapy. Subjects at risk of tumor lysis syndrome are those with high tumor burden prior to treatment.

Non-melanoma skin cancer and other secondary cancers: Non-melanoma skin cancers have occurred in subjects treated with Ibrutinib.

Lymphocytosis: Upon initiation of single agent treatment with Ibrutinib, a reversible increase in lymphocyte counts (i.e., ≥50% increase from baseline and an absolute count >5,000/μL), often associated with reduction of lymphadenopathy, has been observed in most subjects (66%) with CLL/small lymphocytic lymphoma (SLL). This effect has also been observed in some subjects (35%) with MCL treated with Ibrutinib. This observed lymphocytosis is a pharmacodynamic effect and should not be considered progressive disease in the absence of other clinical findings. In both disease types, lymphocytosis typically occurs during the first month of Ibrutinib therapy and typically resolves within a median of 8 weeks in subjects with MCL and 14 weeks in subjects with CLL/SLL (range 0.1 to 104 weeks).

Cerebrovascular Accidents: Although causality has not been established, cases of cerebrovascular accident, transient ischemic attack, and ischemic stroke including fatalities have been reported with the use of Ibrutinib in the post-marketing setting, with and without concomitant atrial fibrillation and/or hypertension.

Diarrhea: Diarrhea is the most frequently reported non-hematologic AE with Ibrutinib monotherapy and combination therapy. Other frequently reported gastrointestinal events include nausea, vomiting, and constipation. These events are rarely severe and are generally managed with supportive therapies including antidiarrheals and antiemetics.

Rash and skin changes: Rash has been commonly reported in subjects treated with either single agent Ibrutinib or in combination with chemotherapy. Most rashes were mild to moderate in severity. Isolated cases of severe cutaneous adverse reactions (SCARs) including Stevens-Johnson syndrome (SJS) have been reported in subjects treated with Ibrutinib.

Hypertension: Hypertension has been commonly reported in subjects treated with Ibrutinib.

### General information about Venetoclax (IMP 2)

Venetoclax (VENCLYXTO®) is a potent, orally administered inhibitor of B-cell lymphoma 2 (BCL-2) co-developed by AbbVie Inc and Genentech Inc for the treatment of B-cell malignancies and acute myelogenous leukemia (AML). In the EU, Venetoclax in combination with Rituximab is indicated for the treatment of adult patients with CLL who have received at least 1 prior therapy and for a subgroup of patients with AML. Monotherapy is indicated for the treatment of CLL in the presence of 17p deletion or TP53 mutation in adult patients who are unsuitable for or have failed a B-cell receptor pathway inhibitor, or in the absence of 17p del or TP53 mutation in adult patients who have failed both chemoimmunotherapy and a B-cell receptor pathway inhibitor.

Please refer also to the latest version of the Venetoclax information for health professionals and/or the applicable regional labeling information.

For details about packaging and storage see 5.7.1

#### Pharmacokinetics

Following multiple oral administrations under fed conditions, maximum plasma concentration of Venetoclax was reached 5-8 hours after dose. Venetoclax steady state AUC increased proportionally over the dose range of 150-800 mg. Food can increase Venetoclax exposure (3.4-fold with a low-fat meal and 5.1- to 5.3-fold with a high-fat meal). Venetoclax should be administered with a meal. The population estimate for the terminal elimination half-life of Venetoclax was approximately 26 hours. In vitro studies demonstrated that Venetoclax is predominantly metabolized by CYP3A4/5. Less than 0.1% of Venetoclax is excreted renally. Venetoclax exposures in subjects with mild or moderate renal impairment are similar to those with normal renal function. The PK of Venetoclax has not been studied in subjects with severe renal impairment (CrCl <30 mL/min) or subjects on dialysis. Venetoclax exposures are similar in subjects with mild and moderate hepatic impairment and normal hepatic function based on the NCI Organ Dysfunction Working Group criteria. Mild hepatic impairment was defined as normal total bilirubin and aspartate transaminase (AST) > upper limit of normal (ULN) or total bilirubin >1.0 to 1.5 times ULN, moderate hepatic impairment as total bilirubin >1.5 to 3.0 times ULN, and severe hepatic impairment as total bilirubin >3.0 times ULN. The PK of Venetoclax has not been studied in subjects with severe hepatic impairment. For the most comprehensive information regarding PK and product metabolism, please refer to the current Venetoclax information for health professionals.

#### Therapeutic effects

Doses administered in Venetoclax clinical studies have ranged from 20 mg to 1200 mg. As of 28 November 2018, a total of 576 NHL subjects treated with Venetoclax in the oncology clinical program had open-label or unblinded safety data available, including 136 subjects who received Venetoclax monotherapy, and 440 subjects who received Venetoclax in combination with other agents including Rituximab, BR, R-CHOP, or G-CHOP. Fifty additional subjects received BR in Study BO29337. Of the 576 NHL subjects treated with Venetoclax, 570 subjects are included in the pooled analyses across all studies, and data from the remaining 6 subjects who crossed over to Venetoclax treatment in Study BO29337 are excluded from the pooled analyses. Overall for NHL, when treated with Venetoclax as a single agent or in combination with other therapies, the most common adverse events were nausea, neutropenia, and diarrhea. Approximately three-fourths of subjects experienced ≥ Grade 3 adverse events, and the most common events were neutropenia, thrombocytopenia, and anaemia. The most common SAEs were febrile neutropenia, neutropenia, and pneumonia. Of the fatal events in the NHL program, the majority were adverse events of malignant neoplasm progression. Findings from the analysis based on the exposure-adjusted incidence rates were consistent with the findings from the analysis based on the subject incidence rates. Many of the adverse events reported in the current NHL studies are consistent with underlying disease or concomitant medical conditions, as well as other combination agents used to treat NHL patients. Safety in combination agents appears to be consistent with that observed in monotherapy trials and combination backbone regimen.

The incidence of TLS in NHL studies is low with reports of 2 cases (1.9%) of laboratory TLS in monotherapy Study M12-175, 4 cases of laboratory TLS (2.5%) in combination Study BO29337 (1 subject Venetoclax + R, 3 subjects Venetoclax + BR), and 4 cases of laboratory TLS (1.5%) in combination Study GO27878 (Venetoclax + R-CHOP or G-CHOP). All cases of TLS resolved, and none led to discontinuation of study drug. No cases of clinical TLS were reported. Neutropenia has a similar frequency in the NHL clinical program as in CLL with higher frequency in NHL combination studies. Serious adverse events of neutropenia and febrile neutropenia, albeit in small numbers, occurred in higher frequency in combination studies. Infections, including serious, were observed in the NHL clinical program, with similar incidence in monotherapy and combination studies.

#### Known side effects

Tumor Lysis Syndrome: Tumor lysis syndrome (TLS) is an important identified risk for Venetoclax in oncology studies, especially in CLL and MCL. As a result of on-target effects, the potential for TLS was identified early in the program. The risk is increased during the first 5 weeks of ramp-up period. A low starting dose followed by gradual dose ramp-up allows for the tumor size to be gradually reduced and has been effective in reducing the risk of TLS. Therefore, Venetoclax should be initiated with the 20 mg dose and gradually ramp-up/titrate up to 400 mg target dose over 5 weeks.

Neutropenia: Neutropenia is an important identified risk for Venetoclax. Clinical data from the oncology studies suggest that the neutropenia adverse events are observed among subjects who receive Venetoclax as a single agent or in combination with other therapeutic agents, with slightly higher frequency observed in some combination studies. Serious adverse events of neutropenia or neutropenia events that lead to discontinuations are few across the entire Venetoclax oncology program. Neutropenia management guidelines are provided in the protocol (see 6.1.4). Granulocyte colony stimulating factors can be used for supportive measures, however the guidance for their use in non- CLL indications is per routine local oncology practice.

Serious Infections: Serious infection is an important identified risk for Venetoclax. Infections have been reported in the oncology clinical studies; however, these events are confounded by the underlying disease, comorbidities, and other immunosuppressive medications. To date, no clear association has been noted between serious infectious events and neutropenia. The types of infectious events observed generally have been consistent with those anticipated in the elderly population of heavily pretreated subjects with hematologic malignancies and are similar across all indications. Infections are closely monitored in Venetoclax program across all indications. Anti-infective prophylaxis is optional according to institutional standards of care.

## Dosage schedule

### *Arm A (VR-I) Dosage and application*

**ARM A (VR-I)**

**Induction, cycle length 28 days:**

Rituximab:

Cycle 1-6: day 1 375 mg/m^2^ i.v.

Ibrutinib:

Cycle 1-6: day 1-28 560 mg

Venetoclax:

Cycle 1: day 22-28 20 mg

Cycle 2: day 1 - 7 50 mg

day 8 - 14 100 mg

day 15 - 21 200 mg

day 22 - 28 400 mg

Cycle 3-6: day 1 - 28 400 mg

**Maintenance, cycle length 28 days:**

Rituximab:

Cycle 7-30: day 1 of every second cycle 375 mg/m^2^ i.v.

The first dose will be given on day 1 in cycle 8, the last in cycle 30. For organizational purposes in maintenance application can be shifted +/- 2 weeks. The next dose however should follow the initial schedule. Skipped doses will not be replaced.

Ibrutinib:

Cycle 7-30: day 1-28 560 mg

Unless there is not clear evidence of disease progression treatment wil continue from induction without pausing for end of induction evaluation. Skipped doses will not be replaced.

Venetoclax:

Cycle 7-30: day 1-28 400 mg

Unless there is not clear evidence of disease progression treatment wil continue from induction without pausing for end of induction evaluation. Skipped doses will not be replaced.

**Application**

The oral intake of Ibrutinib is continued during the whole dose escalation period by the patients at home.

**Ibrutinib**

- Ibrutinib 560 mg (4 x 140mg capsules) is administered orally once daily. The Ibrutinib capsules are to be taken with Venetoclax tablets at approximately the same time each day with a meal and water. The use of strong CYP3A inhibitors/inducers, and grapefruit and Seville oranges should be avoided for the duration of the study. If a dose is not taken at the scheduled time, it can be taken as soon as possible on the same day with a return to the normal schedule the following day. The subject should not take extra capsules to make up the missed dose. Ibrutinib will be dispensed to subjects at each dispensing visit. Study drug may not be shipped to the subject and may not be dispensed to anyone other than the subject.

**Venetoclax**

Management:

- *Hydration***:** Consider adequate hydration (1.5-2 L) 48 hours prior to initiating therapy with Venetoclax and throughout the ramp-up period, especially on the first day of each ramp-up dose. Administer intravenous (IV) fluids as indicated based on overall risk of TLS or for those who cannot maintain adequate oral hydration.
- *Anti-hyperuricemic agents***:** Consider administer allopurinol 72 hours prior to initiation of Venetoclax; consider continuing through the ramp-up period. Rasburicase is recommended for subjects at high risk, especially those with high tumor burden.

Prophylaxis and Management of Tumor Lysis Syndrome:

- Venetoclax can cause rapid reduction in tumor and thus poses a risk for TLS in the ramp-up period. Changes in electrolytes consistent with TLS that require prompt management can occur as early as 6-8 hours following the first dose of Venetoclax and at each dose increase. The risk of TLS is a continuum based on multiple factors, including comorbidities. Subjects with high tumor burden are at a greater risk of TLS when initiating Venetoclax. Reduced renal function further increases the risk. The risk may decrease as tumor burden decreases with Venetoclax treatment.
- Tumor burden assessments, including radiographic evaluation (eg, CT scan) should be performed at screening as well as blood chemistry assessments (creatinine, uric acid, potassium, sodium, phosphate and calcium) in all subjects. Pre-existing abnormalities should be corrected prior to initiation of treatment with venetoclax. The prophylaxis measures listed in the treatment schedule and below should be followed and more intensive measures (including hospitalization) should be employed as overall risk increases.

Hospitalization:

- Based on investigator assessment, at the time of Venetoclax initiation, subjects with high tumor burden (at least one lesion >10 cm; or at least one lesion >5 cm and circulating lymphocytes >25,000 cells/mm3) and/or eGFR <60 mL/min, are at greater risk of TLS and hospitalization is highly recommended the first 24-48 hours of treatment at the 20 mg and the 50 mg ramp-up doses of Venetoclax for more intensive prophylaxis and monitoring. Consider hospitalization for subsequent ramp-up doses based on reassessment of risk (also section 7.2).
- Venetoclax tablets should be taken orally once daily with a meal and water at approximately the same time each day with Ibrutinib capsules. Venetoclax tablets should be swallowed whole and not chewed, crushed, or broken prior to swallowing. If the subject misses a dose of Venetoclax within 8 hours of the time it is usually taken, the subject should take the missed dose as soon as possible on the same day and resume the normal daily dosing schedule. If a subject misses a Venetoclax dose by more than 8 hours, the subject should not take the missed dose and resume the usual dosing schedule the following day. The use of strong CYP3A inhibitors/inducers, and grapefruit and Seville oranges should be avoided for the duration of the study. Subjects should strictly adhere to the ramp-up schedule. In cases of vomiting after taking Venetoclax, no additional dose (tablets) should be taken that day. The next dose should be taken at the usual time the following day.

**Rituximab**

- *Rituximab* is applied with routine clinical precautions as scheduled in the protocol. Especially during the first infusions all measures should be undertaking to supervise for infusion related symptoms. Institutional standards should be followed.

### *Arm B (BR-I) Dosage and application*

**ARM B (BR-I)**

**Induction, cycle length 28 days:**

Bendamustine:

Cycle 1-6: day 1, 2 90 mg/m^2^ i.v.

Rituximab:

Cycle 1-6: day 1 375 mg/m^2^ i.v.

Ibrutinib:

Cycle 1-6: day 1-28 560 mg

**Maintenance, cycle length 28 days:**

Rituximab:

Cycle 7-30: day 1 of every second cycle 375 mg/m^2^ i.v.

The first dose will be given on day 1 in cycle 8, the last in cycle 30. For organizational purposes in maintenance application can be shifted +/- 2 weeks. The next dose however should follow the initial schedule. Skipped doses will not be replaced.

Ibrutinib:

Cycle 7-30: day 1-28 560 mg

Unless there is not clear evidence of disease progression treatment wil continue from induction without pausing for end of induction evaluation. Skipped doses will not be replaced.

Application

For Ibrutinib and Rituximab see 6.2.1

**Bendamustine**

- Commercially available preparations of Bendamustine will be used in this trial. The drug should be handled as described in the summary of product characteristics and according to institutional standards, e.g. with an infusion duration of 30 minutes.

**Please note:** Due to the risk of toxic skin reactions (e.g. Stevens-Johnson syndrome/toxic epidermal necrolysis), concomitant administration of **allopurinol should be avoided**. If allopurinol is indispensable, it should be stopped ≤24-48 hrs before and reinitiated ≥24-48 hrs after administration of Bendamustine.

## Dose Modifications

Note: as there might be overlap between the toxicities of Bendamustine, Ibrutinib and Venetoclax dose reduction recommendations may apply to all drugs at the same time. However, in clinical routine, dose reductions are frequently performed stepwise. If, in the opinion of the investigator, a stepwise approach could be chosen, this can be followed. Appropriate documentation for the individual strategy should be made, including clarification why which drug has been reduced first. In case of combined toxicities, second dose reduction should be made for the alternate drug. The same applies for dose-re-escalations, however, this needs to be discussed with the coordinating investigator.

In case of to hematological toxicities in arm B (BR-I Regime) first drug to be reduced should be bendamustine.

### Bendamustine

Dosages may be adjusted in case of significant changes in body weight compared to baseline (≥ 10%) leading to changes in BSA. Dose reduction will be calculated according to the doses of Bendamustine given in the previous cycle. Dose reduction should be omitted if cytopenia is associated with bone marrow involvement of the lymphoma. The following dose reductions should apply in case of hematological toxicity.

Table 4: Dose reduction schedule

| **ANC/µl on d 1** | **Thrombocytes/µl on d 1** | **Bendamustine** | **Rituximab** |
| --- | --- | --- | --- |
| >1.000/µl (Grade 2) | >75.000/µl (Grade 1) | 100% | 100% |
| 500 – 1000/µl (Grade 3) | 50.000-75.000/µl (Grade 2) | 75% | 100% |
| < 500/µl (Grade 4) | < 50.000/µl (Grade 3) | 50% | 100% |

There is no re-escalation during subsequent cycles. In patients with already reduced doses, the next reductions step applies.

### Ibrutinib

Ibrutinib-treatment should be interrupted for any new onset or worsening Grade 2 cardiac failure, Grade ≥ 3 non hematological toxicities, Grade 3 or greater neutropenia with infection or fever, or Grade 4 hematological toxicities.

Once the symptoms of the toxicity have resolved to Grade 1 or baseline (recovery), resume Ibrutinib treatment at the recommended dose as per the table below.

Study drug may be interrupted for a maximum of 28 consecutive days for drug-related toxicity. In case of reason justifying a later re-start, this needs to be discussed with the coodinating investigator of the trial.

Table 1: Recommended dose modifications for events of cardiac failure or cardiac arrhythmias

| **Event** | **Toxicity occurrence** | **Dose modification after recovery** |
| --- | --- | --- |
| Grade 2 cardiac filure | First | Restart at 420 mg daily |
|  | Second | Restart at 280 mg daily |
|  | Third | Discontinue study drug |
| Grade 3 cardiac arrhythmias | First | Restart at 420 mg daily* |
|  | Second | Discontinue study drug |
| Grade 3 or 4 cardiac failure  Grade 4 cardiac arrhythmias | First | Discontinue study drug |

*Evaluate the benefit-risk before resuming treatment.

Table 1: Recommended dose modifications for non-cardiac events

| **Events** | **Toxicity occurrence** | **Dose modification after recovery** |
| --- | --- | --- |
| Grade 3 or 4 non-hematological toxicity  Grade 3 or 4 neutropenia with infection or fever  Grade 4 hematological toxicity | First* | Restart at 560 mg daily |
|  | Second | Restart at 420 mg daily |
|  | Third | Restart at 280 mg daily |
|  | Fourth | Discontinue study drug |

*When resuming treatment, restart at the same or lower dose based on benefit-risk evaluation. If the toxicity reoccurs, reduce daily dose by 140 mg.

If the dose is reduced, re-escalation is not foreseen, if not discussed and agreed by the coordinating investigator dose may be re-escalated after 2 months of a dose reduction.

No dose escalation of study drug (more than 4 capsules/day [i.e. above 560 mg]) is allowed in this study.

If an Ibrutinib dose is not taken at the scheduled time, it can be taken as soon as possible on the same day with a return to the normal schedule of the following day. The subject should not take extra capsules to make up the missed dose. Doses that were missed for any reason will not be rescheduled.

### Rituximab

There will be no dose reductions of Rituximab. In case of cycle delay due to Ibrutinib induced toxicity, immunochemotherapy of the next cycle will also be postponed until AE has resolved and recycling is allowed. In case of hold of the entire regimen, missed doses of Rituximab should only be applied if the original schedule can be maintained. If Ibrutinib and/or Venetoclax are terminated prematurely during maintenance treatment, Rituximab may be continued as scheduled. During maintenance treatment skipped doses will not be replaced.

### Venetoclax

Venetoclax dosing interruption and/or dose reduction may be required. See following Table for dose modifications for hematologic and other toxicities related to Venetoclax.

Table 2: Dose modifications (Venetoclax)

| **Event** | **Occurrence** | **Action** |
| --- | --- | --- |
| Tumor Lysis Syndrome | | |
| Blood chemistry changes or symptoms suggestive of TLS | Any | Withhold the next days’ dose. If resolved within 24-48 hours of last dose, resume at the same dose. |
|  |  | For any blood chemistry changes defining TLS requiring more than 48 hours to resolve, resume at a reduced dose (see table 3). |
|  |  | For any events of clinical TLS, resume at a reduced dose following resolution (see table 3). |
| Non-Hematologic Toxicities | | |
| Grade 3 or 4 non-hematologic toxicities | First | Interrupt Venetoclax  Once the toxicity has resolved to Grade 1 or baseline level, Venetoclax therapy may be resumed at the same dose. No dose modification is required. |
|  | Second and subsequent occurences | Interrupt Venetoclax  Follow dose reduction guidelines in table 3 when resuming treatment with Venetoclax after resolution. A larger dose reduction may occur at the discretion of the investigator. |
| Hematologic Toxicities | | |
| Grade 3 or 4 neutropenia with infection or fever; or Grade 4 hematologic toxicities (except lymphopenia) | First | Interrupt Venetoclax  To reduce the infection risks associated with neutropenia, granulocyte-colony stimulating factor (G-CSF) may be administered with Venetoclax if clinically indicated. Once the toxicity has resolved to Grade 1 or baseline level, Venetoclax may be resumed at the same dose. |
|  | Second and subsequent occurrences | Interrupt Venetoclax  Consider using G-CSF as clinically indicated. Follow dose reduction guidelines in table 3 when resuming Venetoclax after resolution. Additional dose reductions may occur at the discretion of the investigator. |

Table 3. Dose Modification for Toxicity during Venetoclax Treatment

| **Dose at Interruption** | **Restart Dose** |
| --- | --- |
| 400 mg | 300 mg |
| 300 mg | 200 mg |
| 200 mg | 100 mg |
| 100 mg | 50 mg |

If the dose of Venetoclax is reduced, at the investigator’s discretion, the dose of Venetoclax may be re-escalated after 2 months of a dose reduction in the absence of a recurrence of the toxicity that led to the reduction, and after discussion with the coordinating investigator.

Consider discontinuing Venetoclax for subjects who require dose reductions to less than 100 mg for more than 2 weeks.

## Overdose instructions

There is no specific experience in the management of Ibrutinib overdose in patients. No MTD was reached in the Phase 1 study in which subjects received up to 12.5 mg/kg/day (1400 mg/day). Healthy subjects were exposed up to a single dose of 1680 mg. One healthy subject experienced reversible Grade 4 hepatic enzyme increases (AST and ALT) after a dose of 1680 mg. Subjects who ingested more than the recommended dosage should be closely monitored and given appropriate supportive treatment.

There is no specific experience in the management of Venetoclax overdose in patients. There is also no specific antidote for Venetoclax. For subjects who experience overdose, closely monitor and provide appropriate supportive treatment; during the ramp-up period, interrupt Venetoclax and monitor carefully for signs and symptoms of TLS along with other toxicities. Based on the large volume of distribution and extensive protein binding of Venetocax, dialysis is unlikely to result in significant removal of Venetoclax.

There is no overdose instruction for Rituximab. No formal MTD has been defined and dosis up to 2g/m^2^ have been used in the past.

In case of Bendamustine overdose, there is no specific measure to be initiated. However, patients need to be followed thouroughly for hematotoxicity.

## Recommended concomitant medications, prophylaxes and precautions

Infectious prophylaxis: Prophylaxis of pneumocystis jirovecii-pneumonia is recommended, e.g. with Cotrimoxazole (Trimethoprim/ Sulfamethoxazole 160/800mg, either 1 tablet 3x/week [Mo/Wed/Fr, 1-0-0] or 2 tablets 2x/week [Mo/Thu, 2-0-0] or according to standard institutional practice). Antiviral and antifungal prophylaxis may be given at the discretion of the investigator. Particularly in patients with history of recurrent infections, e.g. Herpes simplex virus, prophylaxis with Aciclovir is recommended. However, caution should be made regarding possible drug-drug-interactions with Venetoclax and Ibrutinib.

Supportive medications: Standard supportive care therapies, such as antiemetics should be administered according to the institutional practice and to the investigator’s discretion.

Closely follow patients for secondary primary malignancies, with a focus of non-melanoma skin cancer.

Screen for side effects within the cardiovascular system with the investigations included in the protocol (RR-assessment, ECG, Echo). Adopt e.g. antihypertensive medication.

Ibrutinib:

- Interstitial Lung Disease (ILD):

Monitor subjects for pulmonary symptoms indicative of ILD. If symptoms develop, interrupt Ibrutinib and manage ILD appropriately. If symptoms persist, consider the risks and benefits of Ibrutinib treatment and follow the protocol dose modification guidelines as needed.

- Cardiac Arrhythmias:

Periodically monitor subjects clinically for cardiac arrhythmia. Subjects who develop arrhythmic symptoms (e.g., palpitations, lightheadedness, syncope, chest discomfort or new onset of dyspnea) should be evaluated clinically, and if indicated, have an ECG performed. For cardiac arrhythmias which persist, consider the risks and benefits of Ibrutinib treatment and follow the protocol dose modification guidelines.

- Tumor Lysis Syndrome:

Monitor subjects closely for TLS and take appropriate precautions (see for precautions according to venotoclax and follow accordingly).

- Non-melanoma skin cancer and other secondary cancers:

Monitor subjects for the appearance of non-melanoma skin cancer, as well as other secondary neoplasms.

- Diarrhea:

Subjects should be monitored carefully for gastrointestinal AEs and cautioned to maintain fluid intake to avoid dehydration. Medical evaluation should be made to rule out other etiologies such as Clostridium difficile or other infectious agents. Should symptoms be severe or prolonged follow the protocol dose modification guidelines.

- Rash and skin changes:

Subjects should be closely monitored for signs and symptoms suggestive of SCAR including SJS. Subjects receiving Ibrutinib should be observed closely for rashes and treated symptomatically, including interruption of the suspected agent as appropriate. In addition, hypersensitivity-related events including erythema, urticaria, and angioedema have been reported. Careful skin care is recommended.

- Hypertension:

Monitor subjects for new onset of hypertension or hypertension that is not adequately controlled after starting Ibrutinib. Adjust existing anti-hypertensive medications and/or initiate anti-hypertensive treatment as appropriate.

## Treatment assignment

Patients enrolled in this trial will receive either a regime consisting of Venetoclax, Rituximab and Ibrutinib (Arm A) or, Bedamustin, Rituxumab and Ibrutinib (Arm B). Subjects will be randomized in a 1:1 ratio to Arm A or Arm B.

Randomization will be done online with an EDC System.

Subjects withdrawn from the trial retain their identification codes (e.g. randomisation number). New subjects will always receive a new identification code.

### Treatment after the end of the trial

Any choice of subsequent treatment is up to the decision of the treating physician. Type/regimen used and response to subsequent treatment should be reported in the eCRF. Patients should be offered to take part in the registry of the European Mantle Cell Lymphoma network, in which subsequent treatment lines can be registered for better understanding of treatment, response and survival patterns.

## Packaging and labelling

IMPs will be labeled according to § 5 GCP regulation. Anticipating transition to EU Regulation 536/2014, Eudra-CT and EU CT number will be named on the label.

### Venetoclax

Venetoclax is manufactured by AbbVie Inc. and will be supplied as oral tablets of 100 mg, 50 mg and 10 mg. 10 mg tablets are packed in bottles containing 16 tablets each. 50 mg tablets are packed in bottles containing 16 tablets each. 100 mg tablets are packed in bottles containing 32 or 120 tablets each. Venetoclax drug must be stored at 2°C − 25°C at site.

Venetoclax tablets will be packaged in high-density polyethylene plastic bottles to accommodate the study design. Each bottle will be labeled (either single panel or booklet) as per individual country requirements. Label must remain affixed to the supplies.

The investigational product is for investigational use only and is to be used only within the context of this study. The study drug supplied for this study must be maintained under adequate security and stored under the conditions specified on the label until dispensed for patient use or returned to the Sponsor.

### Ibrutinib

Ibrutinib is manufactured by AbbVie and marketed in the EU by Janssen, and will be supplied as capsules for oral intake of 140 mg. Tablets are packed in bottles containing 120 capsules each. Ibrutinib drug must be stored at 15-30°C at the sites. Distribution vendor Clinigen CSM will package and distribute Ibrutinib to participating sites. The investigational product is for investigational use only and is to be used only within the context of this study. The study drug supplied for this study must be maintained under adequate security and stored under the conditions specified on the label until dispensed for patient use or returned to the Sponsor.

## Drug Handling

The investigator will take inventory and acknowledges the receipt of all shipments of the trial medication.

All trial medication must be kept in a locked area with access restricted to designated trial staff. The trial medication must be stored dry and in accordance with manufacturer’s instructions. The investigator will also keep accurate records of the quantities of trial medication dispensed, used, and returned by each subject on the drug accountability form.

The site monitor will periodically check the supplies of trial medication held by the investigator to verify the correct accountability of all trial medication used. At the end of the trial, all unused trial medication and all medication containers will be destroyed in case no other procedure is agreed.

Destruction of study medication can be arranged after permission by the sponsor.

The investigator will ensure that a final drug accountability report is prepared and archived in the Trial Master File at the trial site.

### Return of unused medication

Unused Ibrutinib and unused Venetoclax dispensed during previous visits must be returned to the site and drug accountability records updated at each visit. Returned drug must not be redispensed to anyone and must not be destroyed without prior written approval of Clinical Monitor/CRA. The monitor will advise when and where unused medication will be destroyed.

### Safety instructions

All participating sites will get an initiation visit and will be trained by the monitor. Safety und handling instruction will also be documented in the Trial Master File at the trial site***.***

### Procedures for monitoring subject compliance

Trial medication will be dispensed to the subjects by the investigator or his/her qualified staff in the individual site.

Subjects will be instructed to bring all unused trial medication to the trial site at every visit. Compliance will be assessed by counting of tablets. Details will be recorded on the drug accountability form in the Trial Master File at the trial site.

## Not permitted medication

Ibrutinib: Ibrutinib is metabolized primarily by CYP3A4. Avoid concomitant use of systemic strong or moderate CYP3A inhibitors and consider alternative agents with less CYP3A inhibition. If a strong CYP3A inhibitor must be used, e.g. for emergency treatment, reduce Ibrutinib dose to 140 mg or withhold treatment for the duration of inhibitor use. Subjects should be monitored for signs of Ibrutinib toxicity.

If a moderate CYP3A inhibitor must be used, reduce Ibrutinib to 140 mg for the duration of the inhibitor use. Avoid grapefruit and Seville oranges during Ibrutinib/placebo treatment, as these contain moderate inhibitors of CYP3A. No dose adjustment is required in combination with mild inhibitors.

Avoid concomitant use of systemic strong CYP3A inducers (e.g. carbamazepine, rifampin, phenytoin, and St. John’s Wort). Consider alternative agents with less CYP3A induction.

Consider the risks and benefits of anticoagulant or antiplatelet therapy when co-administered with Ibrutinib. Monitor for signs or symptoms of bleeding. Supplements such as fish oil and vitamin E preparations should be avoided. Ibrutinib should be held at least 3 to 7 days pre- and post-surgery, depending upon the type of surgery and the risk of bleeding.

For further information, please refer to the latest version of the Ibutinib information for health professionals. A list of common CYP3A inhibitors and inducers is provided in ANNEX II. Examples of inhibitors, inducers, and substrates can also be found at <http://medicine.iupui.edu/clinpharm/ddis/main-table/> . This website is continually revised and should be checked frequently for updates.

Venetoclax: Concomitant use of Venetoclax with strong CYP3A inhibitors at initiation and during the ramp-up period increases the risk for TLS. Concomitant use of Venetoclax with strong CYP3A inhibitors at initiation (7 days prior to first dose) and during the ramp-up period is contraindicated. For subjects who have completed the ramp-up period and are on a steady daily dose of Venetoclax, reduce the Venetoclax dose by at least 75% when strong CYP3A inhibitors must be used concomitantly. Avoid concomitant use of moderate CYP3A inhibitors with Venetoclax. Consider alternative treatments. If a moderate CYP3A inhibitor must be used, reduce the doses of Venetoclax by at least 50%. Monitor subjects more closely for signs of toxicities.

Avoid concomitant use of Venetoclax with strong CYP3A inducers (eg, carbamazepine, phenytoin, rifampin, St. John’s Wort) or moderate CYP3A inducers (eg, bosentan, efavirenz, etravirine, modafinil, nafcillin). Consider alternative treatments with less CYP3A induction.

For further information, please refer to the latest version of the Venetoclax information for health professionals. A list of common CYP3A inhibitors and inducers is provided in ANNEX II. Examples of inhibitors, inducers, and substrates can also be found at http://medicine.iupui.edu/clinpharm/ddis/main-table/. This website is continually revised and should be checked frequently for updates.

***Antiplatelet Agents and Anticoagulants***

*Ibrutinib*

Use Ibrutinib with caution in subjects requiring anticoagulants or medications that inhibit platelet function, and supplements such as fish oil and vitamin E preparations should be avoided during treatment with Ibrutinib. Bleeding events of any grade, including bruising and petechiae, occurred in patients treated with Ibrutinib and the mechanism for the bleeding events is not well understood. Subjects requiring the initiation of therapeutic anticoagulation therapy (eg, atrial fibrillation), consider the risks and benefits of continuing Ibrutinib treatment. If therapeutic anticoagulation is clinically indicated, treatment with Ibrutinib should be held and not be restarted until the subject is clinically stable and has no signs of bleeding. No dose reduction is required when study drug is restarted. Subjects should be observed closely for signs and symptoms of bleeding.

Surgery: Ibrutinib may increase risk of bleeding with invasive procedures or surgery. The following guidance should be applied to the use of Ibrutinib in the perioperative period for subjects who require surgical intervention or an invasive procedure while receiving Ibrutinib:

- *Minor Surgical Procedures:* For minor procedures (such as a skin or needle biopsy, lumbar puncture [other than shunt reservoir access], thoracentesis, or paracentesis) Ibrutinib should be held for at least 3 days prior to the procedure and should not be restarted for at least 3 days after the procedure. For bone marrow biopsies that are performed while the subject is on Ibrutinib, it is not necessary to hold Ibrutinib.
- *Major Surgical Procedures:* For any surgery or invasive procedure requiring sutures or staples for closure, Ibrutinib should be held at least 7 days prior to the intervention (except for emergency procedures) and should be held at least 7 days after the procedure and restarted at the discretion of the investigator when the surgical site is reasonably healed without serosanguineous drainage or the need for drainage tubes. The treatemment will be initiated at the same dose levels as prior to the intervention.

*Venetoclax*

In a drug-drug interaction study in three healthy subjects, administration of a single 400 mg dose of Venetoclax with 5 mg warfarin resulted in 18% to 28% increase in Cmax and AUC∞ of R-warfarin and S-warfarin, however, its use is – due to the aggravation of bleeding events seen with Ibrutinib – not allowed throughout study treatment.

*Bendamustine*:

Due to the risk of toxic skin reactions (e.g. Stevens-Johnson syndrome/toxic epidermal necrolysis), concomitant administration of allopurinol should be avoided. If allopurinol is indispensable, it should be stopped ≤24-48 hrs before and reinitiated ≥24-48 hrs after administration of Bendamustine.

Prohibited Concomitant anti-cancer Medications: Any non-study protocol-related chemotherapy, anticancer immunotherapy, experimental therapy, or radiotherapy are prohibited while the subject is receiving Ibrutinib treatment.

Corticosteroids for the treatment of the underlying malignancy are prohibited. The sponsor must be notified in advance (or as soon as possible thereafter) of any instances in which prohibited therapies are administered.

Vaccinations: Do not administer live attenuated vaccines prior to, during, or after treatment with Venetoclax. The safety and efficacy of immunization with live or attenuated viral vaccines during or following Venetoclax therapy have not been studied. Advise subjects that vaccinations may be less effective, however current recommendations should be followed. This holds especially true for the current pandemic situation where knowledge is continuously evolving.

# TRIAL SCHEDULE

Visits and examination should be performed according to table `Trial Schedule´ corresponding to the individually designated treatment arm.

Induction visits (cycle 1-6) may be shifted +/- 2 days due to organizational reasons.

Maintenance visits may be shifted +/- 1 week due to organizational reasons.

## Screening and Baseline: Days -28 until -1 (Arm A VR-I and Arm B BR-I)

The following information should be obtained:

- Patient information and informed consent
- Demographics: age, sex, ethnicity
- MCL characteristics: MIPI; MCL diagnosis according to WHO classification; Ann Arbor Stage; B-symptoms
- Prior/concomitant diseases
- Prior/concomitant medication
- Physical examination
  - Mandatory: LN-Status, Liver- and spleen-size, Lung/heart and abdominal status, basic neurological evaluation
  - Symptom oriented
- ECOG
- Vital signs (BP, heart rate, temperature, SaO2). Testing within 7 days prior first dose.
- Height, weight
- ECG - Testing within 7 days prior first dose
- Echocardiogram (recommended)
- Spirometry
- Lab General:
- Whole blood count and differential - Testing within 7 days prior first dose
- Creatinine/eGFR, uric acid, urea, ASAT, ALAT, sodium, potassium, calcium, phosphate - Testing within 7 days prior first dose
  - total bilirubin, gGT, total protein, electrophoresis and immunofixation in blood and urine
  - TSH, fT3, fT4, BSG, CRP, LDH, immunoglobulin levels (IgG), vitamin D, ferritin
- HIV, Hep B/C, EBV, CMV - Testing within 7 days prior first dose:
  - HIV-screen
  - anti-HBs, HBs-Ag, HBcAb, anti-HBc, HBV-DNA (if HBs-AG positive)
  - anti HCV; HCV-RNA (if anti HCV positive)
  - EBV serology
  - CMV serology
- Anti-COVID19 immunity: SARS-CoV-2 antibody response (IgG, IgM)
- CT neck/chest/abdomen/pelvis, evaluation following Lugano criteria. CT acceptable within 42 days before first dose
- (document PET in eCRF if available)
- Bone marrow biopsy: If a prior positive bone marrow result exists, no re-biopsy is necessary. Retesting during study is only necessary if was bone marrow infiltrated at screening
- MRD/ Immunreconstitution asservation (approx. 25ml, following Lab Manual)
- Shipping of diagnostic MCL biopsy sample (formalin fixed paraffin embedded) to national reference pathology (including Ki67 and p53 analysis)
- QoL questionnaires (EORTC QLQ-C30, EORTC QLQ-NHL-HG29) Testing within 7 days prior first dose. QoL will be handed out to the patients prior to seeing the doctor for clinical evaluations. Patients should complete the questionnaires by her/himself during the visit
- Geriatric assessment (ADL, IADL and CIRS-G) Testing within 28 days prior first dose. The questionnaires of ADL and IADL can be completed by a nurse, the questionnaire CIRS-G must be completed by a physician.
- EMCL/GLA-Registry (optional): Participants should be offered to participate in the EMCL/GLA-Registry. Participation in the registry is voluntary and does not affect MCL elderly III study participation but allows long term follow up

## Induction Treatment (Cycle length 28 days)

### **Day 1** of every induction cycle (Arm A VR-I and Arm B BR-I)

- Prior/concomitant medication
- Physical examination (Assessment not necessary, if done within 7 days before, physical examination can be performed symptom oriented, plus evaluation of LN-Status)
- ECOG
- Vital signs (BP, HR, temp., SaO2)
- Weight
- Hep B only at Cycle 2, 4 and 6, if patient was anti-HBc positive at screening:
  - HBV-DNA
- Adverse events

**And**

Laboratory Testings according to cycle and treatment arm and TLS risk profile:

- At Cycle1 Day 1 (**Arm A VR-I and Arm B BR-I**):

Lab Small

- - - Whole blood count and differential
    - Creatinine, uric acid, LDH, ASAT, ALAT, GGT, total bilirubin
- At Cycle 2 Day 1 (**Arm A VR-I**):
  - For patients with normal TLS risk 2x Lab tumorlysis must be done: pre-dose and 6–8 hours post the first dose of Venetoclax
    - Whole blood count and differential (pre-dose only)
    - Creatinine, uric acid, LDH, potassium, sodium, phosphate, calcium

(pre-dose, and 6–8 hours post dose)

- - - For patients with high-risk of TLS (s. section 7.2.4) 4x Lab tumorlysis must be done: pre-dose and 4 ±1h, 8 ±1h, and 12 ±1h post the first dose of Venetoclax
- Whole blood count and differential (pre-dose only)
  - Creatinine, uric acid, LDH, potassium, sodium, phosphate, calcium

(pre-dose, and 4 ±1h, 8 ±1h, and 12 ±1h post dose)

- At Cycle 2 Day 1 (**Arm B BR-I**):
  - Lab small:
    - Whole blood count and differential
    - Creatinine, uric acid, LDH, ASAT, ALAT, GGT, total bilirubin
- At Cycle 3-6 Day 1 (Arm A VR-I and Arm B BR-I):
  - Lab small
  - Whole blood count and differential
  - Creatinine, uric acid, LDH, ASAT, ALAT, GGT, total bilirubin

### **Day 2** Arm A VR-I only cycle 2; Arm B BR-I every induction cycle

- Prior/concomitant medication
- ECOG
- Vital signs (BP, pulse, temp., SaO2)
- Adverse events

**And** for Arm A VR-I cycle 2 only:

- Lab tumorlysis mandatory 24 ± 2 h after first intake of 20mg dose Ventoclax (Cycle 2 Day1), if needed also repeat 48 ±2h post dose.
  - Whole blood count and differential

(24 ± 2 h post first intake of 20mg dose Ventoclax)

- - Creatinine, uric acid, LDH, potassium, sodium, phosphate, calcium

(24 ± 2 h post first intake of 20mg dose Ventoclax).

Further assessment should be made according to good clinical practice on TLS as stated in the institutional standards

### **Day 8** only Arm A VR-I at induction cycle 1 and 2

- Prior/concomitant medication
- ECOG
- Vital signs (BP, HR, temp., SaO2)
- Adverse events

**And**

Laboratory Testings according to cycle and TLS risk profile:

- At Cycle 1 Day 8
  - Lab tumorlysis:
    - Whole blood count and differential
    - Creatinine, uric acid, potassium, sodium, phosphate, calcium, LDH
- At Cycle 2 Day 8
  - Lab small:
    - Whole blood count and differential
    - Creatinine, uric acid, LDH, ASAT, ALAT, GGT, total bilirubin
  - Additional for patients with high-risk of TLS (s. section 7.2.4) 2x Lab tumorlysis must be done: pre-dose and 6 - 8 h post dose of Venetoclax.

(Lab values already analyzied within “lab small” must not be repeated pre dose)

- - - Whole blood count and differential (pre-dose only)
    - Creatinine, uric acid, LDH, potassium, sodium, phosphate, calcium

(pre-dose and 6 - 8 h post dose)

### **Day 15** Arm A VR-I induction cycle 1 and 2; Arm B BR-I at induction cycle 1)

- Prior/concomitant medication
- ECOG
- Vital signs (BP, HR, temp., SaO2)
- Adverse events

**And**

Laboratory Testings according to cycle and treatment arm and TLS risk profile:

- At Cycle 1 Day 15 (VR-I and BR-I)
  - Lab small:
    - Whole blood count and differential
    - Creatinine, uric acid, LDH, ASAT, ALAT, GGT, total bilirubin
- At Cycle 2 Day 15 (VR-I)
  - Lab small:
    - Whole blood count and differential
    - Creatinine, uric acid, LDH, ASAT, ALAT, GGT, total bilirubin
  - Additional for patients with high-risk of TLS (s. section 7.2.4) 2x Lab tumorlysis must be done: pre-dose and 6 - 8 h post dose of Venetoclax.

(Lab values already analyzied within “lab small” must not be repeated pre dose)

- - - Whole blood count and differential (pre-dose only)
    - Creatinine, uric acid, LDH, potassium, sodium, phosphate, calcium

(pre-dose and 6 - 8 h post dose)

### **Day 22** Arm A VR-I induction cycle 1 and 2

- Prior/concomitant medication
- ECOG
- Vital signs (BP, HR, temp., SaO2)
- Adverse events

**And**

Laboratory Testings according to cycle and TLS risk profile:

- At Cycle 1 Day 22
  - For patients with normal TLS risk Lab tumorlysis 2x: must be done: pre-dose and 6-8h post the first dose of Venetoclax 20 mg
    - Whole blood count and differential (pre-dose only)
  - Creatinine, uric acid, LDH, potassium, sodium, phosphate, calcium

(pre-dose and 6-8 h post dose)

- - For patients with high TLS risk (s. section 7.2.4) 4x Lab tumorlysis must be done: pre-dose and at 4 ±1h, 8 ±1h, 12 ±1h
    - Whole blood count and differential (pre-dose only)
    - Creatinine, uric acid, LDH, potassium, sodium, phosphate, calcium

(pre-dose and at 4 ±1h, 8 ±1h, 12 ±1h )

Inpatient treatment is highly recommended for these patients

- At Cycle 2 Day 22
  - Lab small:
    - Whole blood count and differential
    - Creatinine, uric acid, LDH, ASAT, ALAT, GGT, total bilirubin
  - Additional for patients with high-risk of TLS (s. section 7.2.4)

2x Lab tumorlysis must be done: pre-dose and 6 - 8 h post dose of Venetoclax. (Lab values already analyzied within “lab small” must not be repeated pre dose)

- - - - Whole blood count and differential (pre-dose only)
      - Creatinine, uric acid, LDH, potassium, sodium, phosphate, calcium

(pre-dose and 6 - 8 h post dose)

### **Day 23** only cycle 1 Arm A VR-I

- Prior/concomitant medication
- ECOG
- Vital signs (BP, HR, temp., SaO2)
  - Lab tumorlysis mandatory 24 ± 2 h after t the first dose of Venetoclax 20 mg. For high risk patients if needed also 48 ±2h post the first dose of Venetoclax
    - Whole blood count and differential (24 ± 2 h post first 20 mg dose)
    - Creatinine, uric acid, LDH, potassium, sodium, phosphate, calcium

(24 ± 2 h post first 20 mg dose)

Further assessment should be made according to good clinical practice on TLS as stated in the institutional standards.

- Adverse events

### **Staging** prior induction cycle 4, Arm A VR-I and Arm B BR-I

- Prior/concomitant medication
- Physical examination (physical examination can be performed symptom oriented plus evaluation of LN-Status)
- ECOG
- Vital signs (BP, HR, temp., SaO2)
- ECG (if indicated)
- Lab General:
  - Whole blood count and differential
  - Creatinine/eGFR, uric acid, urea, ASAT, ALAT, sodium, potassium, calcium, phosphate, total bilirubin, gGT, total protein,
  - only if positive at screening: electrophoresis and immunofixation in blood and urine
  - TSH, fT3, fT4, BSG, CRP, LDH, immunoglobulin levels (IgG), vitamin D, ferritin
- CT Neck/Chest/Abdomen/Pelvis, evaluation following Cheson criteria
- (document PET in eCRF if available)
- Bone marrow biopsy (only necessary if bone marrow was infiltrated at screening)
- MRD/Immunreconstitution asservation (approx. 25ml, following Lab Manual)
- QoL questionnaires (EORTC QLQ-C30, EORTC QLQ-NHL-HG29) will be handed out to the patients prior to seeing the doctor for clinical evaluations. Patients should complete the questionnaires by her/himself during the visit
- Adverse events

If Interim Staging (prior cycle 4) indicates the end of induction, this Interim Staging Visit may be used as EoI visit. Assessments specific for EoI Visit should be performed additionally (e.g. echocardiogram).

### **End of Induction Visit EoI** (Arm A VR-I and Arm B BR-I)

All patients should perform EoI Visit within 3 weeks (± 7 days) after d1 of the last cycle or before maintenance or any new antineoplastic therapy.

Patients non-eligible for maintenance after EoI Visit, continue with follow up every 6 months.

- Prior/concomitant medication
- Physical examination (Assessment not necessary, if done within 7 days before; physical examination can be performed symptom oriented plus evaluation of LN-Status)
- ECOG
- Vital signs (BP, HR, temp., SaO2)
- Weight
- ECG (if indicated)
- Echocardiogram (recommended)
- Lab General
  - Whole blood count and differential
  - Creatinine/eGFR, uric acid, urea, ASAT, ALAT, sodium, potassium, calcium, phosphate, total bilirubin, gGT, total protein,
  - only if positive at screening: electrophoresis and immunofixation in blood and urine
  - TSH, fT3, fT4, BSG, CRP, LDH, immunoglobulin levels (IgG), vitamin D, ferritin
- Anti-COVID19 immunity: SARS-CoV-2 antibody response (IgG, IgM)
- CT Neck/Chest/Abdomen/Pelvis, evaluation following Cheson criteria
- PET
- Bone marrow biopsy (only necessary if bone marrow was infiltrated at screening)
- MRD/Immunreconstitution asservation (approx. 25ml, following Lab Manual)
- QoL questionnaires (EORTC QLQ-C30, EORTC QLQ-NHL-HG29) will be handed out to the patients prior to seeing the doctor for clinical evaluations. Patients should complete the questionnaires by her/himself during the visit
- Adverse events

## Maintenance Treatment

### **Maintenance Visit** every 8 weeks for 24 cycles (Arm A VR-I and Arm B BR-I)

Maintenance cycle length is 28 days, scheduled visits should be performed every second cycle (every 8 weeks). For organizational purposes in maintenance application can be shifted +/- 2 weeks. The next dose however should follow the initial schedule.

Maintenance should start directly in continuation of induction treatment. First Maintenance visit should be performed at day 57 after d1 of the last induction cycle.

- Prior/concomitant medication
- Physical examination (every 6 months, (physical examination can be performed symptom oriented plus evaluation of LN-Status)
- ECOG
- Vital signs (BP, HR, temp., SaO2)
- Weight
- ECG (if indicated)
- Lab small:
  - Whole blood count and differential
  - Creatinine, uric acid, LDH, ASAT, ALAT, GGT, total bilirubin
  - Hep B re-testing only if patient was anti-HBc positive at screening, HBV-DNA

(every second cycle: Mainenance cycle 2, 4, 6, and so on)

- Anti-COVID19 immunity: SARS-CoV-2 antibody response (IgG, IgM)

(only at week 56, about 1 year after EoI)

- CT Neck/Chest/Abdomen/Pelvis, evaluation following Cheson criteria.

(every 6 months, +/- 1 week from visit for organizational reasons)

- MRD/Immunreconstitution asservation (approx. 25ml, following Lab Manual every 24 weeks , starting visit at week 24 after EoI)
- QoL questionnaires (EORTC QLQ-C30, EORTC QLQ-NHL-HG29) will be handed out to the patients prior to seeing the doctor for clinical evaluations. Patients should complete the questionnaires by her/himself during the visit (at visit week 56 after EoI)
- Adverse events

### **End of Maintenance Visit** 24 months after EoI (Arm A VR-I and Arm B BR-I)

All patients who do not show progression during maintenance treatment should perform EoM Visit 24 months +/- 2 weeks after End of Induction visit (regardless from cycle delay). Patients with early maintanance termination (e.g. progression) should perform EoM within 4 weeks after last dose.

- Prior/concomitant medication
- Physical examination (Assessment not necessary, if done within 7 days before; physical examination can be performed symptom oriented plus evaluation of LN-Status)
- ECOG
- Vital signs (BP, HR, temp., SaO2)
- Weight
- ECG (if indicated)
- Lab small:
  - Whole blood count and differential
  - Creatinine, uric acid, LDH, ASAT, ALAT, GGT, total bilirubin
  - Anti-COVID19 immunity: SARS-CoV-2 antibody response (IgG, IgM)
- CT Neck/Chest/Abdomen/Pelvis, evaluation following Cheson criteria
- (document PET in eCRF if available)
- Bone marrow biopsy (only necessary if bone marrow was infiltrated at screening)
- MRD/Immunreconstitution asservation (approx. 25ml, following Lab Manual)
- QoL questionnaires (EORTC QLQ-C30, EORTC QLQ-NHL-HG29) will be handed out to the patients prior to seeing the doctor for clinical evaluations. Patients should complete the questionnaires by her/himself during the visit
- Adverse events
- Subsequent therapy

## Follow Up Phase

### **Follow Up Visit** every 6 months +/- 4 weeks (Arm A VR-I and Arm B BR-I)

All subjects who enter the trial will continue to be followed every 6 months for disease progression, subsequent treatment, and until at least two years after last patient last treatment up to a maximum of 5 years (LPLT).

- Physical examination (every 12 months, physical examination can be performed symptom oriented plus evaluation of LN-Status)
- ECOG
- Vital signs (BP, HR, temp., SaO2)
- ECG (if indicated)
- CT Neck/Chest/Abdomen/Pelvis, evaluation following Cheson criteria. 12 months after EoM and as necessary
- MRD/Immunreconstitution asservation (every 12 months, approx. 25ml, following Lab Manual)
- QoL questionnaires (every 12 months)
- Subsequent therapy

# TRIAL METHODS

## Assessment of efficacy

In general, analysis of efficacy of any anti-lymphoma treatment has been standardized worldwide during the last decades and it comprises evaluation of measurable lesions via radiologic investigations and the evaluation of non-measurable lesions with laboratory evaluations or bone marrow biopsy. Recently, positron-emission tomography has been added to international standards, which now result in the revised Lugano criteria, which is the accepted standard procedure (30). If PET is not available, criteria based on radiologic and laboratory evaluation are used, which have been the basis of the so called 2007 Cheson criteria (31).

The current Lugano staging system is given in Appendix 15.3.

- - Criteria for Response Assessment are based on the Lugano Classification

Table 5: Criteria for Response Assessment

| **Response and Site** | **PET-CT–Based Response** | **CT-Based Response** |
| --- | --- | --- |
| **Complete** | **Complete metabolic response** | **Complete radiologic response**  **(all of the following)** |
| Lymph nodes and extralymphatic sites | Score 1, 2, or 3* with or without a residual mass on 5PS†  (It is recognized that in Waldeyer’s ring or extranodal sites with high physiologic uptake or with activation within spleen or marrow (eg, with chemotherapy or myeloid colony-stimulating factors), uptake may be greater than normal mediastinum and/or liver. In this circumstance, complete metabolic response may be inferred if uptake at sites of initial involvement is no greater than surrounding normal tissue even if the tissue has high physiologic uptake) | Target nodes/nodal masses must regress to ≤ 1.5 cm in LDi  No extralymphatic sites of disease |
| Nonmeasured lesion | Not applicable | Absent |
| Organ enlargement | Not applicable | Regress to normal |
| New lesions | None | None |
| Bone marrow | No evidence of FDG-avid disease in marrow | Normal by morphology; if indeterminate, IHC negative |
| **Partial** | **Partial metabolic response** | **Partial remission (all of the following)** |
| Lymph nodes and extralymphatic sites | Score 4 or 5† with reduced uptake compared with baseline  and residual mass(es) of any size  At interim, these findings suggest responding disease  At end of treatment, these findings indicate residual disease | ≥ 50% decrease in SPD of up to 6 target measurable nodes and extranodal sites  (When a lesion is too small to measure on CT, assign 5 mm x 5 mm as the default value. When no longer visible, 0 x 0 mm) |
| Nonmeasured lesion | Not applicable | Absent/normal, regressed, but no increase |
| Organ enlargement | Not applicable | Spleen must have regressed by > 50% in length beyond normal |
| New lesions | None | None |
| Bone marrow | Residual uptake higher than uptake in normal marrow but reduced compared with baseline (diffuse uptake compatible with reactive changes from chemotherapy allowed). If there are persistent focal changes in the marrow in the context of a nodal response, consideration should be given to further evaluation with MRI or biopsy or an interval scan | Not applicable |
| **No response or stable disease** | **No metabolic response** | **Stable disease** |
| Lymph nodes and extralymphatic sites | Score 4 or 5 with no significant change in FDG uptake from baseline at interim or end of treatment | <50% decrease from baseline in SPD of up to 6 dominant, measurable nodes and extranodal sites; no criteria for progressive disease are met |
| Nonmeasured lesion | Not applicable | No increase consistent with progression |
| Organ enlargement | Not applicable | No increase consistent with progression |
| New lesions | None | None |
| Bone marrow | No change from baseline | Not applicable |
| **Progressive disease** | **Progressive metabolic disease** | **Progressive disease requires at least 1 of the following** |
| Individual target nodes/nodal  Masses  Extranodal lesions | Score 4 or 5 with an increase in intensity of uptake from baseline and/or  New FDG-avid foci consistent with lymphoma at interim or end-of-treatment assessment | PPD progression:  An individual node/lesion must be abnormal with:  LDi >1.5 cm and  Increase by ≥ 50% from PPD nadir and  An increase in LDi or SDi from nadir  0.5 cm for lesions ≤ 2 cm  1.0 cm for lesions > 2 cm  In the setting of splenomegaly, the splenic length must increase by > 50% of the extent of its prior increase beyond baseline (eg, a 15-cm spleen must increase to > 16 cm). If no prior splenomegaly, must increase by at least 2 cm from baseline  New or recurrent splenomegaly |
| Nonmeasured lesions | None | New or clear progression of preexisting nonmeasured lesions |
| New lesions | New FDG-avid foci consistent with lymphoma rather than another etiology (eg, infection, inflammation).  If uncertain regarding etiology of new lesions, biopsy or  interval scan may be considered | Regrowth of previously resolved lesions  A new node > 1.5 cm in any axis  A new extranodal site > 1.0 cm in any axis; if <1.0 cm in any axis, its presence must be unequivocal and must be attributable to lymphoma.  Assessable disease of any size unequivocally attributable to lymphoma |
| Bone marrow | New or recurrent FDG-avid foci | New or recurrent involvement |
| Abbreviations: 5PS: 5-point scale; CT: computed tomography; FDG: fluorodeoxyglucose; ICH: immunohistochemistry; LDi: longest transverse diameter of a lesion; MRI: magnetic resonance imaging; PET: positron emission tomography; PPD: cross product of the LDi and perpendicular diameter; SDi: shortest axis perpendicular to the LDi; SPD: sum of the product of the perpendicular diameters for multiple lesions.  * A score of 3 in many patients indicates a good prognosis with standard treatment, especially if at the time of an interim scan. However, in trials involving PET where de-escalation is investigated, it may be preferable to consider a score of 3 as inadequate response (to avoid undertreatment).  Measured dominant lesions: Up to six of the largest dominant nodes, nodal masses, and extranodal lesions selected to be clearly measurable in two diameters. Nodes should preferably be from disparate regions of the body and should include, where applicable, mediastinal and retroperitoneal areas.  Non-nodal lesions include those in solid organs (eg, liver, spleen, kidneys, lungs), GI involvement, cutaneous lesions, or those noted on palpation.  Nonmeasured lesions: Any disease not selected as measured, dominant disease and truly assessable disease should be considered not measured. These sites include any nodes, nodal masses, and extranodal sites not selected as dominant or measurable or that do not meet the requirements for measurability but are still considered abnormal, as well as truly assessable disease, which is any site of suspected disease that would be difficult to follow quantitatively with measurement, including pleural effusions, ascites, bone lesions, leptomeningeal disease, abdominal masses, and other lesions that cannot be confirmed and followed by imaging.  In Waldeyer’s ring or in extranodal sites (eg, GI tract, liver, bone marrow), FDG uptake may be greater than in the mediastinum with complete metabolic response, but should be no higher than surrounding normal physiologic uptake (eg, with marrow activation as a result of chemotherapy or myeloid growth factors).  †PET 5PS: 1: no uptake above background; 2: uptake ≤ mediastinum; 3, uptake > mediastinum but ≤ liver; 4: uptake moderately > liver; 5: uptake markedly higher than liver and/or new lesions; X, new areas of uptake unlikely to be related to lymphoma. | | |

## Other assessments

### Previous and intercurrent illnesses

Illnesses already known at the time of informed consent are to be documented in the CRF as medical history. Illnesses detected during the clinical trial are to be documented as Adverse Events (see chapter 9 Safety).

### Previous and intercurrent medical treatments

All medical treatments with the exception of the Investigational Medical Products received by the participant at the beginning and / or during the clinical trial are to be documented in the CRF as concomitant medication.

### Laboratory parameters and vital signs

Mandatory laboratory parameters and vital signs are to be documented in the CRF.

### Tumor Lysis Syndrome (TLS) Risk Assessment

All study subjects will be assessed for risk of developing TLS. Subjects at increased risk (high risk) are defined as having:

at least one lesion >10 cm;

or at least one lesion >5 cm and circulating lymphocytes >25,000 cells/mm3

and/or a reduced renal function eGFR <60 mL/min,

# SAFETY

## Definitions

Please see the standard definitions for Adverse Event (AE), Adverse (drug) reaction (AR), Unexpected Adverse (Drug) Reaction (UAR), Serious Adverse Event (SAE) and Suspected Unexpected Serious Adverse Reaction (SUSAR) in ANNEX I 15.1

### Documentation of special situations

Special situations such as study medication abuse, off-label use, misuse, overdose and medication errors (including significant dilution and infusion rate errors), lack of efficacy, any pregnancy exposure, exposure to medicinal product from breastfeeding, unexpected therapeutic or clinical benefit from use, or suspected transmission of any infectious agent via administration of a Janssen medicinal product (Ibrutinib) have to be documented in the source documents.

Any special situation with the investigational product occurring during the clinical trial must be reported in eCRF. Special situations should be reported in the eCRF within 3 business days after awareness by the investigator.

If the special situation leads to an adverse event, then only this adverse event has to be documented and reported as an AE/SAE

### Onset and end date of AEs and SAEs

The onset date of the AE is defined as the date when new signs or symptoms or worsening of a pre-existing condition first occur. The onset date of the SAE is defined as the date when at least one of the above listed criteria for seriousness occurs.

The end date of the AE is defined as the date when the symptoms resolve, or the event is considered stable by the investigator. The end date of the SAE is defined as the time the seriousness criteria are no longer applicable. The end date of the SAE must not be later than the end date of the corresponding AE. AEs and SAEs that are ongoing at the time of death are considered not resolved or resolving.

## Assessment of AEs by investigator (1^st^ assessment)

Subjects must be carefully monitored for adverse events by the investigator. The intensity of the adverse events and the causal relation to trial medication and/or procedures are to be assessed.

The terms “serious” and “severe” are not synonymous but are often used interchangeably. The term ‘severe’ is often used to describe the intensity (severity) of a specific event; the event itself, however, may be of relatively minor significance (such as severe headache).

This is not the same as “serious”, which is based on the existence of one of the regulatory defined seriousness criteria (see ANNEX I 15.1 Definition of SAE).

### Assessment of Intensity/Severity

The investigator will use the following definitions of severity in accordance with National Cancer Institute common terminology criteria for adverse events, CTCAE, version 5.0 [published on Nov 27^th^, 2017] to describe the maximum intensity of the adverse event.

GRADE Clinical description of severity

1 MILD adverse event

2 MODERATE adverse event

3 SEVERE adverse event

4 LIFE-THREATENING OR DISABLING adverse event

5 DEATH related to adverse event

### Assessment of Seriousness

See detailed definition of Serious Adverse Event in Chapter 15.1. As mentioned above, the criterion “serious” serves as guide for regulatory (expedited) reporting obligations.

If an AE

- results in death,
- is life threatening,
- results in hospitalization (overnight stay) or prolongation of existing hospitalization,
- results in persistent or significant disability or incapacity for the subject,
- is associated with a congenital anomaly or birth defect,

or

- qualifies as “other” medically significant event or condition at investigator’s discretion,
- it fulfills the definition of an Serious Adverse Event.

An SAE should be immediately (within 24 hours) reported to the sponsor after becoming aware of the event.

### Assessment of Causal relation to trial medication/procedures

Investigators should use their knowledge of the patient, the circumstances surrounding the event, and an evaluation of any potential alternative causes to determine whether or not an adverse event is considered to be related to the study drug, indicating yes or no accordingly. The following guidance should be taken into consideration:

- Temporal relationship of event onset to the initiation of study drug
- Course of the event, considering especially the effects of dose reduction, discontinuation of study drug, or reintroduction of study drug (as applicable)
- Known association of the event with the study drug or with similar treatments
- Known association of the event with the disease under study
- Presence of risk factors in the patient or use of concomitant medications known to increase the occurrence of the event
- Presence of non-treatment-related factors that are known to be associated with the occurrence of the event

For patients receiving combination therapy, causality will be assessed individually for each protocol-mandated therapy.

Relationship of the adverse events to the investigational products should be assessed as described in the following table.

Table 6: Relationship of adverse events to investigational products

| Is the adverse event suspected to be caused by the study drug on the basis of facts, evidence, science-based rationales, and clinical judegement? | |
| --- | --- |
| YES | There is a plausible temporal relationship between the onset of the adverse event and administration of the study drug, and the adverse event cannot be readily explained by the patient’s clinical state, intercurrent illness, or concomitant therapies; and/or the adverse event abates or resolves upon discontinuation of the study drug or dose reduction and, if applicable, reappears upon rechallenge. |
| NO | An adverse event will be considered related, unless it fulfills the criteria specified below. Evidence exists that the adverse event has an etiology other than the study drug (e.g. preexisting medical condition, underlying disease, intercurrent illness, or concomitant medication); and/or the adverse event has no pausible temporal relationship to administration of the study drug (e.g., cancer diagnosed 2 days after first dose of study drug). |

## Period of observation

In this trial, the period of observation for collection of adverse events extends from the first intake of study medication up to 30 days after last administration of an IMP.

If the investigator detects a serious adverse event in a trial subject before or after the end of the period of observation, and considers the event (at least possibly) related to the trial treatment or study medication, it should be documented and reported as SAE as described in 9.5.

## Documentation of AEs and Follow-up

Adverse events both serious and non-serious must be carefully documented in the source documents.

All AEs (whether serious or not) reported by the subject or detected by the investigator will be documented on the appropriate pages of the eCRF.

If the adverse event is serious (see Section 14.1), the investigator must complete, in addition to the “Adverse Event Page”, a “Serious Adverse Event Form” at the time the serious adverse event is detected.

Every attempt should be made to describe the adverse event in terms of a diagnosis. If a clear diagnosis has been made, individual signs and symptoms will not be recorded unless they represent atypical or extreme manifestations of the diagnosis, in which case they should be reported as separate events. If a clear diagnosis cannot be established, each sign and symptom must be recorded individually.

All subjects who have adverse events, whether considered associated with the use of the investigational products or not, must be monitored to determine the outcome. The clinical course of the adverse event will be followed up according to accepted standards of medical practice, even after the end of the period of observation, until a satisfactory explanation is found or the investigator considers it medically justifiable to terminate follow-up, but no longer than 30 days after the end of the trial.

Should the adverse event result in death, a full pathologist’s report should be supplied, if possible.

**Necessary criteria for adverse event evaluation by the investigator:**

- Event term or specification
- Duration (start date and stop date)
- Outcome
- Severity grad (according to CTCAE, see above)
- Seriousness of Event (according to seriousness criteria)
- Drug relationship of the AE to the investigational products (Causality assessment)

## Immediate reporting of SAEs by investigator

**SAEs must be immediately (at latest within 24 hours of the investigator’s awareness) reported via eCRF.**

In case of technical problems of the eCRF, the investigator will record the safety information on the provided paper Form, sign and send it to the immediately (at latest within 24 hours) to the

Pharmacovigilance department PV-KUM:

FAX No. +49 89 4400-7- 7900 / 7901

E-Mail: [PVKUM@med.uni-muenchen.de](mailto:PVKUM@med.uni-muenchen.de)

The investigator should provide additional information on the clinical course and the outcome of each Event as soon as possible (Follow up report).

## Safety evaluation and Reporting by sponsor

The sponsor will ensure that all legal reporting requirements are met. According to GCP the sponsor is responsible for the continuous safety evaluation of the investigational product(s) and the clinical trial.

On behalf of the sponsor PV-KUM will conduct the management of SAEs and the expedited reporting as required in legal requirements, following GCP regulation or after transition of the following trial EU Regulation 536/2014:

A suspected unexpected serious adverse reaction (SUSAR) will be reported to the competent authorities and the ethics committees as soon as possible but not later than 15 calendar days, and 7 calendar days if it was fatal or life-threatening. In latter case follow-up information is to be reported within further 8 days. All investigators and the members of the DMC will be informed within the same timeframes. The marketing authorization holder of the IMP should be informed too.

Work flow and procedures concerning Safety management will be described in a separate document.

Following GCP regulation:

Any safety issues requiring a re-evaluation of the benefit-risk relationship of the investigational medicinal product will also be reported to the competent authorities and the ethics committees as soon as possible but not later than 15 calendar days (GCP-Regulation § 13, section 4).

The Development Safety Update Report (DSUR) including a list of all serious adverse reactions will be submittted to the ethics committee(s), the competent authorities and the members of the DMC once a year.

After transition of trial to CTIS under EU Regulation 536/2014:

According to Article 53 (1) of EU Regulation 536/2014, the sponsor shall notify the Member States concerned through the EU portal of all unexpected events which affect the benefit-risk balance of the clinical trial, but are not suspected unexpected serious adverse reactions as referred to in Article 42. That notification shall be made without undue delay but no later than 15 days from the date the sponsor became aware of this event.

The Development Safety Update Report (DSUR) including a list of all serious adverse reactions will be submitted to Eudravigilance database and the members of the DMC once a year.

## Immediate Reporting of pregnancy by investigator

Any **pregnancy** diagnosed in a female subject or in the female partner of a male subject during treatment with the investigational product must be reported immediately using the “Pregnancy Reporting Form” to:

FAX No. +49 89 4400-7- 7900 / 7901

E-Mail: [PVKUM@med.uni-muenchen.de](mailto:PVKUM@med.uni-muenchen.de)

Pregnancy occurring during the clinical trial, although not considered a SAE, must be reported within the same timelines as a serious adverse event. The outcome of a pregnancy should be followed up carefully and abnormal outcome of mother or child should be reported if any.

## Emergency procedures

During and following a subject’s participation in the trial, the investigator should ensure that adequate medical care is provided.

# STATISTICS

Details of the statistical analysis of the data collected in this trial will be documented in a Statistical Analysis Plan (SAP) that will be generated by IBE Munich and finalized before closing the data base for every analysis*.* The SAP is based on the protocol including all amendments. The document may modify the plans outlined in this protocol; however any major modifications of the primary endpoint definition and/or its analysis will also be reflected in a protocol amendment. Any deviation from the original statistical plan must be described and justified in the final report. The statistical analysis will be conducted by means of SAS and/or R.

## Sample size

The primary objective is to compare 30-month FFS for each of the two treatment arms with the results previously observed in the first MCL Elderly trial with R-CHOP followed by R maintenance (Kluin-Nelemans et al., JCO 2020). For each treatment arm, a separate exact binomial hypothesis test will be performed using the following one-sided hypotheses:

Null hypothesis H_0_: 30-month FFS ≤ 60%

Alternative hypothesis H_A_: 30-month FFS > 60%

The significance level will be set to 10% accounting for the phase 2 design with limited sample size. For each of the two treatment arms we consider an improvement by at least 15% to at least 75% as clinically relevant and attainable to be detected with more than 90% probability (statistical power). With 67 evaluable subjects, an effective significance level of 0.0918 and a power of 90.7% is achieved when rejecting the null hypothesis if 46 or more patients are alive and failure-free at the 30-month time point. Allowing for 10% dropouts, 75 patients are needed, summing up to a total of 150 patients to be randomized in the trial.

## Analysis populations

All subjects who signed informed consent and those assigned a randomisation result are considered as enrolled, respective randomized subjects, even if they have not received any trial treatment.

All randomized subjects constitute the Intention-to-treat (ITT) population. This population is the primary analysis population. Within the ITT population analyses subjects will be assigned to the treatment to which they were randomised.

To be eligible for the per-protocol (PP) population, subjects must fulfil the following criteria:

- Inclusion criteria fulfilled and exclusion criteria not fulfilled according to available documentation
- Start of treatment according to the randomization result

The safety population comprises all subjects who received at least one dose of trial treatment. In analyses of the safety population subjects will be assigned to the treatment which they actually received.

## Efficacy analyses

The primary population for the analyses of efficacy is the ITT Population. Per protocol (PP) analyses will be performed for secondary efficacy analysis as indicated below.

### Definition and analysis of primary endpoint

The primary endpoint is failure-free survival status at the 30-month time point from randomization, classified as event yes/no with failure (stable disease at end of induction or progressive disease) or death as events. For the 30-month staging time point a time window of +/- 4 weeks is accepted. Patients alive at 30 months from randomization and without evaluable staging result at end of induction or at the 30-month time point will be excluded from the analysis (drop-outs). As multiple reasons are perceived for a missing staging result, no imputation method seems suitable and we prefer to base the primary analysis on observed data. We will perform a sensitivity analysis counting missing or non-evaluable staging result as failure. Furthermore, in the secondary analysis of failure-free survival as time-to-event endpoint earlier staging results will be included as censored observations.

Separately for each of the two trial arms, the frequency and the percentage of failure-free survival among all evaluable patients will be reported. A one-sided lower-bounded 10%-exact confidence interval will be calculated for the estimated failure-free survival probability in each treatment arm.

Separately for each of the two trial arms, a one-sided exact binomial superiority test will be performed with significance level 0.10 one-sided, as described in 10.1. In case of a different effective sample size as the planned number of 67, the critical value for rejecting the null hypothesis will be chosen maximal among the values with effective significance level ≤0.10. The two primary hypotheses for the two treatment arms are intended to answer separate questions whether or not each treatment is associated with superior outcome compared with the historical control of the preceding European MCL Elderly trial. Each of these questions could in principle be answered in separate trials. Therefore, no correction for multiple testing is necessary.

### Analysis of secondary endpoints

Failure-free survival is calculated from the date of randomization to the end of induction in case of stable disease to induction, to the date of progression or the date of death from any cause, whichever occurred first. Stable disease at end of induction, progression or death from any cause are defined events. For patients without event, failure-free survival will be censored at the latest follow-up showing the absence of an event. For patients without any restaging result or without any contact after randomization, failure-free survival is censored 1 day after randomization.

Progression-free survival is calculated from the date of randomization to the date of progression or death from any cause, whichever occurred first. For patients without documented progression and alive at last contact, progression-free survival is censored at the latest staging where progression was excluded. For patients without any staging result or without any contact after randomization, progression-free survival is censored 1 day after randomization.

Overall survival is calculated from the date of randomization to the date of death. For patients alive at last contact, overall survival is censored at last contact. For patients without any contact after randomization, overall survival is censored 1 day after randomization.

Complete remission (CR) and overall response (OR) rates are calculated as percentage of patients with CR and CR/PR, respectively among all patients with evaluable staging result at staging for end of induction. In addition, the best response achieved by month 6, 18, and 30 from the start of the treatment is recorded for each patient. Time to best response is calculated from the date of treatment start to the date of best response. For patients with the same response at all time points, the time of the first response recorded is considered as time to best response. Median time to best response will be summarized for each best response group (CR, PR, SD, PD). Time to first response is calculated from the date of treatment start to the date of first response (CR or PR). For patients with PD or death as their first response, time to first response is censored at the date of PD or death. For patients with SD as their best response, time to first response is censored at eventual first progression or at last contact in SD or at date of death. For patients without any response recorded after treatment start, time to first response is censored 1 day after the start of the treatment.

Secondary efficacy endpoints will be evaluated with descriptive statistical methods separately in each treatment group. Depending on the data type, absolute and relative frequencies, median, IQR, range, or Kaplan-Meier estimates will be calculated. For probabilities estimated by relative frequencies or the Kaplan-Meier method, two-sided 95% confidence intervals will be reported.

Failure-free survival, progression-free survival, overall survival, and time to first response as time-to-event variables will be exploratively compared between the two treatment arms using Cox regression. In unadjusted analyses and analyses adjusted for MIPI score and/or Ki-67 index (≥ vs. <30%), the hazard ratio between the two treatment arms will be estimated along with two-sided 95% confidence intervals. These results can only be interpreted as hypothesis generating, since the statistical power for a confirmatory evaluation will be unacceptably low. If, for example, BR-I achieves a 30-month FFS rate of 70%, corresponding to a hazard ratio of 0.70 in comparison to R-CHOP+R, 67 evaluable patients per group give rise to a power of only 52% to detect an improvement to 82% 30-months FFS with VR-I by a two-sided logrank-test with alpha=0.05. This improvement corresponds to a hazard ratio of 0.56 similar to the hazard ratio assumed for the sample size calculation for this trial corresponding to the improvement from 60% to 75%.

All secondary analyses will be performed according to both, ITT and PP. ITT analyses will be performed using the ITT cohort, evaluating patients in the respective randomized treatment group and without considering any protocol violations. PP analyses will be performed using the PP cohort, evaluating patients in the respective randomized treatment group and additionally censoring time-to-event endpoints for start of a new anti-lymphoma treatment without preceding treatment failure.

### Analysis of Subgroups

Explorative subgroup analyses may be performed for MIPI groups (combined low/intermediate group, high risk group), Ki-67 index (≥ vs. <30%), and MCL cytology (blastic/non-blastic) or any other relevant characteristic.

### Interim analyses

No interim analyses for the primary efficacy hypothesis tests. Before reaching the final sample size of evaluable patients, efficacy analyses will only be performed using pooled treatment arms.

## Analysis of adverse events

Treatment compliance will be described including the dose adherence, the frequency of treatment interruptions, and the percentage of patients completing treatment as planned.

All summaries and listings of safety data will be performed for the safety population stratified by treatment group.

AEs will be coded according to MedDRA terminology. Detailed information collected for each AE will include: A description of the event, duration, whether the AE was serious, intensity, relationship to trial drug, action taken and clinical outcome. Summary tables will present the number of subjects observed with AEs by MedDRA System Organ Class and Preferred Term and corresponding percentages stratified by treatment group. Additional subcategories will be based on event intensity and relationship to trial drug.

A subject listing of all AEs will be prepared.

Furthermore, dose adherence, number of treatment interruptions, percentage of patients completing treatment as planned will be analyzed.

## Analysis of quality of life

In the present study, health related quality of life (QoL) is a pre-specified key secondary endpoint. It is expected that overall QoL can be improved by the new treatment in the long-term and is not jeopardized in the short-term (during application of treatment).

### Instruments

QoL is a multidimensional construct, which can be defined as a state of general well-being reflecting physical, psychological, and social well-being and the impact of the disease and/or treatment related symptoms on daily-life functioning. The patient’s subjective perspective is an inherent component of QoL and is therefore best assessed via self-administered questionnaires.

Quality of life will be assessed with the following instruments:

- EORTC Quality of Life Core Questionnaire (QLQ-C30)
- EORTC Module for high-grade non-Hodgkin lymphomas (QLQ-NHL-HG29)

#### QLQ-C30

The EORTC QLQ-C30 version 3 is composed of multi-item and single-item scales. These include five functional scales (physical, role, emotional, social, and cognitive), three symptoms (fatigue, nausea and vomiting and pain) and a global health status/QoL scale and six single items (dyspnea, insomnia, appetite loss, constipation, diarrhea and financial difficulties). The scoring algorithm for the scales results in a score from 0 to 100 where a high score for a functional scale represents a healthy level of functioning, but a high score for a symptom scale represents a high level of problems. All scales and single items meet the standards for reliability. The reliability and validity of the questionnaire is highly consistent across different language-cultural groups (32). (The average time to complete the questionnaire is approximately 10 minutes.

#### QLQ-NHL-HG29

This module is to be used in conjunction with the QLQ-C30. It is a standardized instrument for measuring quality of life in patients with high-grade non-Hodgkin lymphomas. It consists of 29 items which can be summarized into the following scales: Symptom burden due to disease and/or treatment, Neuropathy, Physical condition/Fatigue, Emotional impact and Worries/fears about health and functioning (33). The scoring algorithm for the QLQ-NHL-HG29 is similar to the QLQ-C30.

The following scales will be used for statistical tests: Global QoL (from the QLQ-C30), Physical condition/fatigue and Symptom burden (from the QLQ-NHL-HG29). The remaining scales will be analysed on an exploratory basis.

### Data collection and Monitoring of QoL compliance

The case report forms (CRF) will include a question whether the QoL forms have been filled in, and if not, the reason why. The questionnaire will be handed out to the patients by the investigator or a study nurse prior to seeing the doctor for clinical evaluations. The patient should complete the questionnaires by her/himself during the visit to the outpatient clinic as completely and accurately as possible. It is recommended that a key person (e.g. research nurse) at each site should be responsible for questionnaire data collection in order to optimize the compliance of the patient and to ensure the completeness of the data. The completed questionnaires will be entered by the local site personel into the eCRF.

During the study, compliance with completing questionnaires will be investigated at each time point (compliance is defined as number filled/number eligible at this time point). The compliance of the QoL assessments will be reviewed twice a year and will be part of the descriptive report.

### QoL schedule

The time windows for eligible QoL assessments will be as described in the following table.

Table 7: QoL assessments

| **Assessment** | **Target date** | **Time window** |
| --- | --- | --- |
| Baseline (t1) | Within 7 days prior to randomization | No earlier than 28 days prior to randomization. No later than the day of randomization itself; the day of randomization included. |
| Staging prior to cycle 4 (t2) | Date of staging | No earlier than 1 week before and no later than 2 weeks after the staging date. |
| End of Induction EoI (t3) | At week 3 after last combination therapy | No earlier than 1 day and no later than 8 weeks after the date of the final dose |
| 1 year after EoI (t4) | 12 months after End of Induction | No earlier than 1 month before and no later than 2 months after the target date |
| Every 12 months until year 4 thereafter (t5-t7) | During maintenance and for one more year thereafter | No earlier than 2 months before and no later than 4 months after the target date |

QoL forms are considered as invalid if either all questions on the form are blank, the completion date is unknown or it cannot be assigned to a single assessment time point or the completion date falls outside the time windows. When multiple forms are received during the same time window, the form closest to the intended assessment time will be kept.

The above time windows will be evaluated at the time of the analysis. The compliance of the QoL forms will be calculated and the number of invalid forms will be summarized by reason overall (not by treatment arm). The time windows may be readjusted to optimize compliance prior to revealing any QoL score data.

### QoL analysis

#### Power considerations

The key QoL endpoint for this study is the global health/QoL scale (GHS) of the EORTC QLQ-C30. The following scales from the EORTC QLQ-NHL-HG29 will be considered as secondary QoL endpoints: Physical condition/fatigue and Symptom burden. The other available QoL scales will only be analyzed on an exploratory basis. The financial difficulties item will not be analyzed.

The GHS will be used as primary outcome of interest for this study. A difference of 10 points on the 100-point QLQ-C30 scale between the two arms will be considered as clinically relevant. The standard deviation of this scale is approximately 20 points. With the 2-sided alpha set at 5% and a power of 80% to detect a difference of 10 points (effect size of 0.5), a minimum of 128 patients (64 per treatment arm) is required. For an effect size of 0.75 (difference of 15 points), 56 patients (28 per treatment arm) are required. Therefore, this study is sufficiently powered to detect differences in QoL.

#### Primary analysis

The following analysis will be conducted in the overall population according to the ITT principle.

Two summary scores will be calculated per subject for the global health/QoL scale to reflect the short term and the long-term objective:

- *the average change at end of induction:* This will be calculated by subtracting the baseline score from the score reported at end of induction. If either of the scores are missing, this statistic is considered to be missing.
- *the average change at end of maintenance:* This will be calculated by subtracting the baseline score from the score reported at end of maintenance. If either of the scores are missing, this statistic is considered to be missing.

Non-parametric rank-order tests and mixed-models regression analysis will be performed using a 2-sided significance level of 5% to test for significant differences between the treatment arms for both statistics. Description will be presented (mean, standard deviation, median, first and third quartiles, minimum, maximum) by treatment group together with mean difference, 95% CI and the non-parametric test p-value.

Absolute differences in QoL scores will be related to the established 10-point minimal clinically important difference in order to supplement statistical significance with a clinical anchoring.

#### Secondary analysis

The same procedure as described under 10.5.4.2 will be performed with the 2 scales defined to acta s secondary endpoints (physical condition/fatigue and symptom burden)

#### Supportive analysis

Time to improvement is calculated for each of the selected scales (primary and secondary QoL endpoints) as the first post baseline time where the patient reports a value equal to or better than the baseline score. For patients never reporting such value, their time to recovery is censored at the last known QoL assessment time.

Time to improvement is summarized and compared between the two treatment arms using Kaplan-Meier methodology and logrank tests.

#### Exploratory analysis

The scores for all remaining QoL domains in the two arms at each assessment time-point will be presented descriptively (mean, standard deviation, median, first and third quartiles, minimum, maximum). 95% CI will also be calculated, and a graphical display of the patterns of change over time will be provided.

#### Missing data

Missing data is a potential major source of bias in QoL assessment.

In order to check the potential impact in the study, the compliance mechanism will be investigated prior to initiating the QoL analysis. The compliance between the 2 arms will be compared at each post-baseline time point using a chi-square test. In order to adjust for the multiplicity of the tests, a Bonferroni adjustment will be made.

The following outcomes will be presented per time-point:

- - compliance with QoL assessment by treatment group and overall
  - percentage of questions answered by treatment group (summary statistics)
  - comparison of post-baseline compliance (chi-square test or fisher exact test (if cells < 10))

In case significant differences are found, explanation of these differences should be sought and the analysis strategy adapted accordingly. Characteristics of patients with and without valid QoL data will be compared and trends over time per dropout pattern will be investigated. Model building will be used in order to investigate whether the compliance mechanism is linked to selected prognostic variables.

Imputation

Missing (ie. expected but not received) QoL values will be imputed via a general linear regression model (GLM) for the QoL scales. A GLM with identity link, normal distribution of errors and least square-estimation will be fitted to the available data (all valid QoL observations from all timepoints). The following independent covariates will be included: treatment arm, time point, institution, gender, and age.

Prior to building the model, the appropriateness of each covariate with respect to distribution, sparsity and missing data will be assessed. In addition, intercollinearity between the different covariates will be summarized. Covariates may be excluded from inclusion or reparametrized based on these findings. The resulting model will be used to predict imputed values for the missing QoL data whereby values outside the 0-100 range will be truncated to 0 or 100. These completed data will be analyzed similar to the main method as sensitivity analyses to assess the stability of the main results vis-à-vis the missing data.

In case overall compliance is deemed too low (<50%), only an exploratory analysis will be performed in lieu of the main analysis.

## Geriatric assessment evaluation

Older patients of a similar age are extremely heterogeneous, considering comorbidities, functional capacities, psychological and physical reserves and their evaluation through a geriatric assessment (GA) is now recommended by scientific communities (34, 35). This instrument was demonstrated to be able to predict severe treatment-related toxicity and overall survival (OS) in a variety of tumours and treatment settings(36).

Recently, it was recommended that prospective clinical trials should include a geriatric and comorbidity score as an obligatory component of the diagnostic work-up (35). This will be an important first step to correlate treatment toxicity and efficacy with fitness in patients with lymphoma, opening the way to a future tailored treatment approach for each individual patient.

A simplified geriatric assessment (sGA) was recently validated by FIL in a large prospective study addressed to DLBCL patients and an Elderly Prognostic Index was built (37). A pilot study for the application of this score in mantle cell lymphoma (MCL) is in progress.

A specific interest in the application of GA in MCL patients comes from the promising results obtained with chemo-free regimens as a first-line or second-line treatment, with good tolerability and a favourable safety profile (e.g bruton tyrosine kinase inhibitor, Ibrutinib). An attempt was made to speculate whether vulnerable patients may particularly benefit from these new drugs (38). As no data are actually available from clinical trials in patients with MCL, we propose an exploratory evaluation according to a sGA.

# QUALITY CONTROL, QUALITY ASSURANCE AND RISK MANAGEMENT

The sponsor will continuously manage quality and risks to ensure subject protection and reliability of trial results. The measures used will be proportionate to the inherent risks and the importance of the data collected.

## Requirements for Investigator, investigational sites and members of the investigating staff

The investigator should be able to demonstrate (e.g. based on retrospective data) a potential for recruiting the required number of suitable subjects within the agreed recruitment period.

The investigator should have sufficient time to properly conduct and complete the trial within the agreed trial period. The investigator should have available an adequate number of qualified staff and adequate facilities for the foreseen duration of the trial to conduct the trial properly and safely.

The investigator has to select his investigating team and ensure that all members are adequately qualified, informed about the protocol, any amendments to the protocol, the trial treatments, and their trial-related duties and functions. Furthermore, he must nominate an adequately qualified deputy.

If the investigator/institution retains the services of any individual or party to perform trial-related duties and functions, the investigator/institution should ensure this individual or party is qualified to perform those trial-related duties and functions and should implement procedures to ensure the integrity of the trial-related duties and functions performed and any data generated.

The investigator must be able to show a training for EU regulation 536/2014 as this trial will be transtioned to CTIS

## Quality of the source data

The investigator/institution should maintain adequate and accurate source documents and trial records that include all pertinent observations on each of the site’s trial subjects.

Source data should be attributable, legible, contemporaneous, original, accurate, and complete. Changes to source data should be traceable, should not obscure the original and should be explained if necessary (e.g., via an audit trail).

## When a copy is used to replace an original document, the copy has to fulfill the requirements for certified copies.Direct access to source data/documents

The investigator/institution must permit trial-related monitoring and auditing by authorized personnel of the Sponsor, as well as inspections by the appropriate competent authorities, and provide direct access to source data/documents (Confidentiality see chapter 12.4).

The subjects will be informed that representatives of the sponsor and/or competent authorities may inspect their medical records to verify the information collected, and that all personal information made available for inspection will be handled in strictest confidence and in accordance with local data protection laws.

## Monitoring

A combination of on-site monitoring and centralized monitoring will be performed. Remote source date verfication may be conducted risk-based, in compliance with data protection law following the approval of competent authorities.

On-site monitoring will be done by personal visits from a clinical monitor. To initiate the trial, the monitor shall ensure that the investigators and members of the investigating staff understand all requirements of the protocol and their regulatory responsibilities.

The monitor will ensure that the investigator will maintain a list of members of the investigating staff to whom they have delegated significant trial-related duties (“delegation log”).

Each site will be visited by the monitor at regular intervals to ensure compliance with the trial protocol, GCP and legal aspects. The monitor will review the entries into the eCRFs for completeness and correctness and verify the entries on the basis of the source documents. The presence of correct informed consents will be checked for every subject. By frequent communications (letters, telephone, fax), the monitor will ensure that the trial is conducted according to the protocol and regulatory requirements.

The investigator must allow the monitor to look at all relevant documents and must provide support at all times to the monitor.

To ensure continuous oversight and risk management by the sponsor between the monitor visits and for preparation of the monitor visits, the monitor or other sponsor staff adequately qualified and trained (e.g. data managers) will check the entries in the eCRFs at regular intervals (centralized monitoring). The intervals and the items to be checked will be specified in the monitoring manual and/or related documents (e.g. data management plan, safety management plan). Centralized monitoring will help to identify erroneous and potentially unreliable data and sites/processes for targeted on-site monitoring.

Details and the rationale for the chosen monitoring strategy will be specified in the monitoring manual for this trial.

## Risk Management

During protocol development and the entire course of the trial, all potential and emerging risks (e.g. delayed eCRF data entry) to subject protection and reliability of the trial results will be closely monitored, and preventive and corrective measures will be specified. The sponsor will identify, evaluate, control, communicate, review and report the relevant risks periodically to ensure continuous and timely risk management.

## Measures to secure compliance

Any significant noncompliance with the protocol, SOPs, GCP and regulatory requirements by any party involved in the conduct of the trial will be analyzed (e.g. root cause analysis) by the sponsor, and appropriate corrective and preventive actions will be implemented.

## Inspection by authorities

Competent authorities may request access to all source documents, eCRF, and other trial documentation in case of an inspection. Direct access to these documents must be guaranteed by the investigator who must provide support at all times for these activities. Source data documents can be copied during inspection, provided the identity of the subject have been made unrecognizable.

## Audits

Persons (auditors) authorized by the sponsor may request access to all source documents, CRF, and other trial documentation in case of an audit. Direct access to these documents must be guaranteed by the investigator who must provide support at all times for these activities. Source data documents can be copied during an audit, provided the identity of the subject have been made unrecognizable.

# DATA MANAGEMENT

## Responsibilities

The investigators are responsible for complete and correct documentation in a timely manner. Investigators may delegate documentation to other site personal, but they remain responsible for checking the documentation for completeness and correctness in a regular way. The Data management team is authorized in the case of discrepancies or correction of data errors to contact direct the responsible person at trial site. Queries will be created in the eCRF and should be answered within a time of two weeks if no other timeline is specified. The site is responsible for regular checking the eCRF for Queries. Queries and answers remain part of the electronic trial documentation. All members of the investigating team have to agree to be contacted by e-mail and by phone about the documentation.

A detailed methodology for the data management in this trial will be documented in a data management plan (DMP) that will be dated and maintained by the study center in Munich. This plan has to be signed by the sponsor (or a sponsor representative), the head of the data management team and the responsible data manager. The document may modify the plans outlined in this protocol; however, any major modifications of the data handling will also be reflected in a protocol amendment.

## Data collection

In this clinical study, the GCP compliant Electronic Data Capture (EDC) System “MARVIN” by XClinical will be used to implement the eCRF and the related study database. Before any data entry is performed, the eCRF will be validated according to the requirements for computerized systems used in clinical studies, and the technical specifications will be documented. In event of future protocol amendments their impact on the eCRF has to be verified. If necessary the eCRF will be modified adequately and the changes will be validated.

The investigator has ultimate responsibility for completeness, accuracy, authenticity as well as timely collection and reporting of all clinical, safety, and laboratory data in the eCRF. All data may only be entered into the eCRF by authorized qualified study personnel. The study sites should provide the sponsor with the valid and up to date list of persons to whom data entry currently has been assigned. The sponsor will make sure that these persons receive an adequate training in a timely manner with documented license for controlled access to the production eCRF and are provided with written data entry and processing guidelines. The study sites will be informed to contact the study center for assistance. A separate eCRF manual is available to support the data entry.

The investigator, or designated representative, should complete the eCRF pages as soon as possible after information is collected, preferably within two weeks after a study patient is seen for an examination, treatment, or any other study procedure. Any outstanding entries must be completed immediately after the final examination. An explanation should be given for all missing data.

Data will be collected on the eCRF according to this protocol and providing the essential data quality to document eligibility, safety, and efficacy parameters, compliance to treatment schedules, and parameters necessary to evaluate the study endpoints. Any corrections to entries made in the source documents must be dated, signed, and explained (if necessary). Any corrections to entries made in the eCRF must be explained. All entries and corrections on the eCRF are automatically documented via “audit trail” provided by the EDC system. The data in the eCRF must match with the data in the source documents. Inconsistencies will be queried and discussed with the investigator. In most cases, the source documents are the hospitals’ or the physician's subject chart. In these cases data collected on the eCRF must match the data in those charts. After data clearance the data base will be locked against further changes, and data will be used for statistical analysis.

The monitor is responsible to verify the eCRF data at regular intervals throughout the study to verify the adherence to the protocol, completeness, accuracy, and consistency with the source data. Therefore, the monitor should have access to subject medical records and other study-related records needed to verify the entries on the eCRF. The investigator agrees to cooperate with the monitor to ensure that any problems detected in the course of the monitoring visits, including delays in completing eCRF, are resolved and will be avoided in future.

The investigator has to sign the Signature Form for this study on eCRF to confirm the completeness, accuracy, and authenticity of all data entered in the eCRF for the patient.

## Data handling

During data entry integrity checks help to minimize entry failures. Checks for plausibility, consistency and completeness of the data will be performed continuously by datamanagement and medical review during the trial. Based on these checks, queries will be raised. Any missing data or inconsistencies will be reported back to the respective site, if necessary with additional internal messages, and will have to be clarified by the site team under supervision of the responsible investigator. After completion of data entry and if no further corrections are to be made in the database, the access rights will be withdrawn and the database will be declared closed and ready to be used for statistical analysis. All data management activities will be done according to the current Standard Operating Procedures (SOPs) of the study center in Munich.

## Storage and archiving of data

The investigator will be provided with a Investigator Site File/ clinical Trial Master File (cTMF) at the start of the trial. All source data and all essential documents will be kept filed according to applicable legal regulations and to the ICH-GCP guideline. After termination of the trial the trial master file will be archived for 25 years, (anticipating EU Regulation 536/2014), the medical records of subjects in accordance with national law.

The cTMF includes the subject identification list, where the investigator has to record the study participation of each subject. This list allows the identification of each subject and contains the subject-id given by sponsor, the name, telephone number (if applicable), birth date and the date of inclusion of the subject into the study, and will be reviewed by the monitor for completeness. After the end of the study, the subject identification list remains at the study site. In addition, study participation of the subject should be recorded in the subject chart (study drug, subject-id given by sponsor, start and end date of the study).

The investigator should maintain a list of appropriately qualified persons to whom he/she has delegated study duties. This list will be provided with the TMF, too.

Furthermore, study personnel responsible for documentation in the eCRF should be identifiable. Therefore, a signature log with the name, signature, initials/abbreviation and study responsibilities of all persons who are allowed to make entries into the eCRF will be filed in the investigator’s site file.

The study documents provided by the sponsor are confidential and may not be made accessible to third parties not involved in the study by the investigator or other staff members. All study data are collected pseudonymously.

The sponsor and investigator/institution should maintain a record of the location(s) of their respective essential documents including source documents. The storage system used during the trial and for archiving (irrespective of the type of media used) should provide for document identification, version history, search, and retrieval.

After completion of the trial all electronic data will be handed over to the sponsor. For all sites the electronic data entries in the eCRF by the site (including audit trail) will be archived on electronic media for archiving at the trial site.

## Long Term follow up of Patients of the MCL-Elderly-III-trial in the EMCL-Registry

Follow up of clinical research projects is limited to a distinct time period, following current regulations. However, the analysis of subsequent therapies, late complications and overall survival is of special merrit, especially in the light of the dynamic change of current treatment algorithms. To cope with the lack of information on the further disease course, all patients will be asked to consent separatly to participate in the EMCL-Registry (or legal successors, e.g. GLA-R for German sites). During treatment within the MCL elderly III trial, information will be entered or exchanged in both the eCRF and the EMCL-Registry either decentrally or centrally.

# ETHICAL AND LEGAL ASPECTS

## Good clinical practice

The procedures set out in this trial protocol will be performed according to the worldwide-recognized principles of good clinical practice (GCP; as defined in the ICH-E-6 R2 Guideline and in compliance with the ethical principles described in the current version of the Declaration of Helsinki.

## Legal Requirements

This trial will be conducted following GCP regulation and after transition of the following trial EU Regulation 536/2014. Transition of trial to CTIS is planned for 2023. All investigators will be informed about approval of concerned member state.

Furthermore, the requirements of the Data Protection Regulation (EU) No 2016/679 will be adhered to. If supplementary national legislation is applicable, this should be observed.

## Subject information and informed consent

Before being admitted to the clinical trial, the subject must consent to participate after being fully informed by the investigator or a designated member of the investigating team about the nature, importance, risks and individual consequences of the clinical trial and their right, to terminate the participation at any time.

The subject should also have the opportunity to consult the investigator, or a physician member of the investigating team about the details of the clinical trial. The informed consent to participate in the clinical trial may be withdrawn by the subject verbally in the presence of, or in written form directed to, the investigator or a physician member of the investigating team at any time during the trial. The subject must not entail any disadvantage therefor or be coerced or unduly influenced to continue to participate. Furthermore, the subject is not obligated to disclose reasons for the withdrawal of the consent.

A copy of the signed informed consent document must be given to the subject. The original signed consent document will be retained at the trial site.

The investigator must not undertake any measures specifically required for the clinical trial until valid consent has been obtained.

If the subject has a primary physician, the investigator should inform him or her about the subject’s participation in the trial, provided the subject agrees hereto.

After reading the informed consent document, the subject must give consent in writing. The subject's consent must be confirmed by the personally written name and personally dated signature of the subject and by the personally written name and personally dated signature of the physician conducting the informed consent discussion.

If the subject is unable to write, oral presentation and explanation of the content of the subject leaflet, of the informed consent form and of the data protection information must take place in the presence of an impartial witness. The witness and the physician conducting the informed consent discussions must also sign and personally date the consent document. The witness must not be in any way dependent on the sponsor of the trial, the trial site or any member of the investigating team (e. g. an employee at the trial site).

If a subject is not able to give informed consent, e.g. due to their physical or mental condition, the consent of a legally authorized representative* must be sought. The consent must be confirmed by the personally dated signatures of both the representative and the physician conducting the informed consent discussion. A copy of the signed consent document must be given to the representative. The original signed consent document will be retained at the trial site. If possible, informed consent must be sought from the subject as soon as he regains the ability to give consent. This procedure must have prior agreement from the independent ethics committee.

## Confidentiality

The names of the subjects and other confidential information are subject to medical professional discretion data protection according to Data Protection Regulation; (EU 2016/679). If supplementary national legislation is applicable, this should be observed. The name of the subjects and other confidential information will not be disclosed to the sponsor.

During the clinical trial, subjects will be identified solely by means of an individual identification code (e.g. subject number, randomization number). The investigator will maintain a personal subject identification list (subject numbers with the corresponding subject names) to enable records to be identified.

Trial data (electronic and in paper form) will be handled in strictest confidence. Security procedures will be implemented to prevent disclosure of data to unauthorized persons. The appropriate regulations of data legislation will be fulfilled in its entirety.

The subject will declare in the written consent to release the investigator from the medical professional discretion to enable the attribution of the trial data in case of inspections by health authorities, audits by the sponsor and data monitoring by authorized sponsor representatives (monitors).

## Responsibilities of the investigator

‘Principal investigator’ means an investigator who is the responsible leader of a team of investigators who conduct a clinical trial at a clinical trial site.

The principal investigator nominates adequately qualified members of the investigating team and must instruct and supervise them in order to ensure that they are adequately informed about relevant information regarding the trial, especially the trial protocol and substantial modifications. Changes of principal investigators are substantial modifications and have to be approved by applicable authorities. The principal investigator has to inform the sponsor in advance, if changes will take place.

The principal investigator ensures the suitability of the trial site. The principal investigator will maintain a list of the members of the investigating team and other persons to whom they have delegated significant trial-related duties (“delegation log”).

The responsibilities of the investigator with regard to reporting of adverse events are described in chapter 7 (“Safety”).

## Documentation of serious breaches

A ‘serious breach’ means a breach likely to affect to a significant degree the safety and rights of a subject or the reliability and robustness of the data generated in the clinical trial.

Suspected serious breaches have to be documented in the source documents.

Any suspected serious breach occurring during the clinical trial must be reported without undue delay using the report form for suspected serious breachs to:

[ssb-MCLe3@izks-mainz.de](mailto:ssb-MCLe3@izks-mainz.de)

The initial serious breach report should be as complete as possible including: subject’s identification (screening number/random number), if applicable; name of reporter/institution; description of the suspected serious breach; planned corrective and preventive action, if applicable.

The reporter should provide additional information on further action as soon as possible (Follow up report), if applicable.

The sponsor will evaluate if the event reported fulfills the criteria of a serious breach.

Cases will be collected and processed following ICH-GCP. After transistion of trial to EU Regulation 536/2014 the sponsor has to notify the concerned Member States about a serious breach via the EU portal without undue delay but not later than seven calendar days of becoming aware of that breach.

## Approval of trial protocol and amendments

### Submissions

Before the start of the trial, the sponsor submits a written application for trial approval to the competent authority as well as for the favorable opinion of the ethics committee, responsible for the coordinating investigator. Local ethics committees, responsible for the other trial sites receive a copy of the application. Additionally, the local ethics committees receive detailed information on the respective trial sites and the investigators in order to confirm their qualification.

Investigational products can only be supplied to the trial sites after the fulfillment of all ethical and legal requirements for initiation (e.g. needed approvals are granted) of the clinical trial has been confirmed on behalf of the sponsor (“regulatory greenlight”).

### Substantial Amendments/Modifications

Neither the investigator nor the sponsor may alter this trial protocol without obtaining the written agreement of the other party. Subsequent changes – except purily editorial changes, e.g. the correction of mis-spellings - to the protocol during an ongoing trial have to be implemented via protocol amendments. The sponsor is responsible for obtaining the approval for the substantial modification.

Trial sites joining the ongoing trial have to undergo the approval procedure at their ethics committees or the equivalent procedure following EU Regulation 536/2014 after transition of the trial.

### Other information to the concerned member states

The sponsor is responsible for the ongoing evaluation of the safety of the IMP and the participants of the trial; the corresponding notifications in accordance with § 11 and § 13 GCP-V are described in chapter (“Safety”).

After transition the sponsor will proceed all notifications according REGULATION (EU) No 536/2014 (Art 36-38) in CTIS. The sponsor shall permanently update in CTIS any information on changes to the clinical trials which are not substantial modifications but are relevant for the supervision of the clinical trial by the Member States concerned (Art. 81). Relevant correspondence with the concerned member states will be properly archived by the sponsor.

## Data monitoring committee (DMC)

A data monitoring committee consisting of two clinical and one statistical experts will be established by the sponsor to review the safety data. The DMC will monitor the progress of the trial and advice whether to continue, modify, or stop this trial. Details will be specified in the DMC charter for this trial.

## Insurance

The sponsor (or a designated representative of sponsor) has effected an insurance policy covering his legal liability for injuries caused to participating persons and arising out of the trial procedures

Any impairment of health which might occur in consequence of trial participation must be immediately reported to the insurance company by the subject concerned; if applicable, after consulting the investigator. The insured person will consent to and comply with all appropriate measures serving for clarification of the cause and the extent of damage as well as the reduction of damage.

During the conduct of the trial, the subject must not undergo other clinical treatment except in cases of emergency. The subject is obliged to inform the investigator immediately about any emergency treatments, adverse events and additional medication taken. The terms and conditions of the insurance should be given to the subject.

## Agreements

### Financing of the trial

The trial is supported by restricted grants from AbbVie Inc. and Janssen Pharmaceutica NV.

The general conditions of financing for this trial are addressed in separate agreements.

### Report

After conclusion of the trial, the sponsor ensures that a report will be written according to the conditions stipulated in protocol. By signing this protocol, the investigators agree to disclose their names in the trial report.

As this trial is planned to be transitioned to EU-Regulation 536/2014, the sponsor should submit a summary of the results of the clinical trial according to Annex IV together with a summary that is understandable to a layperson according to Annex V and the clinical study report within 1 year after the end of the clinical trial to the CTIS.

### Publication policy

Any publication of the results, either in part or in total (articles in journals or newspapers, oral presentation, etc.) by the investigators, their representatives, or by the sponsor, shall require the approval of the Sponsor delegate.

It is planned to publish the results of the trial as an original article in an appropriate medical journal as well as present at congresses. The LKP/ Coordinating Investigator is first author of the primary publication and will first present the data at a major congress, last authorship will be coverd by the Sponsor’s delegate. The choice of the journal for the publication will be made by the PI and the Sponsor delegate in agreement with the co-authors. Besides the investigator, further authors of this article have to meet the following criteria:

- Substantial contribution to the recruitment of subjects, i.e. one of the five best recruiting sites within the trial.
- Substantial contribution to the interpretation of the data.
- Substantial contribution to drafting the article or revising it critically for important intellectual content.

# REFERENCES

1. Campo E, Rule S. Mantle cell lymphoma: evolving management strategies. Blood. 2015;125(1):48-55.

2. Hoster E, Dreyling M, Klapper W, Gisselbrecht C, van Hoof A, Kluin-Nelemans HC, et al. A new prognostic index (MIPI) for patients with advanced-stage mantle cell lymphoma. Blood. 2008;111(2):558-65.

3. Hoster E, Klapper W, Hermine O, Kluin-Nelemans HC, Walewski J, van Hoof A, et al. Confirmation of the Mantle-Cell Lymphoma International Prognostic Index in Randomized Trials of the European Mantle-Cell Lymphoma Network. Journal of Clinical Oncology. 2014;32(13):1338-46.

4. Hoster E, Rosenwald A, Berger F, Bernd HW, Hartmann S, Loddenkemper C, et al. Prognostic Value of Ki-67 Index, Cytology, and Growth Pattern in Mantle-Cell Lymphoma: Results From Randomized Trials of the European Mantle Cell Lymphoma Network. J Clin Oncol. 2016;34(12):1386-94.

5. Le Gouill S, Thieblemont C, Oberic L, Moreau A, Bouabdallah K, Dartigeas C, et al. Rituximab after Autologous Stem-Cell Transplantation in Mantle-Cell Lymphoma. N Engl J Med. 2017;377(13):1250-60.

6. Hermine O, Hoster E, Walewski J, Bosly A, Stilgenbauer S, Thieblemont C, et al. Addition of high-dose cytarabine to immunochemotherapy before autologous stem-cell transplantation in patients aged 65 years or younger with mantle cell lymphoma (MCL Younger): a randomised, open-label, phase 3 trial of the European Mantle Cell Lymphoma Network. Lancet. 2016;388(10044):565-75.

7. Le Gouill S, Thieblemont C, Oberic L, Moreau A, Bouabdallah K, Dartigeas C, et al. Rituximab after Autologous Stem-Cell Transplantation in Mantle-Cell Lymphoma. The New England journal of medicine. 2017;377(13):1250-60.

8. Dreyling M, Campo E, Hermine O, Jerkeman M, Le Gouill S, Rule S, et al. Newly diagnosed and relapsed mantle cell lymphoma: ESMO Clinical Practice Guidelines for diagnosis, treatment and follow-up. Annals of oncology : official journal of the European Society for Medical Oncology / ESMO. 2017;28(suppl_4):iv62-iv71.

9. Rummel MJ, Al-Batran SE, Kim SZ, Welslau M, Hecker R, Kofahl-Krause D, et al. Bendamustine plus rituximab is effective and has a favorable toxicity profile in the treatment of mantle cell and low-grade non-Hodgkin's lymphoma. J Clin Oncol. 2005;23(15):3383-9.

10. Robak T, Huang H, Jin J, Zhu J, Liu T, Samoilova O, et al. Bortezomib-Based Therapy for Newly Diagnosed Mantle-Cell Lymphoma. New England Journal of Medicine. 2015;372(10):944-53.

11. Robak T, Jin J, Pylypenko H, Verhoef G, Siritanaratkul N, Drach J, et al. Frontline bortezomib, rituximab, cyclophosphamide, doxorubicin, and prednisone (VR-CAP) versus rituximab, cyclophosphamide, doxorubicin, vincristine, and prednisone (R-CHOP) in transplantation-ineligible patients with newly diagnosed mantle cell lymphoma: final overall survival results of a randomised, open-label, phase 3 study. Lancet Oncol. 2018;19(11):1449-58.

12. Kluin-Nelemans HC, Hoster E, Hermine O, Walewski J, Geisler CH, Trneny M, et al. Treatment of Older Patients With Mantle Cell Lymphoma (MCL): Long-Term Follow-Up of the Randomized European MCL Elderly Trial. Journal of Clinical Oncology. 2020;38(3):248-56.

13. Kluin-Nelemans HC, Hoster E, Hermine O, Walewski J, Trneny M, Geisler CH, et al. Treatment of older patients with mantle-cell lymphoma. N Engl J Med. 2012;367(6):520-31.

14. Tam CS, Bassett R, Ledesma C, Korbling M, Alousi A, Hosing C, et al. Mature results of the M. D. Anderson Cancer Center risk-adapted transplantation strategy in mantle cell lymphoma. Blood. 2009;113(18):4144-52.

15. Dietrich S, Tielesch B, Rieger M, Nickelsen M, Pott C, Witzens-Harig M, et al. Patterns and outcome of relapse after autologous stem cell transplantation for mantle cell lymphoma. Cancer. 2011;117(9):1901-10.

16. Dreger P, Michallet M, Bosman P, Dietrich S, Sobh M, Boumendil A, et al. Ibrutinib for bridging to allogeneic hematopoietic cell transplantation in patients with chronic lymphocytic leukemia or mantle cell lymphoma: a study by the EBMT Chronic Malignancies and Lymphoma Working Parties. Bone Marrow Transplant. 2018.

17. Robinson SP, Boumendil A, Finel H, Peggs KS, Chevallier P, Sierra J, et al. Long-term outcome analysis of reduced-intensity allogeneic stem cell transplantation in patients with mantle cell lymphoma: a retrospective study from the EBMT Lymphoma Working Party. Bone Marrow Transplant. 2018;53(5):617-24.

18. Wang M, Rule S, Martin P, Goy A, Auer R, Kahl B, et al. Targeting BTK with Ibrutinib in Relapsed or Refractory Mantle-Cell Lymphoma. N Engl J Med. 2013;369(6):507 - 16.

19. Dreyling M, Jurczak W, Jerkeman M, Silva RS, Rusconi C, Trneny M, et al. Ibrutinib versus temsirolimus in patients with relapsed or refractory mantle-cell lymphoma: an international, randomised, open-label, phase 3 study. Lancet. 2016;387(10020):770-8.

20. Wang M, Schuster SJ, Phillips T, Lossos IS, Goy A, Rule S, et al. Observational study of lenalidomide in patients with mantle cell lymphoma who relapsed/progressed after or were refractory/intolerant to ibrutinib (MCL-004). J Hematol Oncol. 2017;10(1):171.

21. Hess G, Romaguera JE, Verhoef G, Herbrecht R, Crump M, Strahs A, et al. Phase III study of patients with relapsed, refractory mantle cell lymphoma treated with temsirolimus compared with investigator's choice therapy. Journal of Clinical Oncology. 2008;26(15).

22. Hess G, Keller U, Scholz CW, Witzens-Harig M, Atta J, Buske C, et al. Safety and efficacy of Temsirolimus in combination with Bendamustine and Rituximab in relapsed mantle cell and follicular lymphoma. Leukemia. 2015;29(8):1695-701.

23. Davids MS. Targeting BCL-2 in B-cell lymphomas. Blood. 2017;130(9):1081-8.

24. Wang M, Munoz J, Goy A, Locke FL, Jacobson CA, Hill BT, et al. KTE-X19 CAR T-Cell Therapy in Relapsed or Refractory Mantle-Cell Lymphoma. The New England journal of medicine. 2020;382(14):1331-42.

25. Tam CS, Anderson MA, Pott C, Agarwal R, Handunnetti S, Hicks RJ, et al. Ibrutinib plus Venetoclax for the Treatment of Mantle-Cell Lymphoma. N Engl J Med. 2018;378(13):1211-23.

26. Le Gouill S, Morschhauser F, Chiron D, Bouabdallah K, Cartron G, Casasnovas O, et al. Ibrutinib, Obinutuzumab And Venetoclax In Relapsed and Untreated Patients with Mantle-Cell Lymphoma, a phase I/II trial. Blood. 2020.

27. Wang ML, Jurczak W, Jerkeman M, Trotman J, Zinzani PL, Belada D, et al. Ibrutinib plus Bendamustine and Rituximab in Untreated Mantle-Cell Lymphoma. New England Journal of Medicine. 2022;386(26):2482-94.

28. Jain P, Zhao S, Lee HJ, Hill HA, Ok CY, Kanagal-Shamanna R, et al. Ibrutinib With Rituximab in First-Line Treatment of Older Patients With Mantle Cell Lymphoma. Journal of clinical oncology : official journal of the American Society of Clinical Oncology. 2022;40(2):202-12.

29. Rule S, Dreyling M, Goy A, Hess G, Auer R, Kahl B, et al. Ibrutinib for the treatment of relapsed/refractory mantle cell lymphoma: extended 3.5-year follow up from a pooled analysis. Haematologica. 2019;104(5):e211-e4.

30. Cheson BD, Fisher RI, Barrington SF, Cavalli F, Schwartz LH, Zucca E, et al. Recommendations for Initial Evaluation, Staging, and Response Assessment of Hodgkin and Non-Hodgkin Lymphoma: The Lugano Classification. Journal of Clinical Oncology. 2014;32(27):3059-67.

31. Cheson BD, Pfistner B, Juweid ME, Gascoyne RD, Specht L, Horning SJ, et al. Revised response criteria for malignant lymphoma. Journal of clinical oncology : official journal of the American Society of Clinical Oncology. 2007;25(5):579-86.

32. Aaronson NK, Ahmedzai S, Bergman B, Bullinger M, Cull A, Duez NJ, et al. The European-Organization-for-Research-and-Treatment-of-Cancer Qlq-C30 - a Quality-of-Life Instrument for Use in International Clinical-Trials in Oncology. Journal of the National Cancer Institute. 1993;85(5):365-76.

33. Husson O, Rooij BHd, Kieffer JM, Oerlemans S, Mols F, Aaronson NK, et al. Independent prognostic value of the EORTC QLQ-C30 summary score on all-cause mortality: Results from the population-based PROFILES registry. Journal of Clinical Oncology. 2018;36(15_suppl):10070-.

34. Wildiers H, Heeren P, Puts M, Topinkova E, Janssen-Heijnen ML, Extermann M, et al. International Society of Geriatric Oncology consensus on geriatric assessment in older patients with cancer. J Clin Oncol. 2014;32(24):2595-603.

35. Buske C, Hutchings M, Ladetto M, Goede V, Mey U, Soubeyran P, et al. ESMO Consensus Conference on malignant lymphoma: general perspectives and recommendations for the clinical management of the elderly patient with malignant lymphoma. Ann Oncol. 2018;29(3):544-62.

36. Bron D, Ades L, Fulop T, Goede V, Stauder R, Elderly Task Force in Hematology ES. Aging and blood disorders: new perspectives, new challenges. Haematologica. 2015;100(4):415-7.

37. Merli F, Luminari S, Tucci A, Arcari A, Rigacci L, Hawkes E, et al. Simplified Geriatric Assessment in Older Patients With Diffuse Large B-Cell Lymphoma: The Prospective Elderly Project of the Fondazione Italiana Linfomi. J Clin Oncol. 2021;39(11):1214-22.

38. Ye H, Desai A, Zeng D, Romaguera J, Wang ML. Frontline Treatment for Older Patients with Mantle Cell Lymphoma. Oncologist. 2018;23(11):1337-48.

39. Lister TA, Crowther D, Sutcliffe SB, Glatstein E, Canellos GP, Young RC, et al. Report of a committee convened to discuss the evaluation and staging of patients with Hodgkin's disease: Cotswolds meeting. Journal of clinical oncology : official journal of the American Society of Clinical Oncology. 1989;7(11):1630-6.

# APPENDICES

## ANNEX I SAFETY DEFINITIONS

**Adverse Event (AE)**

An Adverse Event (AE) is any untoward medical occurrence (i.e. any unfavorable and unintended sign [including abnormal laboratory findings], symptom or disease) in a subject or in a clinical investigation subject who has administered a medicinal or pharmaceutical product or is participating in a clinical study. Therefore, an AE may or may not be temporally or causally associated with the use of a medicinal (investigational) product.

This may include the following:

- AEs not previously observed in the patient that emerge during the protocol‑specified AE reporting period
- Complications that occur as a result of protocol-mandated interventions (e.g., invasive procedures such as biopsies)
- Preexisting medical conditions (other than FL), judged by the investigator to have worsened in severity or frequency or changed in character during the protocol‑specified AE reporting period

**Adverse (Drug) Reaction (AR)**

Adverse drug reactions (ADR) are all noxious and unintended responses to a medicinal product related to any dose. The phrase "responses to a medicinal product" means that a causal relationship between a medicinal product and an adverse event is at least a reasonable possibility, i.e., the relationship cannot be ruled out.

**Unexpected Adverse (Drug) Reaction (UAR)**

This is defined to be an adverse drug reaction which nature and severity is not consistent with the applicable product information (e.g. Summary of Product Characteristics for an authorized product or Investigator’s Brochures for an unauthorized investigational medicinal product), or an event which has not previously been observed or documented and which is thus not on the basis of what might be anticipated from the pharmacological properties of the product.

**Serious Adverse Event (SAE)**

A Serious Adverse Event is any untoward medical occurrence or effect at any dose, any undesirable or unintentional effect that:

- results in death (regardless of cause)
- is life threatening
- places the subject, in the view of the investigator, at immediate risk of death at the time of event
- It does not refer to an event that, which hypothetically might have caused death if it were more severe
- results in hospitalization (overnight stay) or prolongation of existing hospitalization, excluding the following:
- Hospitalization that does not necessitate an overnight stay.
- routine scheduled treatment or monitoring of the studied indication, not associated with any deterioration in condition
- Hospitalisation planned before subject entered in the study
- elective or pre-planned treatment for a pre-existing condition that is unrelated to the indication treated in the study and which has not worsened since the start of treatment with the investigational medicinal product
- results in persistent or significant disability or incapacity for the subject
- disability is a substantial disruption of a person’s ability to conduct normal life functions
- is associated with a congenital anomaly or birth defect
- is qualified as “other” important medically significant event or condition e.g. the event may jeopardize the subject or may require intervention to prevent one of the outcome listed above (e.g. intensive treatment in an emergency room or at home).

**Serious Adverse (Drug) Reaction (SAR)**

This is defined as an adverse drug reaction that is serious and at least possibly related to IMP (see SAE criteria above). The events that are excluded from the definition of an SAE are also excluded from the definition of an SAR.

**Suspected Unexpected Serious Adverse (Drug) Reaction (SUSAR)**

A SUSAR is an adverse reaction, which is both serious and unexpected because the nature or severity of this event is not consistent with the applicable product information (current version of german SmPc, Summary of Product Characteristics).

## ANNEX II Sample List of Cautionary Medications

Cautionary medications are defined as follows. Refer to section 6.2 on instructions for concomitant use of CYP3A inhibitors and inducers with Ibrutinib and Venetoclax, respectively.

The below medication apply to both Ibrutinib and Venetoclax unless otherwise specified.

| **INHIBITORS OR INDUCERS** | | **SUBSTRATES** |
| --- | --- | --- |
| **Strong CYP3A inhibitors:** | **Strong CYP3A inducers:** | **Substrates of P-gp** |
| boceprevir | avasimibe | aliskiren |
| clarithromycin | carbamazepine | ambrisentan |
| cobicistat | phenobarbitalPhenobarbital | colchicines |
| conivaptan | phenytoin | dabigatran etexilate |
| indinavir | rifabutin | digoxin |
| itraconazole | rifampin | everolimus |
| ketoconazole | St. John’s Wort | fexofenadine |
| lopinavir |  | lapatinib |
| mibefradil |  | loperamide |
| nefazodone | **Moderate CYP3A inducers:** | maraviroc |
| nelfinavir | bosentan | nilotinib |
| posaconazole | efavirenz | ranolazine |
| ritonavir | etravirine | saxagliptin |
| saquinavir | modafinil | sirolimus |
| telaprevir | nafcillin | sitagliptin |
| telithromycin | oxcarbazepine | talinolol |
| troleandomycin | troglitazone | tolvaptan |
| voriconazole* |  | topotecan |
| **Moderate CYP3A inhibitors:** | **Weak CYP3A inducers:** | **Substrates of BCRP**  **(Venetoclax only)** |
| aprepitant | amprenavir | methotrexate |
| amprenavir | aprepitant | mitoxantrone |
| atazanavir | armodafinil | irrinotecan |
| ciprofloxacin | clobazamechinacea | lapatinib |
| crizotinib | glucocorticoids (eg, prednisone) | rosuvastatin |
| darunavir/ritonavir | nevirapine | sulfasalazine |
| dronedarone | pioglitazone | topotecan |
| erythromycin | rufinamide |  |
| diltiazem | vemurafenib |  |
| fluconazole |  |  |
| fosamprenavir |  |  |
| imatinib |  |  |
| cverapamil |  |  |
| **Weak CYP3A inhibitors:** | **Inhibitors of OATP1B1/B3**  **(Venetoclax)** | **Substrates of OATP1B1/B3**  **(Venetoclax only)** |
| alprazolam | gemfibrozil | atrasentan |
| amiodarone | eltrombopag | atorvastatin |
| amlodipine | cyclosporine | ezetimibe |
| atorvastatin | tipranavir | fluvastatin |
| bicalutamide |  | glyburide |
| cilostazol |  | olmesartan |
| cimetidine | **Inhibitors of BCRP (Venetoclax )** | rosuvastatin |
| cyclosporine | cyclosporine | simvastatin acid |
| fluvoxamine | geftinib | pitavastatin |
| fluoxetine |  | pravastatin |
| ginkgo |  | repaglinide |
| goldenseal | **Inhibitors of P-gp (Venetoclax)** | telmisartan |
| isoniazid | amiodarone | valsartan |
| nilotinib | azithromycin |  |
| oral contraceptives | captopril |  |
| pazopanib | carvedilol |  |
| ranitidine | cyclosporine |  |
| ranolazine | dronedarone |  |
| suboxone | felodipine |  |
| tipranavir/ritonavir | quercetin |  |
| ticagrelor | quinidine |  |
| zileuton | ranolazine |  |
|  | ticagrelor |  |

* Allowed to dose with 140 mg ibrutinib based on clinical data

Note that this is not an exhaustive list. Further information can be found at the following websites:

http://medicine.iupui.edu/clinpharm/ddis/main-table/ and

http://www.fda.gov/Drugs/DevelopmentApprovalProcess/DevelopmentResources/DrugInteractionsLabeling/ucm080499.htm.

In addition to the medications listed in this table, subjects receiving venetoclax should not consume grapefruit, grapefruit products, Seville oranges (including marmalade containing Seville oranges) or starfruits.

## ANNEX III Lugano classification for staging of lymphomas (derived from Ann Arbor staging with Cotswolds modifications)

**Stage I** — Involvement of a single lymph node region (e.g., cervical, axillary, inguinal, mediastinal) or lymphoid structure such as the spleen, thymus, or Waldeyer's ring.

**Stage II** — Involvement of two or more lymph node regions or lymph node structures on the same side of the diaphragm. Hilar nodes should be considered to be "lateralized" and when involved on both sides, constitute stage II disease. For the purpose of defining the number of anatomic regions, all nodal disease within the mediastinum is considered to be a single lymph node region, and hilar involvement constitutes an additional site of involvement. The number of anatomic regions should be indicated by a subscript (e.g., II-3).

**Stage III** — Involvement of lymph node regions or lymphoid structures on both sides of the diaphragm. This may be subdivided stage III-1 or III-2: stage III-1 is used for patients with involvement of the spleen or splenic hilar, celiac, or portal nodes; and stage III-2 is used for patients with involvement of the paraaortic, iliac, inguinal, or mesenteric nodes.

**Stage IV** — Diffuse or disseminated involvement of one or more extranodal organs or tissue beyond that designated E, with or without associated lymph node involvement.

All cases are subclassified to indicate the absence (A) or presence (B) of the systemic symptoms of significant unexplained fever, night sweats, or unexplained weight loss exceeding 10% of body weight during the six months prior to diagnosis.

The designation "E" refers to extranodal contiguous extension (i.e., proximal or contiguous extranodal disease) that can be encompassed within an irradiation field appropriate for nodal disease of the same anatomic extent. More extensive extranodal disease is designated stage IV.

Bulky disease: A single nodal mass, in contrast to multiple smaller nodes, of 10 cm or ≥⅓ of the transthoracic diameter at any level of thoracic vertebrae as determined by CT; record the longest measurement by CT scan. The term "X" (used in the Ann Arbor staging system) is no longer necessary.

The subscript "RS" is used to designate the stage at the time of relapse.

Patients can be clinically or pathologically staged. Splenectomy, liver biopsy, lymph node biopsy, and/or bone marrow biopsy are mandatory for the establishment of pathological stage. The pathologic stage at a given site is denoted by a subscript (eg, M = bone marrow, H = liver, L = lung, O = bone, P = pleura, and D = skin).

Adapted from Lister et al, 1989 (39) and Cheson et al, 2014 (30).
